# Comparison of face attention bias in adults with ASD, ADHD, or comorbid ADHD+ASD

**DOI:** 10.1093/scan/nsaf112

**Published:** 2025-10-27

**Authors:** Irene Sophia Plank, Julia Nowak, Alexandra Pior, Christine M Falter-Wagner

**Affiliations:** Department of Psychiatry and Psychotherapy, LMU University Hospital, LMU Munich, Munich 80336, Germany; Department of Psychiatry and Psychotherapy, LMU University Hospital, LMU Munich, Munich 80336, Germany; Department of Sociology, Faculty of Behavioral and Social Sciences, University of Groningen, Groningen 9712 TS, Netherlands; Department of Psychology, Faculty of Psychology and Neuroscience, Maastricht University, Maastricht 6229 ER, Netherlands; Department of Psychiatry and Psychotherapy, LMU University Hospital, LMU Munich, Munich 80336, Germany

**Keywords:** face attention bias, autism, attention-deficit/hyperactivity disorder, attention, social attention

## Abstract

Faces are special for humans. This importance is reflected in increased relative attention to faces, referred to as face attention bias (FAB). This preregistered study investigated FAB transdiagnostically in two neurodevelopmental disorders associated with social symptoms, autism spectrum disorder (ASD) and attention-deficit/hyperactivity disorder (ADHD). We assessed exogenous selective attention to faces using a dot-probe paradigm in adults with ASD, adults with ADHD, adults with both and non-clinical comparison adults. While comparison adults showed FAB as expected, adults with ASD did not. Yet, the between-group difference in FAB was not credible, contrary to our hypothesis. Critically, adults with ADHD but no ASD showed *increased* FAB, suggesting heightened exogenous selective attention towards faces. This increase was not reflected in oculomotor behaviour, indicating covert attentional mechanisms. Adults with comorbid ASD and ADHD did not show increased FAB. Saccades were produced faster towards face-cued targets across all groups, but no significant interaction with group emerged regarding oculomotor behaviour. These findings unveil an attentional signature in ADHD: a heightened bias for faces possibly connected to social symptoms. Furthermore, they highlight the nuanced and distinct attentional profiles in different neurodevelopmental disorders, underscoring the critical need to understand shared and distinct mechanisms of ASD and ADHD.

## Introduction

Neurodevelopmental disorders are a group of disorders emerging early during development and persisting across the lifespan (Morris-Rosendahl and Crocq 2020). Among these disorders, autism spectrum disorder (ASD) and attention-deficit/hyperactivity disorder (ADHD) show overlap in genetic vulnerability and behavioural phenomenology (Antshel and Russo 2019, Lau-Zhu et al. 2019), resulting in an estimated 28% of autistic adults having a comorbid ADHD diagnosis (Lai et al. 2019). These commonalities exist over and above the defining symptomatology specific to ASD and ADHD, respectively. ASD is being diagnosed based on difficulties in social interaction and communication in addition to repetitive and restricted behaviour, while ADHD is characterized by difficulties regulating attention with or without hyperactivity and impulsivity (World Health Organization 2019). Investigations of the overlap of ADHD and ASD with respect to those selective symptoms and carving out underlying mechanisms thereof could not only refine our mechanistic understanding of both disorders but also guide interventions (Antshel and Russo 2019). In the current study, we focused on social attention as a vital base for social cognition for which attenuation have been demonstrated in both ADHD and ASD (Borhani and Nejati 2018, Yeung 2022). While impairments in social cognition are part of the core symptoms of ASD, the cause of these impairments in ADHD is still discussed, specifically whether they are a consequence of attention deficits or not (Roberts and Trossman 2024). Comparable social attention impairments in ASD and ADHD could provide evidence for a shared mechanism across neurodevelopmental disorders, supporting a domain-specific social attention impairment going beyond domain-general attention deficits in ADHD. Thus, in this study, we focus on the transdiagnostic overlap of social attention in ASD and ADHD to capture modified social attention behaviourally but also explore mechanistic differences behind these modifications.

Attention can be divided into selective and sustained attention, where the first allows the preferential processing of a stimulus and the latter allows us to continuously perform a task (Mueller et al. 2017). We focus here on selective attention to social stimuli; that is how social stimuli are preferentially capturing attention. Attention to stimuli can be top-down, endogenously driven, e.g. by certain task goals, or bottom-up, exogenously driven, for instance by bright colours (Mueller et al. 2017). In a computational model proposed by Wickens (2021), four factors predict which stimuli capture attention: salience, effort, expectancy and value. Of these factors, we focus on the salience of social stimuli, specifically faces. On the neuronal level, research links dopamine to the processing of perceived salience (Chen and Bruchas 2021, Kutlu et al. 2021, Fleury et al. 2024). Furthermore, studies found a modulatory effect of oxytocin on social attention (Dal Monte et al. 2017, Kanat et al. 2017, Burley and Daughters 2020, Le et al. 2021, Zhuang et al. 2022). Both findings are linked in the social salience hypothesis of oxytocin. According to Shamay-Tsoory and Abu-Akel (2016), oxytocin unfolds its effects on social behaviour and cognition by regulating the salience of social cues via the dopaminergic system. Alerting signals are first sent to salience-coding dopaminergic neurons in the ventral tegmental area and from there to mesolimbic structures to assess value and valence. Following their hypothesis, the effect of oxytocin depends on changes in dopamine.

The dot-probe paradigm allows to manipulate exogenous attention driven by salience, e.g. for social stimuli like faces to assess social attention. Participants encounter targets which are preceded by cues: reaction to targets is faster if the cue which was presented at the location of the target captured attention. Assuming balanced conditions, performance is independent of whether someone attends certain cues preferentially. Therefore, effort, expectancy and value are constant, only salience differs between the conditions leading to effects being driven by exogenous attention. Previous studies have shown decreased reaction times and altered oculomotor behaviour in response to face- compared to object-cued targets (Bindemann et al. 2007, Moore et al. 2012, Pereira et al. 2020, 2022, Jakobsen et al. 2021), indicating increased salience resulting in exogenous attention to faces (face attention bias, FAB). This preferential attention to faces has also been shown in a variety of other contexts, for instance when faces are competing with other stimuli (Ro et al. 2001), pop out amongst other stimuli (Hershler and Hochstein 2005), or retain our attention more strongly (Bindemann et al. 2005). The peculiarity of the dot-probe task to assess FAB is that both objects and faces are irrelevant to the task and, thus, FAB measures salience of faces compared to objects independent of task performance. Therefore, we captured both response and oculomotor behaviour in a dot-probe paradigm developed by Jakobsen et al. (2021) to assess exogenous attention to faces in the current study.

An important symptom of autism is attenuated eye contact, e.g. avoidance or asynchronous eye contact during conversations (World Health Organization 2019). This observation has inspired research to investigate social attention with eye-tracking studies. A meta-analysis found reduced dwell times for faces, eyes and mouths as well as non-social stimuli in autistic adults (Setien-Ramos et al. 2023). Differences seem to be most pronounced for the eye region, with medium effect sizes for reduced number of fixations and decreased fixation duration (Cohen 1988). Specifically focusing on exogenous attention to faces, Moore et al. (2012) used a dot-probe paradigm with a cue presentation of 200 ms to assess FAB in autistic and non-autistic adults. They observed a significant FAB in non-autistic but not autistic adults. In a similar paradigm, Kanat et al. (2017) found reduced FAB in autistic adults when presenting face and object cues for 500 ms but not when presenting them for 100 ms. Their study also provided further evidence for the regulatory effect of oxytocin on the salience of faces: they found administering intranasal oxytocin before performing a dot-probe paradigm enhanced attention to faces overall and specifically in the autistic adults for longer cue presentations. These findings suggest decreased FAB in autistic adults as measured by a dot-probe paradigm where the cue is presented between 200 and 500 ms. While these are the only studies using the dot-probe paradigm to investigate FAB in ASD, other paradigms have demonstrated a decrease across the lifespan. For instance, individuals with ASD have less difficulties disengaging from faces (Chawarska et al. 2010), show reduced dwell times to eyes and faces (Setien-Ramos et al. 2023) and may be less distracted by task-irrelevant faces (Remington et al. 2012). However, faces interfered more than objects with the performance of autistic individuals in a Stroop task (Ashwin et al. 2006) and were easier to find than objects in a face-in-the-crowd paradigm (Moore et al. 2016).

Less research has focused on social attention in ADHD, despite the emergence of literature on social interaction (Gvirts Problovski et al. 2021) and facial emotion recognition deficits (Borhani and Nejati 2018). Social attention could disentangle the causes for these social deficits, distinguishing between social deficits due to core ADHD symptoms, specifically attention deficits, or as a separate mechanism (Roberts and Trossman 2024). Muszkat et al. (2015) found shorter dwell times to eyes in a free viewing paradigm in both children with ADHD or ASD compared with children without psychiatric diagnoses. Nagatsuka et al. (2024) found no differences in dwell times in adults with or without ADHD when watching social scenes from movies. In a direct comparison of ADHD and ASD in children, Kochhar et al. (2024) found dwell times to be reduced and first fixation time increased in autistic children regardless of ADHD diagnosis and no differences in gaze patterns associated with ADHD. While there is no research on FAB for faces in adults with ADHD to date, research on attentional biases in individuals with ADHD has revealed both increased attention biases for emotional over neutral faces (Raz and Dan 2015, Cremone et al. 2018, Nejati and Estaji 2024) as well as decreased value-driven attentional capture, gaze-cueing and attention bias to alcohol-related images (Roberts et al. 2012, Sali et al. 2018, Uono et al. 2023). Thus, we expect FAB to be influenced by ADHD but consider both increased FAB and decreased FAB to be possible, indicating increased and decreased salience of faces, respectively. General distractibility and domain-general attention deficits do not influence FAB as they would affect both cue types. Thus, both increased and decreased FAB in ADHD would support the theory of domain-specific social attention modifications, possibly contributing to social deficits. In the case of decreased FAB, social attention deficits could be a transdiagnostic symptom shared with ASD, while increased FAB would hint towards separate mechanisms in these two neurodevelopmental disorders.

Mechanistic explanations of attentional symptoms in ADHD have focused on the interplay of catecholamines, most notably dopamine, and attentional neural networks (Aboitiz et al. 2014, Mueller et al. 2017). Specifically, Aboitiz et al. (2014) propose a combination of disbalance in the catecholaminergic system and network interactions to explain why people with ADHD are more easily distracted by salient stimuli. Thus, dopamine imbalance affecting the perceived salience of stimuli might explain deficits in selective attention in ADHD. While theories of ASD focus less on endocrine factors, there are proposals focusing on the effect of oxytocin on dopaminergic areas of the brain during development (Havranek et al. 2024) as well as on dysfunction of the catecholaminergic system (Koevoet et al. 2022). Indeed, Koevoet et al. (2022) review the neuromodulation in ASD and ADHD to conclude that not only could dysfunction of catecholaminergic and cholinergic neuromodulation explain symptoms in both ASD and ADHD but more importantly that there seems to be relevant overlap in the type of modification between both neurodevelopmental disorders. If we observe a similarly decreased FAB in people with ASD and ADHD, with possibly additive effects of comorbidity, this could suggest a transdiagnostic effect of catecholaminergic imbalance affecting the salience of faces in both neurodevelopmental disorders.

In the present preregistered study, we investigated social attention by assessing FAB measured with a dot-probe paradigm in adults with ADHD and ASD. We expected to replicate FAB in the form of decreased reaction times to face-cued targets as well as an increased number of saccades towards the faces in our comparison group of adults without psychiatric diagnoses. Furthermore, we expected to replicate increased overall reaction times in adults with ASD and ADHD (Sonuga-Barke et al. 2004, Ghosn et al. 2019). Based on previous data, we hypothesized that FAB is reduced in autistic adults compared to adults without any psychiatric diagnoses (Moore et al. 2012, Kanat et al. 2017). Furthermore, we hypothesized that FAB in adults with ADHD differs from FAB in adults without psychiatric diagnoses. Concerning oculomotor behaviour, we hypothesize that decreased attention to faces in autistic adults results in increased latencies compared to adults of the comparison group when producing saccades towards face-cued targets. Additionally, we expected more saccades towards faces to be associated with increased FAB. Last, we collected data from a group of adults with ASD and ADHD to explore the effect of comorbid diagnoses. We used two questionnaires to collect clinical self-assessments of symptom severity in ASD and ADHD (Kessler et al. 2005, Rausch et al. 2024).

## Method

The preregistration of this study can be found on OSF: https://osf.io/bze4g. We performed the data preprocessing and analysis in R *4.4.1* (R Core Team 2021) in RStudio (RStudio Team 2020) as well as preprocessed the eye-tracking data and presented the paradigm in MATLAB *R2023a* (The Mathworks Inc. 2022) using Psychtoolbox-3 (Brainard 1997, Kleiner et al. 2007). All code as well as preprocessed anonymized data is shared on GitHub: https://github.com/IreneSophia/EMBA_FAB. Variances are reported as standard errors and square brackets are used to denote 95% intervals.

### Sample

We recruited 108 adult participants for this project. Participants were recruited by posting flyers online, at the LMU University Hospital Munich and distributing them via psychotherapist practices, as well as through the lab’s participant database. All participants received either monetary compensation or student credit for their time. The Ethics committee of the Medical Faculty of the LMU Munich approved this study (reference: 22-0561). We asked 45 of our autistic participants (both ASD and ADHD+ASD) for their language preference with mixed results (identity-first: 40%; person-first: 38%; no preference: 22%); thus, we use both throughout this manuscript.

We recruited autistic participants without ADHD (ASD group), autistic participants with ADHD (ADHD+ASD group), participants with ADHD but no ASD (ADHD group) as well as a comparison group (COMP group). We verified diagnoses by checking medical documents. Additionally, most of our autistic sample was recruited from our internal database for which participants’ diagnosis is validated by a trained clinician. Inclusion criteria for all groups were as follows: between 16 and 45 years of age, no neurological diagnoses, sufficient knowledge of the German language (determined by the experimenter either prior to testing on the phone or at the time of testing through a conversation as well as a verbal IQ task; MWT-B, Lehrl 1999), estimated IQ of at least 70, unimpaired or corrected-to-normal vision, written informed consent of the participant. One participant was underage at the time of testing; thus, both they and their parents gave written informed consent. Since only one participant was underage, we refer to the participants as adults throughout the manuscript. Participants in the ASD, ADHD and COMP group needed to be able to enter the MRI scanner. Participants in the COMP group had no psychiatric diagnoses and were not taking any psychotropic medication. Additional exclusion criteria were self- or other-harming behaviour and acute suicidality as assessed with the Beck’s Depression Inventory (BDI, Hautzinger et al. 1994).

We excluded the dataset of one autistic participant due to suicidality and three datasets due to a change in diagnostic status (two autistic, one ADHD+ASD). Furthermore, we excluded one participant with ADHD and one with ASD due to accuracies below 2/3 in the task of interest. Our final behavioural sample consisted of 23 participants with ADHD (56.5% on ADHD medication), 23 participants with ASD, 22 participants with ADHD+ASD (59.09% on ADHD medication) and 24 COMP participants (see Table 1). We included participants on ADHD medication to capture effects as they appear in everyday life, which for many adults with ADHD includes medication. The final sample size for the reaction time and error rate analyses slightly exceeded the minimal sample size of 22 participants per group estimated using PANGEA v0.2 (Westfall 2016) as follows: medium sized effect of *d *= 0.45 between two of the three preregistered groups (ASD, ADHD and COMP) with at least 80% power in each of the tasks used in this project (current task: 89.3% power; fixed effects: group, target cue; random effects: subjects nested in group, trials nested in target cue). However, additional exclusion of data for the eye tracking analysis results in a smaller sample than originally planned (16 ADHD, 19 ASD, 21 ADHD+ASD, and 21 COMP). Analysis of the ADHD+ASD group was explorative.

**Table 1.** Comparison of the four groups on clinical self-assessment questionnaires and socio-demographic measurements including means and standard errors or counts

Measurement	ADHD	ADHD+ASD	ASD	COMP	bf.log
ASRS-v1.1	43.39 ± 0.54 (16 to 62)	46.64 ± 0.43 (32 to 67)	32.83 ± 0.35 (14 to 52)	25.04 ± 0.34 (13 to 42)	19.997^*^
Age	26.70 ± 0.32 (17 to 43)	30.68 ± 0.37 (17 to 44)	29.04 ± 0.30 (20 to 45)	27.42 ± 0.24 (21 to 42)	−1.239
Education^a^	3.55 ± 0.05 (1 to 5)	4.00 ± 0.06 (1 to 5)	3.65 ± 0.04 (2 to 5)	4.33 ± 0.03 (2 to 5)	0.196
Gender^b^	9—12—2	12—7—3	12—11—0	11—13—0	−4.021
IQ estimate	108.35 ± 0.51 (84 to 130)	113.23 ± 0.52 (91 to 133)	111.98 ± 0.63 (78 to 144)	109.90 ± 0.39 (92 to 131)	−2.014
RADS-R	92.61 ± 1.82 (18 to 148)	145.86 ± 1.55 (85 to 201)	151.61 ± 1.79 (55 to 217)	44.58 ± 1.46 (9 to 142)	29.577^*^

Group differences concerning continuous measurements were assessed using Bayesian ANOVA either on the raw normal-distributed or rank-transformed variables. For gender distribution, we used contingency tables. Reported Bayes Factors (BF) are log-transformed so that positive values indicate evidence in favour and negative values evidence against group differences.

ADHD, adult attention-deficit/hyperactivity disorder; ASD, autism spectrum disorder; ASRS, Adult ADHD Self-Report Scale; COMP, comparison group of adults without psychiatric diagnoses; IQ, estimated Intelligence Quotient from verbal and non-verbal IQ; RADS-R, Ritvo Autism Diagnostic Scale—Revised.

a Education: ranging from 1 = “no educational or vocational training” to 5 = “University degree.”

b Gender categories: female—male—diverse/agender/nonbinary; asterisks highlight at least moderate evidence for a group difference as assessed by a Bayesian ANOVA.

* Asterisks denote credible evidence in favour of group differences.

### Experimental procedure

This study was part of a larger project that also included neuroimaging; however, the here-presented data was not collected in the fMRI scanner. Thus, testing was performed at the NeuroImaging Core Unit Munich (NICUM) (https://www.en.nicum.uni-muenchen.de/index.html). Participants were asked for their written informed consent before filling out a demographics questionnaire and the Beck Depression Inventory (BDI, Hautzinger et al. 1994) to assess suicidality followed by the Mehrfachwahl-Wortschatz-Intelligenztest (MWT-B, Lehrl 1999) and the Grundintelligenztest Skala 2 (CFT-20R, Weiß 2006). We computed the average of both intelligence measures to assess the inclusion criterion of IQ estimate above 70.

Then, they performed three behavioural tasks, including the dot-probe task to measure FAB. The order of the tasks was counterbalanced across participants and all tasks were performed in a separate soundproof chamber with consistent lighting conditions. The other tasks measured emotion discrimination threshold (Plank et al. 2025a,b) and probabilistic associative learning (Plank et al., in prep.). We collected their gaze patterns using a LiveTrack Lightning eye tracker (Cambridge RS, 500 Hz) and a headrest to ensure a constant viewing distance of 57 cm. All tasks were performed on a Lenovo Legion Pro 5 laptop (resolution 2560 × 1600 pixel, screen size 344 × 215mm, refresh rate 60 Hz). Before each task, an individual calibration of the eye tracker was performed based on nine points. We only collected data from eyes for which the accuracy was below 0.5°. Participants provided their answers using an external numpad placed on their preferred side.

Following the behavioural tasks, some participants performed a task in the fMRI scanner (Plank et al. 2025a,b). Participants also filled out the Ritvo Autism Diagnostic Scale—Revised (RADS-R, Rausch et al. 2024) and the Adult ADHD Self-Report Scale (ASRS-v1.1, Kessler et al. 2005) as clinical self-assessments of symptom severity. These questionnaires were filled out whenever participants needed a break, either between two of the behavioural tasks, after the behavioural tasks or at the end of testing. For most participants total testing duration was between 3 and 4 hours of which one hour was spent in the scanner. The here-presented dot-probe task took approximately 10 minutes.

### Dot-probe task

**Figure 1.**
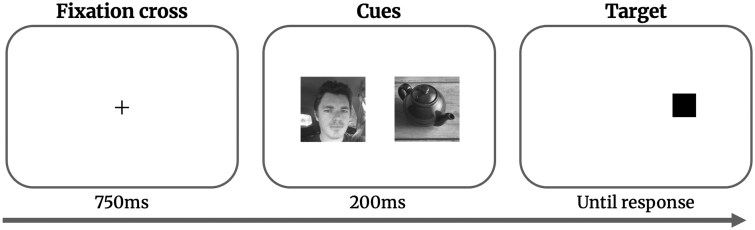
Schematic of the dot-probe task. In each trial, a pair of pictures consisting of one picture of a face and one picture of an object were displayed as the cue. This cue was presented for 200 ms and followed by a target square, which was presented until an answer was given. Only the middle of the screen is shown in this schematic. Stimuli have been reused with permission from Jakobsen et al. (2021).

We used a dot-probe task adapted from Jakobsen et al. (2021) to measure FAB. Trials started with the presentation of a centred fixation cross for 750 ms (see Fig. 1), followed by 200 ms of the cues, which were two quadratic pictures, one of an object and one of a face. These pictures were presented 5.34° apart centred around the fixation cross, with a visual angle of 4.25°. After the cues disappeared, a target square appeared at the location of one of the cues (height = width = 1.17° visual angle). In a two-answer forced-choice (2-AFC) task participants indicated the location of the target square as fast and accurately as possible. The target square stayed on screen until an answer was provided and participants received no accuracy feedback.

We used the same stimuli as Jakobsen et al. (2021) comprising six objects and six faces that were combined to create 36 picture pairs. All pairs were presented in two variants based on the location of the face (left or right). The stimuli were processed with the SHINE toolbox to match the luminance (Willenbockel et al. 2010). The task consisted of 432 trials, balanced across the following conditions: face location (left or right) and condition (target appeared on the side of the face or the object). Trials were pseudo-randomized within three blocks of 144 trials (72 cue pairs in two conditions) so that the target square never appeared on the same side in more than three consecutive trials. The order of blocks was randomized.

### Preprocessing

**Figure 2.**
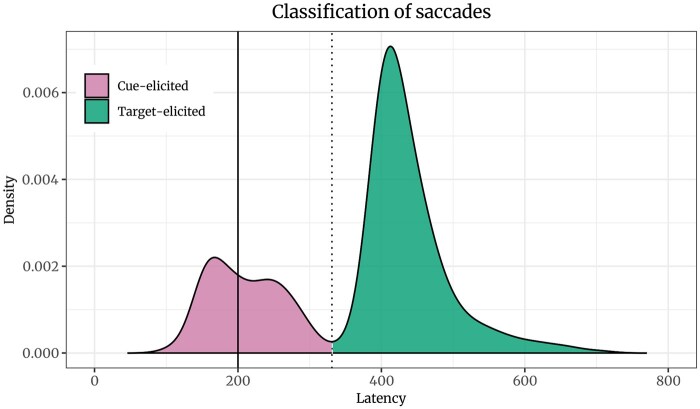
Density plot of saccade latency. The black line shows the onset of the target, 200 ms after the onset of the cue. The dotted line shows the critical point between saccades associated with the cue presentation in pink and saccades associated with the target presentation in green.

We used the interquartile range method to detect outliers in the reaction times and exclude them as well as incorrect trials from the analysis. Participants with less than 66.67% accuracy were excluded from the analysis (one with ADHD and one with ASD). We used the algorithm developed by Nyström and Holmqvist (2010) to detect saccades in participants’ eye movements based on the mean of both eyes (Cui and Hondzinski 2006). Participants who blinked on more than 1/3 of the samples were excluded from the eye-tracking analysis, which affected two of the 84 participants for whom eye-tracking data was collected. Only detected saccades starting during the presentation of the cue or target and starting at the previous location of the fixation cross, that is the centre of the screen between the cues, were included in the further analysis. Data of one autistic and one comparison participant was excluded since they did not have any relevant saccades. Relevant saccades were classified based on whether their direction was towards the location of the face cue and whether their direction was towards the target. Furthermore, we determined whether saccades were elicited with the presentation of the cue or with the presentation of the target. To do so, we calculated the critical point of the density function of the latencies (see Fig. 2).

### Analyses

We used Bayesian linear mixed models (BLMM) as implemented in the brms package (Bürkner 2017) to test our hypotheses with effects with a posterior probability above 95% for directed hypotheses and 97.5% for undirected hypotheses described as credible. All categorical predictors were set to sum contrast coding. All models included the population-level effects group (ADHD, ASD, ADHD+ASD, and COMP) as well as target cue (face-cued target and object-cued target) or direction for cue-induced saccades (face or object cue). Models assessing reaction times and latencies were based on shifted lognormal distributions and models assessing number of saccades were modelled with a Poisson distribution. Furthermore, we used a Bayesian spearman correlation to assess the relationship between FAB and number of saccades towards faces (van Doorn and Ly 2017).

We followed Barr’s guidelines (Barr et al. 2013) regarding group-level effects (participant for all models, additionally stimulus for models with the outcome reaction time). We extensively tested the validity and reliability of our BLMMs following the suggestions by Schad et al. (2021), with some of these checks performed including three instead of four groups (see Supplementary Materials). Based on posterior predictive checks, we aggregated reaction times by computing the median reaction time for each cue pair in each target cue condition of all correctly answered trials as well as latencies by computing the median latency for each cue condition.

In addition to our hypotheses, we explored error rates (BLMM with Bernoulli distribution) as well as number, latencies and dwell times of cue-elicited saccades (BLMM with Poisson and shifted lognormal distribution). We also explored associations between FAB and autism-like traits as well as ADHD symptoms across groups using Bayesian Spearman correlations. Furthermore, we used Bayesian ANOVAs to explore the influences of female and male gender, group and their interaction on FAB as well as ADHD medication within the adults with ADHD. Last, we used Bayesian ANOVAs to perform group comparisons on clinical self-assessment questionnaires. The data was rank-transformed if not normally distributed. We consider Bayes Factors (BF) above 3 to be credible (for log-transformed BF this threshold corresponds to ∼1.099).

## Results

### Clinical self-assessment of symptom severity

We found credible differences between our groups in both of our clinical self-assessment questionnaires (see Table 1). Post-hoc tests revealed that clinical groups received higher scores on the RADS-R measuring autism-like traits than the COMP group (ADHD: log(*BF_10_*) = 5.393; ADHD+ASD: log(*BF_10_*) = 22.505; ASD: log(*BF_10_*) = 21.815). Furthermore, the ADHD+ASD group and the ASD group both scored higher than the ADHD group (ADHD+ASD: log(*BF_10_*) = 6.432; ASD: log(*BF_10_*) = 6.906) but did not differ from each other (log(*BF_10_*) = −1.116). Furthermore, all clinical groups scored higher on the ASRS-v1.1 screening tool for ADHD than the COMP group (ADHD: log(*BF_10_*) = 10.42; ADHD+ASD: log(*BF_10_*) = 17.476; ASD: log(*BF_10_*) = 2.887). While there was no credible difference between the ADHD and the ADHD+ASD group (log(*BF_10_*) = −0.835), both scored higher than the ASD-only group (ADHD: log(*BF_10_*) = 3.180; ADHD+ASD: log(*BF_10_*) = 8.253). Only one of our COMP participants scored above the threshold in the ASRS-v1.1 and only three above the threshold in the RADS-R, following the criteria described in Kessler et al. (2005) and Rausch et al. (2024). However, eleven of our ADHD participants and five of our ASD participants scored above the threshold in both (see S1.4).

### Task-relevant response behaviour

#### Reaction times

**Figure 3.**
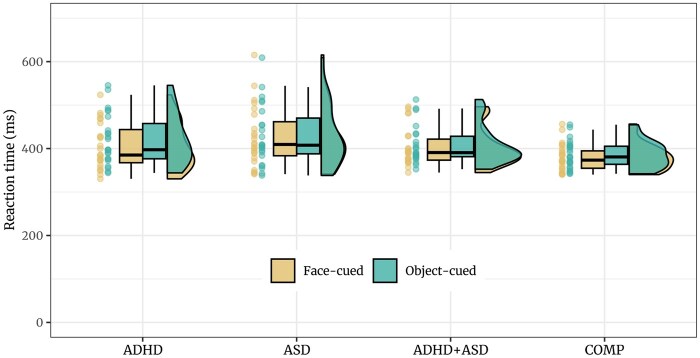
Median reaction times of correctly answered trials per subject for face and object cues. Boxplot show the median (bold line) as well as first and third quartiles (hinges). The whiskers extend from the hinges to the most extreme value no further than 1.5 times the interquartile range from the hinge. The clinical groups exhibited increased overall reaction times compared to adults without psychiatric diagnosis.

As hypothesized, both autistic adults and adults with ADHD exhibited increased overall reaction times compared with the COMP group (COMP–ADHD: *estimate *=* −*0.12 [*−*0.2, *−*0.03], *posterior probability *= 99.01%; COMP—ASD: *estimate *=* −*0.14 [*−*0.23, *−*0.06], *posterior probability *= 99.48%; see Fig. 3 and Table 2).

**Table 2.** Mean and standard error for reaction times (RT, only correct trials) and error rates of each group in each condition, showing similar error rates across groups and conditions

	ADHD		ASD		ADHD+ASD		COMP	
	Face-cued	Object-cued	Face-cued	Object-cued	Face-cued	Object-cued	Face-cued	Object-cued
Accuracy	3.38 ± 0.97	3.58 ± 0.89	2.98 ± 0.57	2.17 ± 0.39	2.19 ± 0.36	2.17 ± 0.43	2.16 ± 0.41	2.28 ± 0.43
RT	412 ± 12	425 ± 13	432 ± 16	437 ± 16	408 ± 11	414 ± 10	382 ± 7	389 ± 7

**Figure 4.**
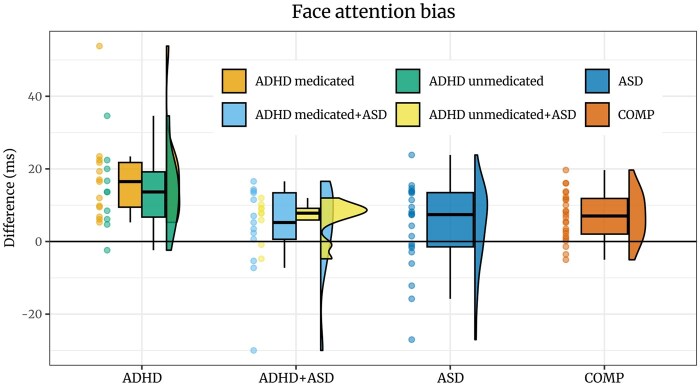
FAB for each participant, based on the difference between reaction time for targets appearing on the side of the object and targets appearing on the side of the face. While most participants in all groups seem to exhibit FAB, several autistic participants and some of the ADHD+ASD participants seem to exhibit an object attention bias instead. We plot medicated and unmedicated participants with ADHD separately.

Furthermore, COMP participants reacted faster in response to face-cued than object-cued targets (*estimate *=* −*0.03 [*−*0.06, *−*0.01], *posterior probability *= 98.52%). FAB was not credibly decreased in ASD participants compared to COMP participants (*estimate *=* −*0.02 [*−*0.04, 0.01], *posterior probability *= 87.52%). However, FAB was credibly higher in the ADHD than the COMP group (*estimate *= 0.03 [0, 0.06], *posterior probability *= 98.49%; see Fig. 4). While reaction times to both object- and face-cued targets were increased in ADHD compared to COMP participants (face-cued: *estimate *=* −*0.1 [*−*0.2, 0], *posterior probability *= 97.8%; object-cued: *estimate *=* −*0.13 [*−*0.23, *−*0.03], *posterior probability *= 99.48%), this increase was larger for the object-cued targets (*estimate *= 0.03 [0, 0.06], *posterior probability *= 98.49%, see S2.2). Comparison of FAB in percentage of participant-specific reaction times confirmed that increased FAB in ADHD was not merely due to overall increased reaction times (see S4.3). Exploration revealed a credible main effect of cue, suggesting a FAB effect when considering data from all groups (*estimate *=* −*0.03 [*−*0.06, *−*0.01], *posterior probability *= 99.74%; see Table 3) as well as separately in the ADHD group (*estimate *=* −*0.06 [*−*0.09, *−*0.04], *posterior probability *= 100%) but not the ASD (*estimate *=* −*0.02 [*−*0.05, 0.01], *posterior probability *= 88.75%) or the ADHD+ASD group (*estimate *=* −*0.02 [*−*0.05, 0.01], *posterior probability *= 94.42%). A similar model including the number of saccades produced the same results (see S3). Furthermore, Bayesian Spearman correlations revealed moderate evidence against associations between FAB and questionnaires assessing ASD (RADS-R: log(*BF*) = *−*1.72) or ADHD (ASRS: log(*BF*) = *−*1.59; see S4.1) as well as against an association between FAB and overall reaction times (log(*BF*) = *−*1.31). A Bayesian ANOVA including 19 adults not taking medication and 26 adults taking medication revealed credible evidence against an effect of ADHD medication on FAB in ADHD and ADHD+ASD adults (log(*BF*) = *−*1.21). Last, we found no indication of an effect of gender on FAB (see S4.2).

**Table 3.** Summary of FAB measures

Measurement	Object-cued target	Face-cued target	Difference
Reaction times (ms)	407 [397, 417]	399 [390, 408]	7.6 [1.5, 13.0]^a^
Error rates (%)	1.9 [1.4, 2.4]	2.0 [1.5, 2.5]	−0.01 [−0.73, 0.52]
Latencies of target-elicited saccades (ms)	238 [223, 242]	233 [223, 242]	5.6 [−0.36, 11]
	*Object cue*	*Face cue*	
Latencies of cue-elicited saccades (ms)	218 [207, 230]	202 [191, 213]	16 [2.7, 29]^a^
Number of total saccades	22 [15, 30]	21 [14, 30]	0.35 [−0.81, 1.5]
Number of cue-elicited saccades	2.4 [1.2, 4.2]	2.5 [1.3, 4.3]	−0.13 [−0.44, 0.14]^a^

We assessed FAB with various measures based on task-relevant response and task-irrelevant oculomotor behaviour. In this table, we summarize the predicted values based on the models and indicate whether the difference was credible or not.

a Indicate credible differences.

#### Error rates

Accuracies were high, with a total of 2.61% inaccurate responses across groups (see Table 2). There were no credible differences between the groups or between the conditions (see S5.3).

### Oculomotor behaviour

#### Number of saccades

**Figure 5.**
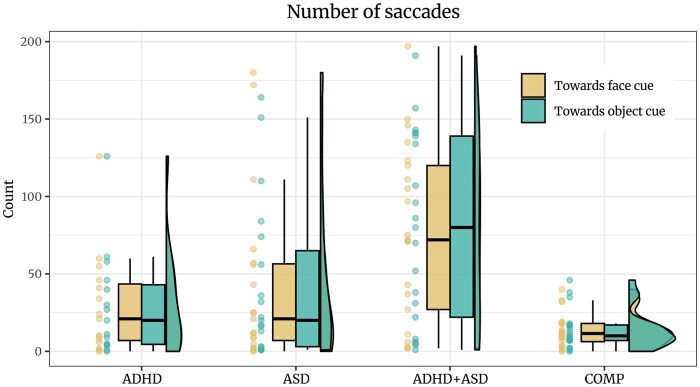
Participants in the ADHD+ASD group produced more saccades compared to the other groups. Saccade number did not depend on the direction of the saccade, that is whether it was produced towards the location of the face or the object cue.

Contrary to our hypothesis, there was no credible difference between number of saccades towards face or object cues in our COMP group (*estimate *= 0.03 [*−*0.11, 0.17], *posterior probability *= 36.33%; see Fig. 5). Exploration of a FAB effect regardless of group similarly indicated no FAB in the form of increased saccade numbers towards the faces over the whole trial (*estimate *= 0.01 [*−*0.05, 0.07], *posterior probability *= 34.08%). On average, participants produced cue-elicited saccades on 4.64% ± 1.06 of the trials, ranging from 0 to 68.75%. Regardless of group, credibly more cue-elicited saccades were produced towards face than object cues (*estimate *=* −*0.14 [*−*0.27, *−*0.02], *posterior probability *= 98.56%; see S11.2). Furthermore, the Bayesian Spearman correlation revealed moderate evidence against an association between the number of saccades towards face cues and FAB (log(*BF*) = *−*1.95, see S10).

#### Saccade latencies

**Figure 6.**
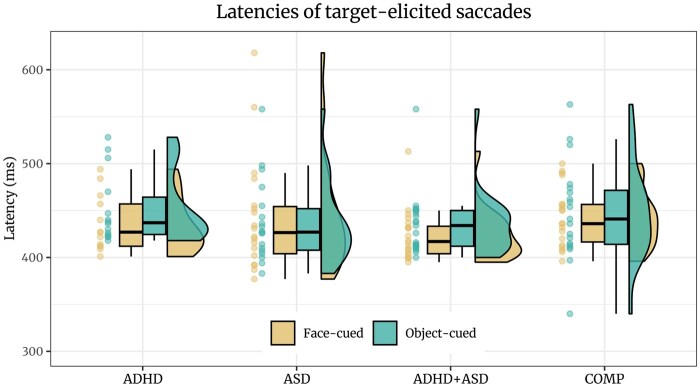
Latencies of saccades elicited by the target presentation show no differences between the groups. Explorative analysis revealed a trend of saccades elicited by targets appearing at the location of the face being produced faster.

Our hypothesis that target-elicited saccades towards face-cued targets have a longer latency in ASD than COMP adults was not confirmed by the data (*estimate *= 0.01 [*−*0.17, 0.18], *posterior probability *= 45.93%; see Fig. 6). Our exploration revealed a trend for faster latencies of both target-elicited saccades in response to face-cued compared to object-cued targets (*estimate *= 0.04 [0, 0.09], *posterior probability *= 97.35%) as well as credibly faster latencies of cue-induced saccades produced towards face than object cues (*estimate *= 0.1 [0.02, 0.18], *posterior probability *= 98.86%; see S12.2). Both effects were independent of group. We also explored dwell times of fixations starting during the time window of the cue-elicited saccades which were only influenced by the target location (see S13).

## Discussion

Social stimuli in general and faces in particular uniquely capture our attention (Palermo and Rhodes 2007). This effect leads to FAB where people are more likely to focus their attention on a face compared to an object (Jakobsen et al. 2021). In the current study, we used a dot-probe paradigm to assess FAB in adults with ADHD and ASD in comparison with adults without psychiatric disorders. While we found a general FAB on reaction times, this bias was neither credibly decreased in autistic adults nor altered in adults with comorbid ASD and ADHD compared to adults without psychiatric diagnoses. However, FAB on reaction times was increased in adults with ADHD compared to adults without psychiatric diagnoses, suggesting that social deficits in ADHD are domain-specific and go beyond general inattentiveness, which is not captured by FAB. These opposite effects in ASD and ADHD speak against a transdiagnostic mechanism affecting social attention in these two neurodevelopmental disorders. Additionally, FAB was observable in some oculomotor behaviour, but these effects did not differ between groups.

We found credible evidence for FAB on both task–relevant responses measured by reaction times and task-irrelevant oculomotor behaviour. Participants produced more cue-elicited saccades towards face compared to object cues. Furthermore, saccades towards a face cue or a face-cued target were produced faster; however, the total number of saccades towards faces compared to objects did not differ. Saccadic bias might be more reliable than measuring FAB with reaction times. In experiments where Pereira et al. (2020), similarly to our study, gave no instructions about eye movements, more cue-elicited saccades were produced towards face than object cues, but they found no differences in mean reaction times. This discrepancy between our and their results could stem from stimulus differences: while our stimuli were matched on luminance, we chose to use naturalistic stimuli showing objects and faces contextually embedded. Pereira et al. (2020) instead opted for stimuli explicitly matched on attractiveness, using grey-scaled, cut-out female faces and houses. In the context of investigating social attention as measured by FAB in clinical groups, we preferred to use a wide array of naturalistic pictures. However, we cannot determine which features of faces led to the observed effect on reaction times in this study. There are several options, including attractiveness, socialness and complexity. Regardless of the driver behind FAB, we conclude that faces receive more exogenous attention than objects in naturalistic scenes measured in both response and oculomotor behaviour.

Our findings suggest that ADHD is associated with an increased attention bias towards faces, adding to the mounting evidence for modified social attention in ADHD described in the introduction. Specifically, we found that while adults with ADHD took longer to react to targets in general, this increase in reaction time was more pronounced for object-cued targets leading to a larger difference in reaction times to face-compared to object-cued targets than adults without any psychiatric diagnoses. However, since all trials included both an object and a face cue, we can only interpret influences on responses based on their interaction and not how reactions to faces or objects in general differ between groups. Frick et al. (2023) found that children with ADHD take longer to disengage from the eye region of faces and that increased symptoms of inattention predict longer latencies of saccades when cued to look away from the eyes. This finding together with our results of increased FAB indicates increased attention towards faces or specific landmarks of faces in people with ADHD. The increase in FAB was not paralleled by altered oculomotor behaviour, indicating that covert attention shifts drive this decrease in reaction times in response to faces. In our study, FAB was unrelated to task performance: more attention to faces might increase reaction times to object-cued targets but similarly decrease reaction times to face-cued targets; thus, our finding of increased FAB is not merely a reflection of general distractibility in ADHD (Aboitiz et al. 2014, World Health Organization 2019), but a specific attention bias to faces due to increased salience. This pattern suggests that social deficits in ADHD are not merely a consequence of domain-general attention deficits but could be their own trait rooted in modified social attention. However, distractibility in ADHD could be caused by modified salience of certain inputs, like our results show for faces. Placing faces in more complex contexts could assess the influence of competing stimuli, e.g. when participants need to filter irrelevant from relevant social cues. If this shift occurs for faces regardless of the focus of current attention, the outcome could be increased distractibility for faces in social situations specifically. Imbalanced social attention could explain issues associated with ADHD regarding social interactions (Gvirts Problovski et al. 2021, Figueiredo et al. 2022) and, subsequently, relationship building (Bagwell et al. 2001, Hoza et al. 2005, Harpin et al. 2016). These symptoms are especially relevant due to their importance for life satisfaction (Gudjonsson et al. 2009); thus, our findings urge researchers to consider not only possible decreases or deficits but also imbalances and even increases when studying social attention and functioning in ADHD.

Our data did not show credible support for our hypothesis regarding decreased FAB in autistic adults, nor for any differences in oculomotor behaviour. Previous literature reported a decreased FAB based on reaction times in ASD in similar dot-probe paradigms (Moore et al. 2012, Kanat et al. 2017), notwithstanding the same sample as in Moore et al. (2012) did show an intact attention bias for faces in a face-in-the-crowd paradigm (Moore et al. 2016). Nonetheless, while a decrease in FAB in ASD compared to the comparison group was not credible in the current sample, neither was a FAB effect credible within the ASD or the comorbid ADHD+ASD group, meaning that these groups did not react credibly faster to face- than object-cued targets. Contrastingly, reactions to face-cued targets were credibly faster than to object-cued targets in both the comparison and the ADHD-only group. Thus, our sample may have been too underpowered to capture decreased FAB when comparing ASD participants to comparison participants’ despite of the lack of a FAB effect in the ASD group. Power was possibly affected by a more heterogeneous sample. Recent years have seen an increase in ASD diagnoses paralleled by a decrease in effect sizes capturing differences between autistic and non-autistic samples (Bachmann et al. 2018, Rødgaard et al. 2019). It has been argued that the decreasing differentiability might be due to an increase in broadening diagnostic inclusion resulting in increasing heterogeneity in samples (Rødgaard et al. 2019). This possibility is strengthened by studies revealing low retention rates for ASD diagnoses when consensus- and guideline-diagnostics are being applied, which might indicate misdiagnoses (Bachmann et al. 2018, Duvall et al. 2025). Furthermore, a Swedish study found a decrease in average autism symptom scores between 2004 and 2014 for children diagnosed between the age of seven and twelve (Arvidsson et al. 2018). Thus, before concluding that FAB is not affected by ASD, for which our analysis did not find credible evidence either, future research will need to incorporate more stringent protocols of diagnostic verification when sampling.

Based on this study, we can only speculate as to the cause of increased FAB in ADHD and possibly decreased FAB in ASD. However, the opposite pattern observed in ASD and ADHD does not support a transdiagnostic mechanism affecting social attention across neurodevelopmental disorders. Instead, changes in the dopaminergic system associated with ADHD (Tripp and Wickens 2009, Chen et al. 2024) might be responsible for an exaggerated salience of faces in ADHD, since dopamine was linked to the processing of perceived salience on the neuronal level (Chen and Bruchas 2021, Kutlu et al. 2021, Fleury et al. 2024). An explorative analysis of FAB in adults with ADHD in our sample who were and who were not on dopamine-affecting medication known to influence attention (Mueller et al. 2017) did not show any differences, although we did not control for timing of medication. The null effect of dopamine-affecting medication on FAB supports a domain-specific social attention modification, with our data suggesting that catecholaminergic imbalances alone are not sufficient to explain social attention modifications in ADHD. Rather, the increased FAB in ADHD could be caused by a modulatory influence of oxytocin which has been proposed in the social salience hypothesis (Shamay-Tsoory and Abu-Akel 2016). While there are some studies suggesting oxytocin modulations in ADHD, their exact nature of these modulations and how they are affected by ADHD medication are still unclear (Petersson and Uvnäs-Moberg 2024). Furthermore, the decreased FAB in ASD could be either explained by specific differences in the modulations of the catecholaminergic system in ASD compared to ADHD or by an ASD-specific modulation of this system by oxytocin. Thus, influences on oxytocin levels, e.g. participant-specific influences like sex, age, and menstrual cycle (Carter 2007, de Vries 2008, Engel et al. 2019, Audunsdottir and Quintana 2022) but possibly also context-dependent influences like time of testing (Perlow et al. 1982) could influence FAB. Determining these influences could establish the exact roles and interplay of oxytocin and dopamine in the increased exogeneous selective attention towards faces.

Importantly, our results do not contradict the wealth of research showing decreased attention as measured in dwell times to faces or eyes in autism (Setien-Ramos et al. 2023): we focused on exogenous selective attention driven by the salience of faces. While this was not credibly attenuated in our study, social attention to faces can still be affected in autism due to other factors of attention, e.g. endogenous attention. Furthermore, exogenous attention could still be attenuated for specific facial landmarks, especially the eye region. Last, dwell times do not necessarily relate to the same attentional capture as FAB in the current study, but rather how long people linger with a stimulus once it has captured their attention.

Comorbid ADHD and ASD has been shown to exacerbate some but not all symptoms of the conditions (Yerys et al. 2009, Harkins et al. 2022), although the interplay between ASD and ADHD symptoms remains overall not sufficiently understood. In a free viewing paradigm, Ioannou et al. (2020) found decreased dwell times on faces for children with both ASD and ADHD as well as decreased dwell times on faces for children with ASD only if the depicted social scenes were more complex. Children with ADHD did not differ from children without psychiatric diagnoses, indicating a potential additive effect of a comorbid diagnosis. Contrary to this pattern, FAB in adults with both ASD and ADHD did not differ credibly from comparison participants in the current study, similarly to the autistic adults without ADHD. Only adults with ADHD showed an increase, suggesting that increased FAB observed in adults with ADHD could be levelled out by ASD. While the sample of adults with ADHD and ASD was exploratory which should be considered when interpreting the current findings, the results of our exploration further cement the importance of considering comorbid diagnosis of ASD and ADHD in research regarding social attention and social cognition. The difference in FAB between our ADHD and our ADHD+ASD group shows that some symptoms may be diminished in the comorbid group, potentially leading to an underestimation of effects if comorbidity is not considered.

Certain constraints of generality apply to the findings presented in this article (Simons et al. 2017). Our exclusion criteria included being over 45 years of age, being non-verbal or having an intellectual disability. Therefore, our sample was limited to a specific subsample of the general population, specifically for autism where an estimated third has an intellectual disability (Zeidan et al. 2022). Additionally, while clinical practice now acknowledges the comorbid diagnosis ASD and ADHD, it was not supported by the ICD-10 which is still predominantly used in Germany (World Health Organization 2004). Thus, some of our ASD and ADHD sample who developed symptoms of one neurodevelopmental disorder earlier than of the other may not have been assessed for the comorbid diagnosis. Unfortunately, the self-reported symptom questionnaires included in this study are not valid as diagnostic instruments and cannot be used to indicate ASD or ADHD diagnoses without further diagnostic assessment (Hines et al. 2012, Jones et al. 2021, Rausch et al. 2024). Future studies should consider verifying not only existing diagnoses but also collaborate with an independent party for a clinical assessment of all clinical participants for the comorbid diagnosis. Furthermore, we did not instruct specific oculomotor behaviour. While the task design encouraged fixating on the fixation cross, participants were allowed to move their eyes freely which explains the large variance in the number of saccades between participants. Last, research has demonstrated that FAB as captured by dot-probe paradigms can be influenced by task design, e.g. cue presentation duration (Jakobsen et al. 2021) or attractiveness of the cues (Pereira et al. 2022) possibly limiting the generalizability of our findings.

This study revealed a robust FAB in response and some oculomotor behaviour, highlighting the inherent salience of faces. Different attentional profiles of adults with ADHD and adults with ASD or comorbid ASD and ADHD highlight the critical need to understand the nuanced and distinct mechanisms behind altered social attention in neurodevelopmental disorders, specifically examining shared and unique mechanisms in ASD and ADHD. Crucially, adults with ADHD exhibited heightened attentional capture by faces compared to objects, indicating increased salience of faces in ADHD and suggesting domain-specific social attention modifications in ADHD.

## Supplementary Material

nsaf112_Supplementary_Data

## Data Availability

Anonymized, raw reaction time data as well preprocessed, anonymized eye-tracking data can be found on GitHub (Plank, 2023/2025). Raw eye-tracking data will be shared upon reasonable request.
