# Supplementary material for: Comparison of face attention bias in adults with ASD, ADHD, or comorbid ADHD+ASD
Source: Soc Cogn Affect Neurosci. 2025 Oct 27;20(1):nsaf112. doi: 10.1093/scan/nsaf112 (PMC12706865; doi:10.1093/scan/nsaf112)

Supplementary materials

Comparison of face attention bias in adults with ASD, ADHD or comorbid ADHD+ASD

I. S. Plank

2025-09-26

Table of Contents

# 1 Introduction

This R Markdown script analyses behavioural data from the FAB (face attention bias) paradigm of the EMBA project. The data was preprocessed before being read into this script.

The task is modeled after Jakobsen et al. (2021), *Attention, Perception, & Psychophysics* and the authors were kind enough to share their stimuli. Each trial starts with a black fixation cross on a white background. Then, a cue consisting of a pair of pictures, one object and one face, is shown with one picture on the left and one on the right of the previous location of the fixation cross. In line with Moore et al. (2012), *J Autism Dev Disord*, we set the duration of the cue presentation to 200ms. Afterwards, a target square appears either at the previous location of the face or the object. Subjects task is to determine the location (right or left) of the target as fast and accurate as possible. The target only disappears when the participant gives their answer.

The visual angle of the target was 1.17 degrees, the visual angle of the cues was 4.25 and the distance of the centre of the target and cue from the fixation cross was 2.67 degrees.

## 1.1 Some general settings

# number of simulations
nsim = 250

# set number of iterations and warmup for models
iter = 3000
warm = 1000

# set the seed
set.seed(2468)

## 1.2 Package versions

The following packages are used in this RMarkdown file:

## [1] "R version 4.5.1 (2025-06-13)"

## [1] "knitr version 1.50"
## [1] "ggplot2 version 3.5.2"
## [1] "brms version 2.22.0"
## [1] "designr version 0.1.13"
## [1] "bridgesampling version 1.1.2"
## [1] "tidyverse version 2.0.0"
## [1] "ggpubr version 0.6.1"
## [1] "vtable version 1.4.8"
## [1] "ggrain version 0.0.4"
## [1] "bayesplot version 1.13.0"
## [1] "SBC version 0.3.0.9000"
## [1] "rstatix version 0.7.2"
## [1] "flextable version 0.9.9"
## [1] "officer version 0.6.10"
## [1] "BayesFactor version 0.9.12.4.7"
## [1] "showtext version 0.9.7"
## [1] "effectsize version 1.0.1"
## [1] "bayestestR version 0.17.0"

## 1.3 General info

We planned to determine the group-level effect subjects following Barr (2013). For each model, experiment specific priors were set based on previous literature or the task (see comments in the code).

We performed prior predictive checks as proposed in Schad, Betancourt and Vasishth (2020) using the SBC package based on the original design with three groups. To do so, we create 250 simulated datasets where parameters are simulated from the priors. These parameters are used to create one fake dataset. Both the true underlying parameters and the simulated discrimination values are saved.

Then, we create graphs showing the prior predictive distribution of the simulated discrimination threshold to check whether our priors fit our general expectations about the data. Next, we perform checks of computational faithfulness and model sensitivity as proposed by Schad, Betancourt and Vasishth (2020) and implemented in the SBC package. We create models for each of the simulated datasets. Last, we calculate performance metrics for each of these models, focusing on the population-level parameters. We did not rerun SBC after adding the exploratory sample of ADHD+ASD.

We base our assessment of the hypothesis on the posterior distributions. Therefore, we perform posterior prdictive checks and in some cases simplify the model by aggregating values to improve posterior fit.

## 1.4 Preparation and group comparisons

First, we load the data and combine it with demographic information including the diagnostic status of the subjects. Then, all predictors are set to sum contrasts. We have a look at the demographics describing our four diagnostic groups: adults with ADHD, autistic adults, autistic adults with ADHD (explorative) and adults without any neurological and psychiatric diagnoses.

Since this is sensitive data, we load the anonymised version of the processed data at this point but also leave the code we used to create it.

# check if the data file exists, if yes load it:
if (!file.exists("FAB_data.RData")) {

 # get demo info for subjects
 df.sub = read_csv(file.path("/home/emba/Documents/EMBA/CentraXX", "EMBA_centraXX.csv"),
 show_col_types = F) %>%
 mutate(
 diagnosis = recode(diagnosis, "CTR" = "COMP"),
 adhd.meds.desc = adhd.meds,
 adhd.meds = if_else(is.na(adhd.meds), FALSE, TRUE)
 )

 # set the data path
 dt.path = "/home/emba/Documents/EMBA/BVET"
 dt.explo = "/home/emba/Documents/EMBA/BVET-explo"

 # load excluded participants (low accuracy, change in diagnosis)
 exc = c(scan(file.path(dt.path, 'FAB_exc.txt'), what="character", sep=NULL),
 scan(file.path(dt.explo, 'FAB_exc.txt'), what="character", sep=NULL))
 df.exc = df.sub %>% filter(subID %in% exc) %>%
 select(diagnosis) %>%
 group_by(diagnosis) %>% count()

 # load the behavioral data and merge with group
 df.fab = merge(df.sub %>% select(subID, diagnosis,
 RAADS_total, ASRS_total, adhd.meds, gender),
 readRDS(file = paste0(dt.path, '/df_FAB.RDS'))) %>%
 mutate_if(is.character, as.factor) %>%
 filter(!(subID %in% exc))
 df.exp = merge(df.sub %>% select(subID, diagnosis,
 RAADS_total, ASRS_total, adhd.meds, gender),
 readRDS(file = paste0(dt.explo, '/df_FAB.RDS'))) %>%
 mutate_if(is.character, as.factor) %>%
 filter(!(subID %in% exc))

 # only keep participants included in the study in the subject data frame
 subIDs = as.character(c(unique(df.fab$subID), unique(df.exp$subID)))
 df.sub = df.sub %>% filter(subID %in% subIDs)

 df.med = df.sub %>% group_by(diagnosis) %>%
 summarise(
 adhd.meds = mean(adhd.meds)
 )

 adhd.meds.desc = unique(df.sub[!is.na(df.sub$adhd.meds.desc),]$adhd.meds.desc)

 # load the eye tracking data and only keep participants included in the study,
 # so no people with more than 33% mistakes, no people without any saccades
 # and no people with too many blinks
 df.sac = rbind(readRDS(file.path(dt.explo, "FAB_ET_data.rds")),
 readRDS(file.path(dt.path, "FAB_ET_data.rds"))) %>%
 merge(., df.sub %>% select(subID, diagnosis), keep.y = T)

 # check groups of people who had no relevant saccades at all
 df.nosac = df.sac %>% filter(is.na(trl)) %>%
 group_by(diagnosis) %>%
 count()

 # anonymise the data
 df.fab = df.fab %>%
 mutate(
 PID = subID,
 subID = as.numeric(subID)
 )
 df.exp = df.exp %>%
 mutate(
 PID = subID,
 subID = as.factor(as.numeric(subID) + max(df.fab$subID))
 )

 # get a correspondence of original PIDs and anonymised subIDs
 df.recode = rbind(df.fab %>% select(PID, subID) %>% distinct(),
 df.exp %>% select(PID, subID) %>% distinct())
 recode = as.character(df.recode$subID)
 names(recode) = df.recode$PID
 df.fab = df.fab %>% select(-PID)
 df.exp = df.exp %>% select(-PID)

 # anonymise ET data in the same way
 df.sac$subID = str_replace_all(df.sac$subID, recode)

 # print gender frequencies and compare them across groups
 tb.gen = xtabs(~ gender + diagnosis, data = df.sub)
 ct.full = contingencyTableBF(tb.gen,
 sampleType = "indepMulti",
 fixedMargin = "cols")
 # since only DAN in the ADHD group, we try again after excluding them
 ct.mf = contingencyTableBF(tb.gen[2:3,],
 sampleType = "indepMulti",
 fixedMargin = "cols")
 # we add this information to our demographics tablerbind(df.demo,
 df.demo = data.frame(
 measurement = "Gender",
 ADHD = sprintf("%.0f - %.0f - %.0f",
 tb.gen["fem","ADHD"],
 tb.gen["mal","ADHD"],
 tb.gen["dan","ADHD"]
 ),
 `ADHD+ASD` = sprintf("%.0f - %.0f - %.0f",
 tb.gen["fem","BOTH"],
 tb.gen["mal","BOTH"],
 tb.gen["dan","BOTH"]
 ),
 ASD = sprintf("%.0f - %.0f - %.0f",
 tb.gen["fem","ASD"],
 tb.gen["mal","ASD"],
 tb.gen["dan","ASD"]
 ),
 COMP = sprintf("%.0f - %.0f - %.0f",
 tb.gen["fem","COMP"],
 tb.gen["mal","COMP"],
 tb.gen["dan","COMP"]
 ),
 bf.log = ct.full@bayesFactor[["bf"]]
 )

 # then, we save some more gender information in a table in case we need it
 tb.gen = xtabs(~ gender + diagnosis + cis, data = df.sub)

 # get the gender descriptions of the not-male and not-female participants
 gen.desc = unique(tolower(df.sub[df.sub$gender == "dan",]$gender_desc))

 # convert the measures to long which we include in the participant table
 df.sublng = df.sub %>%
 # rename some of the variables
 rename(
 "RADS-R" = "RAADS_total",
 "ASRS-v1.1" = "ASRS_total",
 "Age" = "age",
 "IQ estimate" = "iq",
 "Education" = "edu"
 ) %>%
 select(diagnosis, Age, `IQ estimate`, `ASRS-v1.1`, `RADS-R`, Education) %>%
 pivot_longer(cols = where(is.numeric)) %>%
 mutate_if(is.character, as.factor)

 # initialise the data frame for posthoc tests
 df.post = data.frame()

 # now we loop through our measurements to create our demographics table
 for (m in unique(df.sublng$name)) {
 # select the relevant part of df.sub
 df.rel = df.sublng %>% filter(name == m)
 # check which of the group's data is not normally distributed
 df.sht = df.rel %>%
 group_by(diagnosis) %>%
 shapiro_test(value) %>%
 filter(p < 0.05)
 # if more than zero is not normally distributed...
 if (nrow(df.sht) > 0) {
 # rank transform the data
 df.rel = df.rel %>% ungroup() %>% mutate(value = rank(value))
 }
 # compute the ANOVA
 aov = anovaBF(value ~ diagnosis, data = df.rel)
 # get back the original, untransformed values
 df.rel = df.sublng %>% filter(name == m)
 # put all the information into the demographics table
 df.demo = rbind(df.demo,
 data.frame(
 measurement = m,
 ADHD = sprintf("%.2f ±%.2f (%.0f to %.0f)",
 # ignore NAs because edu missing for one person
 mean(df.rel[df.rel$diagnosis == "ADHD",]$value, na.rm = T),
 sd(df.rel[df.rel$diagnosis == "ADHD",]$value, na.rm = T)/
 sqrt(sum(df.rel$diagnosis == "ADHD")),
 min(df.rel[df.rel$diagnosis == "ADHD",]$value, na.rm = T),
 max(df.rel[df.rel$diagnosis == "ADHD",]$value, na.rm = T)
 ),
 `ADHD+ASD` = sprintf("%.2f ±%.2f (%.0f to %.0f)",
 mean(df.rel[df.rel$diagnosis == "BOTH",]$value),
 sd(df.rel[df.rel$diagnosis == "BOTH",]$value)/
 sqrt(sum(df.rel$diagnosis == "BOTH")),
 min(df.rel[df.rel$diagnosis == "BOTH",]$value),
 max(df.rel[df.rel$diagnosis == "BOTH",]$value)
 ),
 ASD = sprintf("%.2f ±%.2f (%.0f to %.0f)",
 mean(df.rel[df.rel$diagnosis == "ASD",]$value),
 sd(df.rel[df.rel$diagnosis == "ASD",]$value)/
 sqrt(sum(df.rel$diagnosis == "ASD")),
 min(df.rel[df.rel$diagnosis == "ASD",]$value),
 max(df.rel[df.rel$diagnosis == "ASD",]$value)
 ),
 COMP = sprintf("%.2f ±%.2f (%.0f to %.0f)",
 mean(df.rel[df.rel$diagnosis == "COMP",]$value),
 sd(df.rel[df.rel$diagnosis == "COMP",]$value)/
 sqrt(sum(df.rel$diagnosis == "COMP")),
 min(df.rel[df.rel$diagnosis == "COMP",]$value),
 max(df.rel[df.rel$diagnosis == "COMP",]$value)
 ),
 bf.log = aov@bayesFactor[["bf"]]
 ))

 # next, we want to check whether there are group differences
 if (abs(exp(aov@bayesFactor$bf)) > 3) {
 # do the group comparisons
 aov.ADHDvASD = anovaBF(value ~ diagnosis,
 data = df.rel %>% filter(diagnosis %in% c("ADHD", "ASD")))
 aov.ADHDvBOTH = anovaBF(value ~ diagnosis,
 data = df.rel %>% filter(diagnosis %in% c("ADHD", "BOTH")))
 aov.ADHDvCOMP = anovaBF(value ~ diagnosis,
 data = df.rel %>% filter(diagnosis %in% c("ADHD", "COMP")))
 aov.ASDvBOTH = anovaBF(value ~ diagnosis,
 data = df.rel %>% filter(diagnosis %in% c("ASD", "BOTH")))
 aov.ASDvCOMP = anovaBF(value ~ diagnosis,
 data = df.rel %>% filter(diagnosis %in% c("ASD", "COMP")))
 aov.BOTHvCOMP = anovaBF(value ~ diagnosis,
 data = df.rel %>% filter(diagnosis %in% c("BOTH", "COMP")))
 # put into the posthoc data frame
 df.post = rbind(df.post,
 data.frame(
 measurement = m,
 ADHDvASD = aov.ADHDvASD@bayesFactor[["bf"]],
 ADHDvBOTH = aov.ADHDvBOTH@bayesFactor[["bf"]],
 ADHDvCOMP = aov.ADHDvCOMP@bayesFactor[["bf"]],
 ASDvBOTH = aov.ASDvBOTH@bayesFactor[["bf"]],
 ASDvCOMP = aov.ASDvCOMP@bayesFactor[["bf"]],
 BOTHvCOMP = aov.BOTHvCOMP@bayesFactor[["bf"]]
 ))
 }
 }

 # save the demographics and the posthoc table as word documents
 read_docx() %>%
 body_add_table(df.demo %>% arrange(measurement) %>%
 mutate(bf.log =
 if_else(
 bf.log > log(3),
 sprintf("%.3f*", bf.log),
 sprintf("%.3f", bf.log)))) %>%
 print(target = "FAB_demo.docx")
 read_docx() %>%
 body_add_table(df.post %>%
 mutate_if(is.numeric,
 ~ifelse(.>log(3),sprintf("%.3f*", .),sprintf("%.3f", .)))) %>%
 print(target = "FAB_post.docx")

 # check how many of each group are above threshold for asrs and rads
 tb.screen = xtabs(~ diagnosis + screening,
 data = df.sub %>%
 select(diagnosis, ASRS_screen, RAADS_total) %>%
 mutate(
 screening = case_when(
 ASRS_screen >= 4 & RAADS_total > 81 ~ "screenBOTH",
 ASRS_screen >= 4 & RAADS_total <= 81 ~ "screenADHD",
 ASRS_screen < 4 & RAADS_total <= 81 ~ "screenNone",
 ASRS_screen < 4 & RAADS_total > 81 ~ "screenASD"
 )
 ))

 # save it all
 save(df.fab, df.sac, df.exp, ct.full, ct.mf, df.exc, tb.screen,
 df.nosac, gen.desc, tb.gen, adhd.meds.desc, df.med,
 file = "FAB_data.RData")

} else {

 load("FAB_data.RData")

}

# print the group of excluded participants based on low accuracy (< 2/3)
# as well as change in diagnosis
kable(df.exc)

| diagnosis | n |
| --- | --- |
| ADHD | 1 |
| ASD | 2 |
| BOTH | 1 |

rm(df.exc)

# print the group of the participants included in behavioural and eye tracking
kable(merge(
 df.sac %>% filter(!is.na(trl)) %>% select(subID, diagnosis) %>% distinct() %>%
 group_by(diagnosis) %>% summarise(`sample size eye tracking` = n()),
 rbind(df.fab, df.exp) %>% select(subID, diagnosis) %>% distinct() %>%
 group_by(diagnosis) %>% summarise(`sample size behavioural` = n())
 ))

| diagnosis | sample size eye tracking | sample size behavioural |
| --- | --- | --- |
| ADHD | 16 | 23 |
| ASD | 19 | 23 |
| BOTH | 21 | 22 |
| COMP | 21 | 24 |

# Note: eye-tracking only collected if calibration accuracy < 0.5, then exclusion:
# 1 due to more than 1/3 blinks
# 2 due to no relevant saccades
# how many have been removed due to no relevant saccades?
kable(df.nosac)

| diagnosis | n |
| --- | --- |
| ASD | 1 |
| COMP | 1 |

rm(df.nosac)

# print the how many people are above threshold for clinical self assessment
kable(tb.screen)

|  | screenADHD | screenASD | screenBOTH | screenNone |
| --- | --- | --- | --- | --- |
| ADHD | 4 | 2 | 11 | 6 |
| ASD | 1 | 16 | 5 | 1 |
| BOTH | 0 | 8 | 14 | 0 |
| COMP | 1 | 3 | 0 | 20 |

# print the outcome of the two contingency tables for comparison
# based on full sample with agender, diverse and non-binary in one category
ct.full@bayesFactor

## bf error time code
## Non-indep. (a=1) -4.020544 0 Mon Jul 14 12:30:52 2025 30c72a3236db

# based on female and male participants only
ct.mf@bayesFactor

## bf error time code
## Non-indep. (a=1) -2.43588 0 Mon Jul 14 12:30:52 2025 30c721d9416c5

# combine explorative and original data
df.fab = rbind(df.fab, df.exp)

# set the levels of the diagnosis factor
df.fab$diagnosis = factor(df.fab$diagnosis,
 levels = c("ADHD", "ASD", "BOTH", "COMP"))

# set and print the contrasts
contrasts(df.fab$cue) = contr.sum(2)
contrasts(df.fab$cue)

## [,1]
## face 1
## object -1

contrasts(df.fab$diagnosis) = contr.sum(4)
contrasts(df.fab$diagnosis)

## [,1] [,2] [,3]
## ADHD 1 0 0
## ASD 0 1 0
## BOTH 0 0 1
## COMP -1 -1 -1

The three diagnostic groups are similar in age, IQ and gender distribution. However, they seem to differ in their questionnaire scores measuring ADHD (ASRS), depression (BDI), autism (RAADS) and alexithymia (TAS).

# 2 Reaction times

First, we analyse the reaction times for all correctly answered trials to assess whether participants answer faster if the target appears at the previous location of the face, which we refer to as face attention bias (FAB). In our preregistration, we formulated the following hypotheses:

H1a) COMP participants react faster in response to targets appearing on the side of the face compared to targets appearing on the side of the object (face attention bias; Jakobsen et al., 2021). H1b) ADHD participants react slower than COMP participants in both cue conditions (Sonuga-Barke et al., 2004). H1c) ASD participants react slower than COMP participants in both cue conditions (Ghosn et al., 2018). H1d) Face attention bias is decreased in ASD participants compared to COMP participants (Moore et al., 2012). H1e) Face attention bias in ADHD participants differs from face attention bias in COMP participants.

## 2.1 Full model

### 2.1.1 Simulation-based calibration

First, we attempted to use a full model for the data. This model includes multiple instances of each stimulus per participant in each of the conditions (face cue or object cue). Therefore, we need slopes for the cue per subject as well as for cue, diagnosis and their interaction for the stimulus.

code = "FAB"

# full model formula
f.fab = brms::bf(rt.cor ~ diagnosis * cue + (cue | subID) + (cue * diagnosis | stm) )

# set informed priors based on previous results
priors = c(
 # general priors based on SBV
 prior(normal(6, 0.3), class = Intercept),
 prior(normal(0, 0.5), class = sigma),
 prior(normal(0, 0.1), class = sd),
 prior(lkj(2), class = cor),
 # face attention bias effect based on Jakobsen et al. (2021)
 prior(normal(-0.01, 0.04), class = b, coef = cue1),
 # ADHD subjects being slower based on Pievsky & McGrath (2018)
 prior(normal(0.025, 0.04), class = b, coef = diagnosis1),
 # ASD subjects being slower based on Morrison et al. (2018)
 prior(normal(0.025, 0.04), class = b, coef = diagnosis2),
 # decreased FAB in ASD subjects based on Moore et al. (2012)
 prior(normal(0.01, 0.04), class = b, coef = diagnosis2:cue1),
 # no specific expectations for FAB in ADHD
 prior(normal(0, 0.04), class = b),
 # shift
 prior(normal(200, 100), class = ndt)
)

# check if the SBC already exists
if (file.exists(file.path(cache_dir, sprintf("df_res_%s.rds", code)))) {
 # load in the results of the SBC
 df.results = readRDS(file.path(cache_dir, sprintf("df_res_%s.rds", code)))
 df.backend = readRDS(file.path(cache_dir, sprintf("df_div_%s.rds", code)))
 dat = readRDS(file.path(cache_dir, sprintf("dat_%s.rds", code)))
} else {
 # perform the SBC
 gen = SBC_generator_brms(f.fab, data = df.fab, prior = priors,
 family = shifted_lognormal,
 thin = 50, warmup = 20000, refresh = 2000)
 bck = SBC_backend_brms_from_generator(gen, chains = 4, thin = 1,
 init = 0.1, warmup = warm, iter = iter)
 dat = generate_datasets(gen, nsim)
 saveRDS(dat, file.path(cache_dir, sprintf("dat_%s.rds", code)))
 res = compute_SBC(dat,
 bck,
 cache_mode = "results",
 cache_location = file.path(cache_dir, sprintf("res_%s", code)))
 df.results = res$stats
 df.backend = res$backend_diagnostics
 saveRDS(df.results, file = file.path(cache_dir, paste0("df_res_", code, ".rds")))
 saveRDS(df.backend, file = file.path(cache_dir, paste0("df_div_", code, ".rds")))
}

We start by investigating the rhats and the number of divergent samples. This shows that 6 of 250 simulations had at least one parameter that had an rhat of at least 1.05, and 1 model had divergent samples (mean number of samples of the simulations with divergent samples: 4). This suggests that this model performs well.

Next, we can plot the simulated values to perform prior predictive checks.

# create a matrix out of generated data
dvname = gsub(" ", "", gsub("[\\|~].*", "", f.fab)[1])
dvfakemat = matrix(NA, nrow(dat[['generated']][[1]]), length(dat[['generated']]))
for (i in 1:length(dat[['generated']])) {
 dvfakemat[,i] = dat[['generated']][[i]][[dvname]]
}
truePars = dat$variables

# set large values to a max
dvfakemat[dvfakemat > 2000] = 2000

# compute one histogram per simulated data-set
binwidth = 20
breaks = seq(0, max(dvfakemat, na.rm=T) + binwidth, binwidth)
histmat = matrix(NA, ncol = length(dat), nrow = length(breaks)-1)
for (i in 1:nrow(truePars)) {
 histmat[,i] = hist(dvfakemat[,i], breaks = breaks, plot = F)$counts
}
# for each bin, compute quantiles across histograms
probs = seq(0.1, 0.9, 0.1)
quantmat= as.data.frame(matrix(NA, nrow=dim(histmat)[1], ncol = length(probs)))
names(quantmat) = paste0("p", probs)
for (i in 1:dim(histmat)[1]) {
 quantmat[i,] = quantile(histmat[i,], p = probs)
}
quantmat$x = breaks[2:length(breaks)] - binwidth/2 # add bin mean
p1 = ggplot(data = quantmat, aes(x = x)) +
 geom_ribbon(aes(ymax = p0.9, ymin = p0.1), fill = c_light) +
 geom_ribbon(aes(ymax = p0.8, ymin = p0.2), fill = c_light_highlight) +
 geom_ribbon(aes(ymax = p0.7, ymin = p0.3), fill = c_mid) +
 geom_ribbon(aes(ymax = p0.6, ymin = p0.4), fill = c_mid_highlight) +
 geom_line(aes(y = p0.5), colour = c_dark, linewidth = 1) +
 labs(title = "Distribution of simulated discriminations", y = "", x = "") +
 theme_bw()

tmpM = apply(dvfakemat, 2, mean) # mean
tmpSD = apply(dvfakemat, 2, sd)
p2 = ggplot() +
 stat_bin(aes(x = tmpM), fill = c_dark) +
 labs(x = "Mean RTs (ms)", title = "Means of simulated RTs") +
 theme_bw()
p3 = ggplot() +
 stat_bin(aes(x = tmpSD), fill = c_dark) +
 labs(x = "SD RTs (ms)", title = "SDs of simulated RTs") +
 theme_bw()
p = ggarrange(p1,
 ggarrange(p2, p3, ncol = 2, labels = c("B", "C")),
 nrow = 2, labels = "A")
annotate_figure(p,
 top = text_grob("Prior predictive checks: reaction times",
 face = "bold", size = 14))


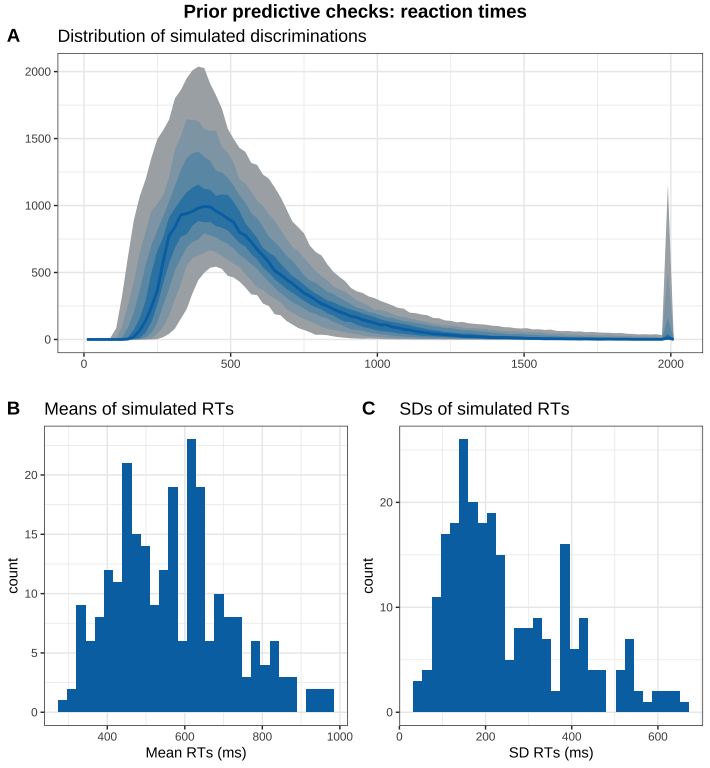


Subfigure A shows the distribution of the simulated data with bluer bands being more likely than greyer bands. It shows a distribution that fits our expectations about reaction times in a simple decision task. The same applies to the distribution of the means and standard deviations in the simulated datasets. We go ahead with these priors and check the results of the SBC. We only plot the results from the models that had no divergence issues.

# get simulation numbers with issues
rank = max(df.results$max_rank)
check = merge(df.results %>%
 group_by(sim_id) %>%
 summarise(
 rhat = max(rhat, na.rm = T),
 mean_rank = mean(max_rank)
 ) %>%
 filter(rhat >= 1.05 | mean_rank != rank),
 df.backend %>% filter(n_divergent > 0), all = T)

# plot SBC with functions from the SBC package focusing on population-level parameters
df.results.b = df.results %>%
 filter(substr(variable, 1, 2) == "b_") %>%
 filter(!(sim_id %in% check$sim_id))
p1 = plot_ecdf_diff(df.results.b) + theme_bw() + theme(legend.position = "none") +
 scale_x_continuous(breaks=scales::pretty_breaks(n = 3)) +
 scale_y_continuous(breaks=scales::pretty_breaks(n = 3))
p2 = plot_rank_hist(df.results.b, bins = 20) + theme_bw() +
 scale_x_continuous(breaks=scales::pretty_breaks(n = 3)) +
 scale_y_continuous(breaks=scales::pretty_breaks(n = 3))
p3 = plot_sim_estimated(df.results.b, alpha = .8) + theme_bw() +
 scale_x_continuous(breaks=scales::pretty_breaks(n = 3)) +
 scale_y_continuous(breaks=scales::pretty_breaks(n = 3))
p4 = plot_contraction(df.results.b,
 prior_sd = setNames(
 c(as.numeric(
 gsub(".*, (.+)\\).*", "\\1",
 priors[priors$class == "Intercept",]$prior)),
 as.numeric(
 gsub(".*, (.+)\\).*", "\\1",
 priors[priors$class == "b",]$prior))),
 unique(df.results.b$variable))) +
 theme_bw() +
 scale_x_continuous(breaks=scales::pretty_breaks(n = 3)) +
 scale_y_continuous(breaks=scales::pretty_breaks(n = 3))

p = ggarrange(p1, p2, p3, p4, labels = "AUTO", ncol = 1, nrow = 4)
annotate_figure(p, top =
 text_grob("Computational faithfulness and model sensitivity",
 face = "bold", size = 14))


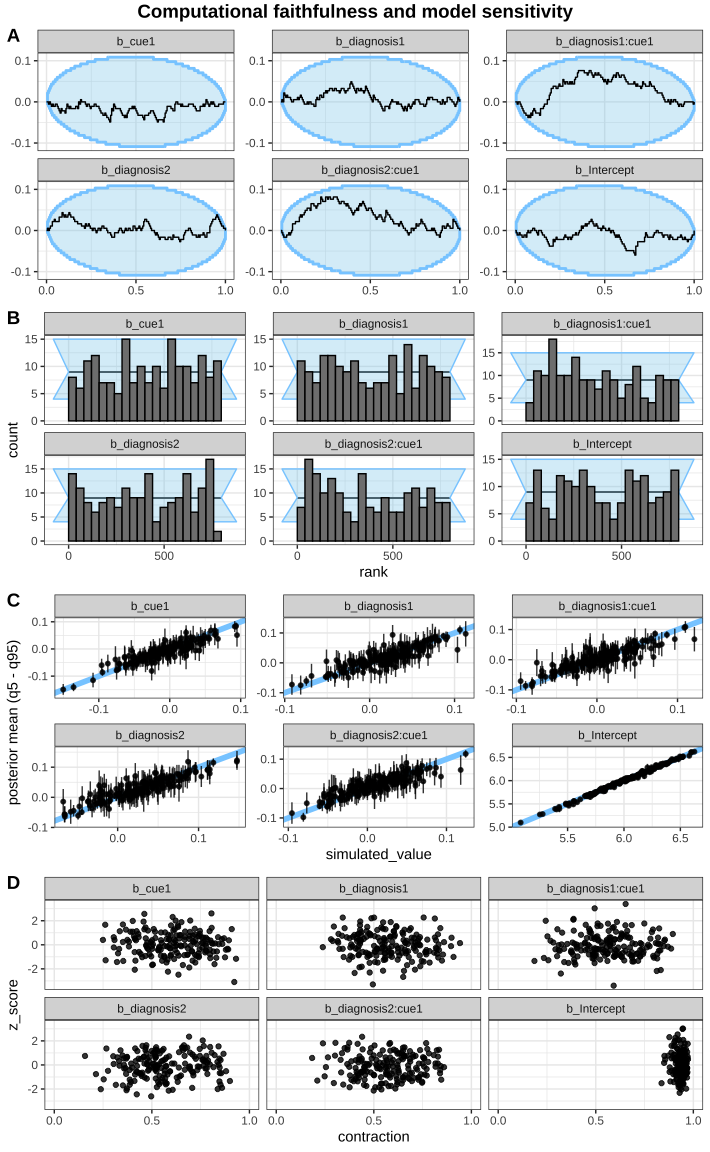


Next, we check the ranks of the parameters. If the model is unbiased, these should be uniformly distributed (Schad, Betancourt and Vasishth, 2020). The sample empirical cumulative distribution function (ECDF) lies within the theoretical distribution (95%) and the rank histogram also shows ranks within the 95% expected range, although there are some small deviations. We judge this to be acceptable.

Then, we investigated the relationship between the simulated true parameters and the posterior estimates. Although there are individual values diverging from the expected pattern, most parameters were recovered successfully within an uncertainty interval of alpha = 0.05.

Last, we explore the z-score and the posterior contraction of our population-level predictors. The z-score “determines the distance of the posterior mean from the true simulating parameter”, while the posterior contraction “estimates how much prior uncertainty is reduced in the posterior estimation” (Schad, Betancourt and Vasisth, 2020). All of this looks good for this model.

### 2.1.2 Posterior predictive checks

As the next step, we fit the model to the data, check whether there are divergence or rhat issues, and then check whether the chains have converged.

# fit the full model
set.seed(2469)
m.fab = brm(f.fab,
 df.fab, prior = priors,
 family = shifted_lognormal,
 iter = iter, warmup = warm,
 backend = "cmdstanr", threads = threading(8),
 file = "m_fab_full"
 )
rstan::check_hmc_diagnostics(m.fab$fit)

##
## Divergences:

## 0 of 8000 iterations ended with a divergence.

##
## Tree depth:

## 0 of 8000 iterations saturated the maximum tree depth of 10.

##
## Energy:

## E-BFMI indicated no pathological behavior.

# check that rhats are below 1.01
sum(brms::rhat(m.fab) >= 1.01, na.rm = T)

## [1] 0

# check the trace plots
post.draws = as_draws_df(m.fab)
mcmc_trace(post.draws, regex_pars = "^b_",
 facet_args = list(ncol = 3)) +
 scale_x_continuous(breaks=scales::pretty_breaks(n = 3)) +
 scale_y_continuous(breaks=scales::pretty_breaks(n = 3))

## Scale for x is already present.
## Adding another scale for x, which will replace the existing scale.


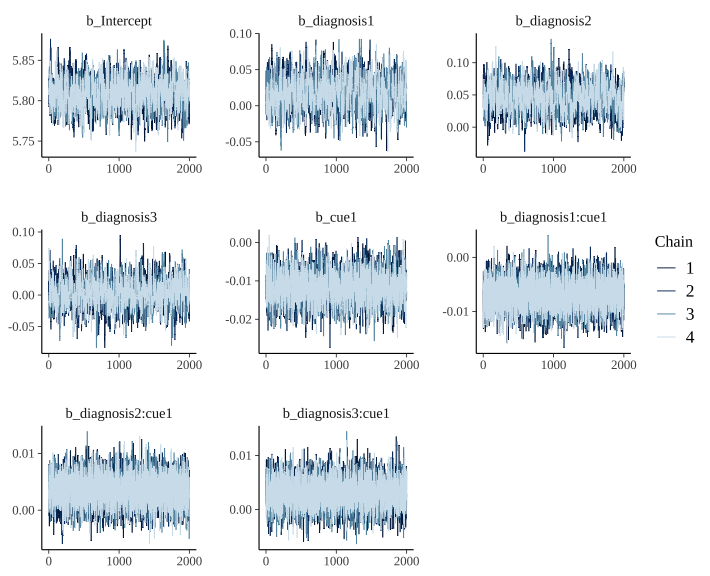


This model has no pathological behaviour with E-BFMI, no divergent samples and no rhat that is higher or equal to 1.01. Therefore, we go ahead and perform our posterior predictive checks.

# get posterior predictions
post.pred = posterior_predict(m.fab, ndraws = nsim)

# check the fit of the predicted data compared to the real data
p1 = pp_check(m.fab, ndraws = nsim) +
 theme_bw() + theme(legend.position = "none") + xlim(0, 2000)

# get rid of NAs in data frame for plotting
df.fab.na = df.fab[!is.na(df.fab$rt.cor),]

# distributions of means compared to the real values per group
p2 = ppc_stat_grouped(df.fab.na$rt.cor, post.pred, df.fab.na$diagnosis) +
 theme_bw() + theme(legend.position = "none")

# distributions of means compared to the real values per cue
p3 = ppc_stat_grouped(df.fab.na$rt.cor, post.pred, df.fab.na$cue) +
 theme_bw() + theme(legend.position = "none")

p = ggarrange(p1, p2, p3,
 nrow = 3, ncol = 1, labels = "AUTO")
annotate_figure(p,
 top = text_grob("Posterior predictive checks: RTs",
 face = "bold", size = 14))


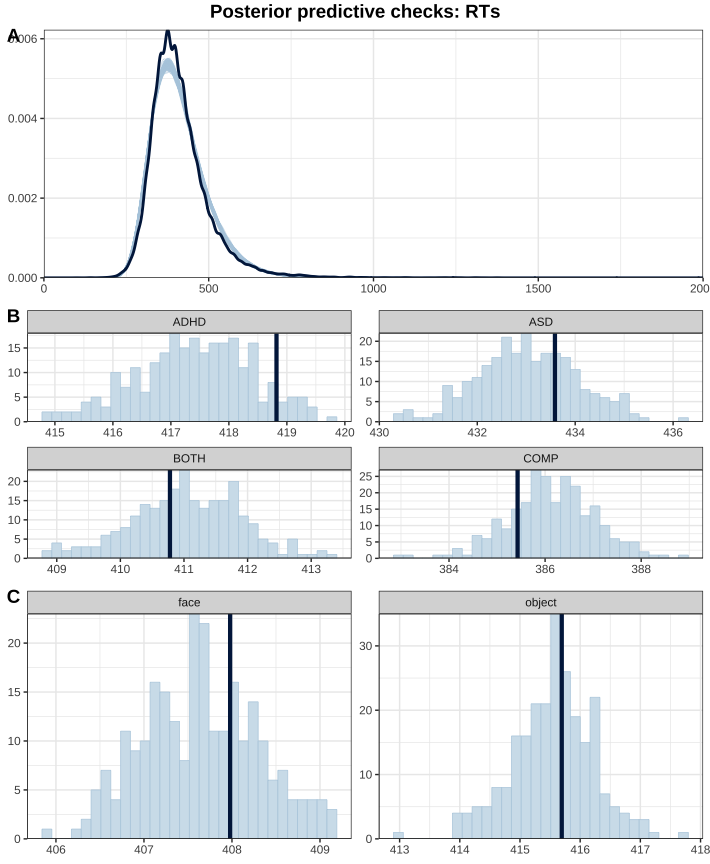


Although the overall shape in subfigure A of the simulated data fits well with the real data, the model seems to underestimate the reaction times of the ADHD and ASD groups and overestimate the reaction times of the COMP group: the dark blue line shows the mean of the actual dataset while the light blue bars show the distribution of the predicted data.

Since we are interested in accurate estimates, we decide to aggregate with the median of the reaction times per stimulus (face-object cue combination) and cue. Then, there are no missing values in the data and we model an estimate for each specific stimulus and cue combination for each participant.

## 2.2 Aggregated model

First, we compute the aggregation and have a quick look at the resulting data.

# keep full dataframe
df.fab.full = df.fab

# aggregate reaction times
df.fab = df.fab %>%
 group_by(subID, diagnosis, stm, cue) %>%
 summarise(
 rt.cor = median(rt.cor, na.rm = T)
 ) %>% ungroup() %>%
 mutate_if(is.character, as.factor)

# set and print the contrasts
contrasts(df.fab$cue) = contr.sum(2)
contrasts(df.fab$cue)

## [,1]
## face 1
## object -1

contrasts(df.fab$diagnosis) = contr.sum(4)
contrasts(df.fab$diagnosis)

## [,1] [,2] [,3]
## ADHD 1 0 0
## ASD 0 1 0
## BOTH 0 0 1
## COMP -1 -1 -1

summary(df.fab)

## subID diagnosis stm cue rt.cor
## 1 : 72 ADHD:1656 1_10 : 184 face :3312 Min. :256.0
## 10 : 72 ASD :1656 1_11 : 184 object:3312 1st Qu.:364.0
## 11 : 72 BOTH:1584 1_12 : 184 Median :395.5
## 12 : 72 COMP:1728 1_7 : 184 Mean :407.4
## 13 : 72 1_8 : 184 3rd Qu.:436.1
## 14 : 72 1_9 : 184 Max. :919.0
## (Other):6192 (Other):5520

There are now no NAs in the data, because no one made an error on all instances of one stimulus combination.

### 2.2.1 Stimulation-based calibration

We again perform an SBC. The model formula and priors can stay the same.

code = "FAB_agg"

# check if the SBC already exists
if (file.exists(file.path(cache_dir, sprintf("df_res_%s.rds", code)))) {
 # load in the results of the SBC
 df.results = readRDS(file.path(cache_dir, sprintf("df_res_%s.rds", code)))
 df.backend = readRDS(file.path(cache_dir, sprintf("df_div_%s.rds", code)))
 dat = readRDS(file.path(cache_dir, sprintf("dat_%s.rds", code)))
} else {
 # perform the SBC
 gen = SBC_generator_brms(f.fab, data = df.fab, prior = priors,
 family = shifted_lognormal,
 thin = 50, warmup = 20000, refresh = 2000)
 bck = SBC_backend_brms_from_generator(gen, chains = 4, thin = 1,
 init = 0.1, warmup = warm, iter = iter)
 set.seed(468)
 if (file.exists(file.path(cache_dir, sprintf("dat_%s.rds", code)))) {
 dat = readRDS(file.path(cache_dir, sprintf("dat_%s.rds", code)))
 } else {
 dat = generate_datasets(gen, nsim)
 saveRDS(dat, file.path(cache_dir, sprintf("dat_%s.rds", code)))
 }
 res = compute_SBC(dat,
 bck,
 cache_mode = "results",
 cache_location = file.path(cache_dir, sprintf("res_%s", code)))
 df.results = res$stats
 df.backend = res$backend_diagnostics
 saveRDS(df.results, file = file.path(cache_dir, paste0("df_res_", code, ".rds")))
 saveRDS(df.backend, file = file.path(cache_dir, paste0("df_div_", code, ".rds")))
}

set.seed(4682)

We start by investigating the rhats and the number of divergent samples. This shows that 3 of 250 simulations had at least one parameter that had an rhat of at least 1.05, and 2 models had divergent samples (mean number of samples of the simulations with divergent samples: 17.5). This suggests that this model performs well enough and only few simulated models exhibit issues.

Next, we can plot the simulated values to perform prior predictive checks.

# create a matrix out of generated data
dvname = gsub(" ", "", gsub("[\\|~].*", "", f.fab)[1])
dvfakemat = matrix(NA, nrow(dat[['generated']][[1]]), length(dat[['generated']]))
for (i in 1:length(dat[['generated']])) {
 dvfakemat[,i] = dat[['generated']][[i]][[dvname]]
}
truePars = dat$variables

# set large values to a max
dvfakemat[dvfakemat > 2000] = 2000

# compute one histogram per simulated data-set
binwidth = 20
breaks = seq(0, max(dvfakemat, na.rm=T) + binwidth, binwidth)
histmat = matrix(NA, ncol = length(dat), nrow = length(breaks)-1)
for (i in 1:nrow(truePars)) {
 histmat[,i] = hist(dvfakemat[,i], breaks = breaks, plot = F)$counts
}
# for each bin, compute quantiles across histograms
probs = seq(0.1, 0.9, 0.1)
quantmat= as.data.frame(matrix(NA, nrow=dim(histmat)[1], ncol = length(probs)))
names(quantmat) = paste0("p", probs)
for (i in 1:dim(histmat)[1]) {
 quantmat[i,] = quantile(histmat[i,], p = probs)
}
quantmat$x = breaks[2:length(breaks)] - binwidth/2 # add bin mean
p1 = ggplot(data = quantmat, aes(x = x)) +
 geom_ribbon(aes(ymax = p0.9, ymin = p0.1), fill = c_light) +
 geom_ribbon(aes(ymax = p0.8, ymin = p0.2), fill = c_light_highlight) +
 geom_ribbon(aes(ymax = p0.7, ymin = p0.3), fill = c_mid) +
 geom_ribbon(aes(ymax = p0.6, ymin = p0.4), fill = c_mid_highlight) +
 geom_line(aes(y = p0.5), colour = c_dark, linewidth = 1) +
 labs(title = "Distribution of simulated discriminations", y = "", x = "") +
 theme_bw()

tmpM = apply(dvfakemat, 2, mean) # mean
tmpSD = apply(dvfakemat, 2, sd)
p2 = ggplot() +
 stat_bin(aes(x = tmpM), fill = c_dark) +
 labs(x = "Mean RTs (ms)", title = "Means of simulated RTs") +
 theme_bw()
p3 = ggplot() +
 stat_bin(aes(x = tmpSD), fill = c_dark) +
 labs(x = "SD RTs (ms)", title = "SDs of simulated RTs") +
 theme_bw()
p = ggarrange(p1,
 ggarrange(p2, p3, ncol = 2, labels = c("B", "C")),
 nrow = 2, labels = "A")
annotate_figure(p,
 top = text_grob("Prior predictive checks: reaction times",
 face = "bold", size = 14))


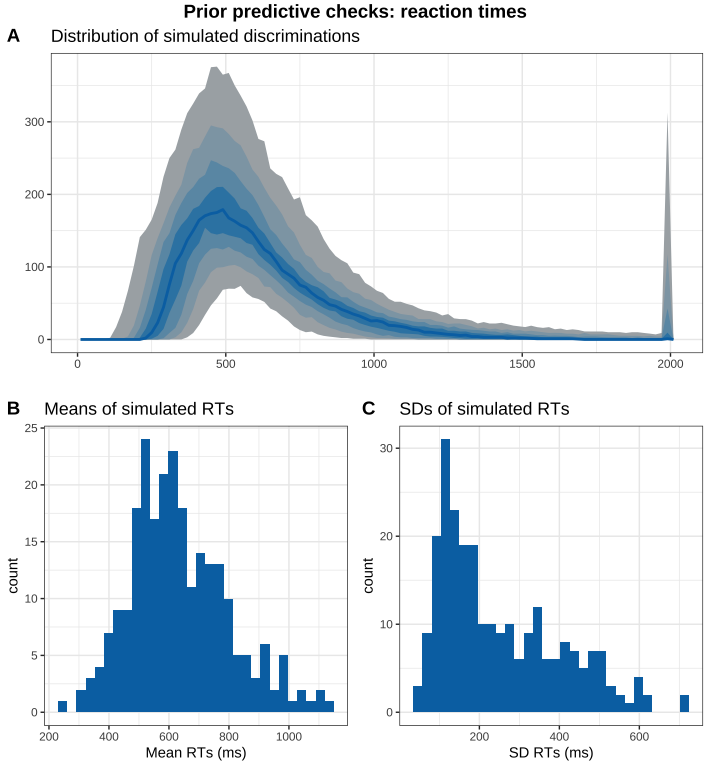


Again, this all looks good.

# get simulation numbers with issues
rank = max(df.results$max_rank)
check = merge(df.results %>%
 group_by(sim_id) %>%
 summarise(
 rhat = max(rhat, na.rm = T),
 mean_rank = mean(max_rank)
 ) %>%
 filter(rhat >= 1.05 | mean_rank != rank),
 df.backend %>% filter(n_divergent > 0), all = T)

# plot SBC with functions from the SBC package focusing on population-level parameters
df.results.b = df.results %>%
 filter(substr(variable, 1, 2) == "b_") %>%
 filter(!(sim_id %in% check$sim_id))
p1 = plot_ecdf_diff(df.results.b) + theme_bw() + theme(legend.position = "none") +
 scale_x_continuous(breaks=scales::pretty_breaks(n = 3)) +
 scale_y_continuous(breaks=scales::pretty_breaks(n = 3))
p2 = plot_rank_hist(df.results.b, bins = 20) + theme_bw() +
 scale_x_continuous(breaks=scales::pretty_breaks(n = 3)) +
 scale_y_continuous(breaks=scales::pretty_breaks(n = 3))
p3 = plot_sim_estimated(df.results.b, alpha = .8) + theme_bw() +
 scale_x_continuous(breaks=scales::pretty_breaks(n = 3)) +
 scale_y_continuous(breaks=scales::pretty_breaks(n = 3))
p4 = plot_contraction(df.results.b,
 prior_sd = setNames(
 c(as.numeric(
 gsub(".*, (.+)\\).*", "\\1",
 priors[priors$class == "Intercept",]$prior)),
 as.numeric(
 gsub(".*, (.+)\\).*", "\\1",
 priors[priors$class == "b",]$prior))),
 unique(df.results.b$variable))) +
 theme_bw() +
 scale_x_continuous(breaks=scales::pretty_breaks(n = 3)) +
 scale_y_continuous(breaks=scales::pretty_breaks(n = 3))

p = ggarrange(p1, p2, p3, p4, labels = "AUTO", ncol = 1, nrow = 4)
annotate_figure(p,
 top = text_grob("Computational faithfulness and model sensitivity",
 face = "bold", size = 14))


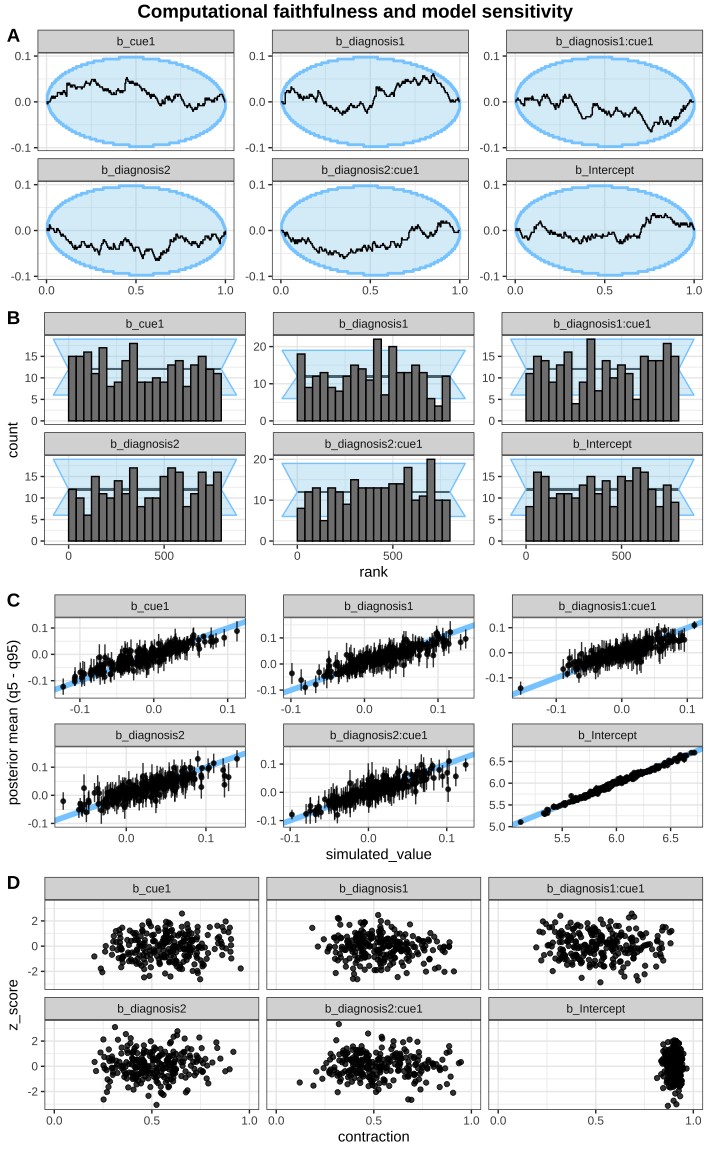


Rank histogramms, sample ECDF, the relationship between the simulated true parameters and the posterior estimates as well as z-score and posterior contraction of our population-level predictors all are acceptable for this model as well.

### 2.2.2 Posterior predictive checks

As the next step, we fit the model to the data, check whether there are divergence or rhat issues, and then check whether the chains have converged.

# fit the final model
set.seed(6824)
m.fab = brm(f.fab,
 df.fab, prior = priors,
 family = shifted_lognormal,
 iter = iter, warmup = warm,
 backend = "cmdstanr", threads = threading(8),
 file = "m_fab_final"
 )
rstan::check_hmc_diagnostics(m.fab$fit)

##
## Divergences:

## 0 of 8000 iterations ended with a divergence.

##
## Tree depth:

## 0 of 8000 iterations saturated the maximum tree depth of 10.

##
## Energy:

## E-BFMI indicated no pathological behavior.

# check that rhats are below 1.01
sum(brms::rhat(m.fab) >= 1.01, na.rm = T)

## [1] 49

# check the trace plots
post.draws = as_draws_df(m.fab)
mcmc_trace(post.draws, regex_pars = "^b_",
 facet_args = list(ncol = 3)) +
 scale_x_continuous(breaks=scales::pretty_breaks(n = 3)) +
 scale_y_continuous(breaks=scales::pretty_breaks(n = 3))

## Scale for x is already present.
## Adding another scale for x, which will replace the existing scale.


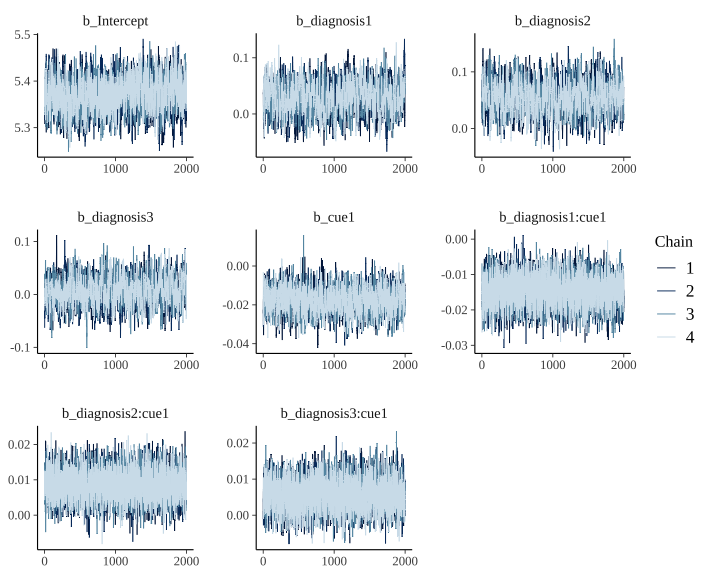


This model has no pathological behaviour with E-BFMI, no divergent samples and no rhat that is higher or equal to 1.01. Therefore, we go ahead and perform our posterior predictive checks.

# get posterior predictions
post.pred = posterior_predict(m.fab, ndraws = nsim)

# check the fit of the predicted data compared to the real data
p1 = pp_check(m.fab, ndraws = nsim) +
 theme_bw() + theme(legend.position = "none")

# distributions of means compared to the real values per group
p2 = ppc_stat_grouped(df.fab$rt.cor, post.pred, df.fab$diagnosis) +
 theme_bw() + theme(legend.position = "none")

# distributions of means compared to the real values per cue
p3 = ppc_stat_grouped(df.fab$rt.cor, post.pred, df.fab$cue) +
 theme_bw() + theme(legend.position = "none")

p = ggarrange(p1, p2, p3,
 nrow = 3, ncol = 1, labels = "AUTO")
annotate_figure(p,
 top = text_grob("Posterior predictive checks: RTs",
 face = "bold", size = 14))


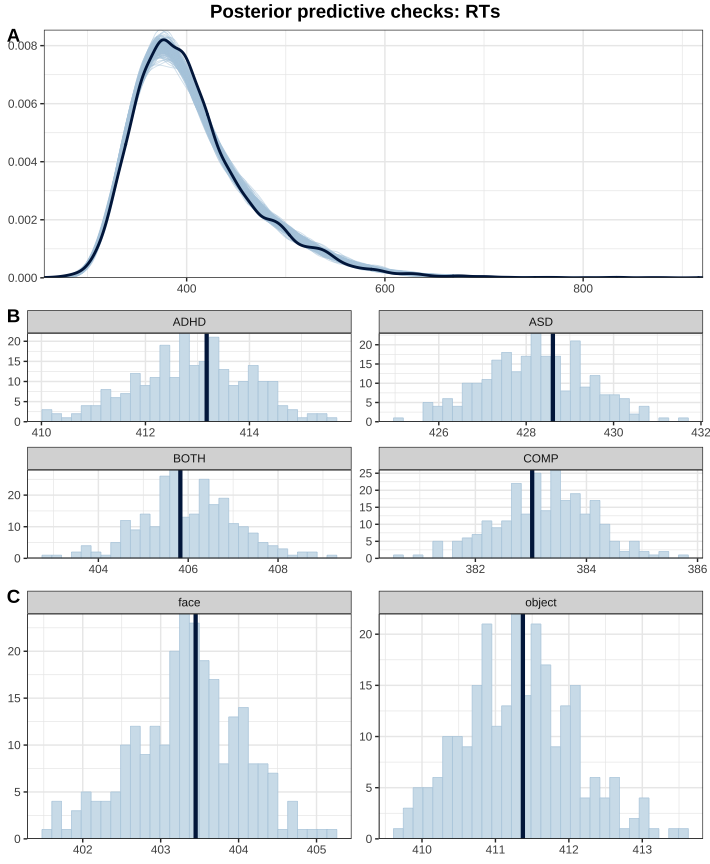


This model fits our data much better.

### 2.2.3 Inferences

Now that we are convinced that we can trust our model, we have a look at its estimate and use the hypothesis function to assess our hypotheses and perform explorative tests.

# print a summary
summary(m.fab)

## Family: shifted_lognormal
## Links: mu = identity; sigma = identity; ndt = identity
## Formula: rt.cor ~ diagnosis * cue + (cue | subID) + (cue * diagnosis | stm)
## Data: df.fab (Number of observations: 6624)
## Draws: 4 chains, each with iter = 3000; warmup = 1000; thin = 1;
## total post-warmup draws = 8000
##
## Multilevel Hyperparameters:
## ~stm (Number of levels: 36)
## Estimate Est.Error l-95% CI u-95% CI Rhat
## sd(Intercept) 0.03 0.00 0.02 0.04 1.00
## sd(cue1) 0.03 0.00 0.03 0.04 1.00
## sd(diagnosis1) 0.01 0.00 0.00 0.02 1.00
## sd(diagnosis2) 0.00 0.00 0.00 0.01 1.00
## sd(diagnosis3) 0.01 0.00 0.00 0.01 1.00
## sd(cue1:diagnosis1) 0.00 0.00 0.00 0.01 1.00
## sd(cue1:diagnosis2) 0.00 0.00 0.00 0.01 1.00
## sd(cue1:diagnosis3) 0.01 0.00 0.00 0.01 1.00
## cor(Intercept,cue1) -0.30 0.15 -0.58 0.02 1.00
## cor(Intercept,diagnosis1) -0.12 0.27 -0.60 0.43 1.00
## cor(cue1,diagnosis1) 0.01 0.26 -0.51 0.51 1.00
## cor(Intercept,diagnosis2) -0.09 0.29 -0.63 0.49 1.00
## cor(cue1,diagnosis2) -0.03 0.29 -0.58 0.53 1.00
## cor(diagnosis1,diagnosis2) -0.01 0.30 -0.59 0.57 1.00
## cor(Intercept,diagnosis3) 0.07 0.28 -0.48 0.60 1.00
## cor(cue1,diagnosis3) 0.17 0.27 -0.39 0.65 1.00
## cor(diagnosis1,diagnosis3) -0.13 0.31 -0.69 0.48 1.00
## cor(diagnosis2,diagnosis3) -0.07 0.30 -0.63 0.53 1.00
## cor(Intercept,cue1:diagnosis1) 0.03 0.28 -0.51 0.56 1.00
## cor(cue1,cue1:diagnosis1) -0.05 0.28 -0.57 0.49 1.00
## cor(diagnosis1,cue1:diagnosis1) -0.00 0.30 -0.57 0.57 1.00
## cor(diagnosis2,cue1:diagnosis1) 0.00 0.30 -0.57 0.57 1.00
## cor(diagnosis3,cue1:diagnosis1) -0.03 0.30 -0.61 0.56 1.00
## cor(Intercept,cue1:diagnosis2) -0.16 0.29 -0.67 0.44 1.00
## cor(cue1,cue1:diagnosis2) -0.05 0.28 -0.58 0.51 1.00
## cor(diagnosis1,cue1:diagnosis2) 0.04 0.30 -0.54 0.61 1.00
## cor(diagnosis2,cue1:diagnosis2) 0.04 0.31 -0.56 0.60 1.00
## cor(diagnosis3,cue1:diagnosis2) -0.05 0.30 -0.60 0.53 1.00
## cor(cue1:diagnosis1,cue1:diagnosis2) -0.07 0.31 -0.64 0.52 1.00
## cor(Intercept,cue1:diagnosis3) -0.12 0.28 -0.63 0.46 1.00
## cor(cue1,cue1:diagnosis3) -0.16 0.28 -0.64 0.43 1.00
## cor(diagnosis1,cue1:diagnosis3) 0.05 0.30 -0.52 0.61 1.00
## cor(diagnosis2,cue1:diagnosis3) 0.07 0.30 -0.52 0.62 1.00
## cor(diagnosis3,cue1:diagnosis3) -0.02 0.29 -0.58 0.55 1.00
## cor(cue1:diagnosis1,cue1:diagnosis3) -0.08 0.30 -0.65 0.52 1.00
## cor(cue1:diagnosis2,cue1:diagnosis3) -0.01 0.30 -0.57 0.56 1.00
## Bulk_ESS Tail_ESS
## sd(Intercept) 2724 4317
## sd(cue1) 2215 3985
## sd(diagnosis1) 2240 3682
## sd(diagnosis2) 4108 3660
## sd(diagnosis3) 3017 4012
## sd(cue1:diagnosis1) 3368 4350
## sd(cue1:diagnosis2) 3580 3611
## sd(cue1:diagnosis3) 2788 3253
## cor(Intercept,cue1) 1852 3479
## cor(Intercept,diagnosis1) 11906 5652
## cor(cue1,diagnosis1) 13168 5256
## cor(Intercept,diagnosis2) 15076 5534
## cor(cue1,diagnosis2) 13876 5843
## cor(diagnosis1,diagnosis2) 9969 6171
## cor(Intercept,diagnosis3) 12876 5591
## cor(cue1,diagnosis3) 12089 6067
## cor(diagnosis1,diagnosis3) 7225 6300
## cor(diagnosis2,diagnosis3) 6838 6402
## cor(Intercept,cue1:diagnosis1) 13820 5873
## cor(cue1,cue1:diagnosis1) 14433 6204
## cor(diagnosis1,cue1:diagnosis1) 8323 6155
## cor(diagnosis2,cue1:diagnosis1) 7080 6710
## cor(diagnosis3,cue1:diagnosis1) 7663 6743
## cor(Intercept,cue1:diagnosis2) 12437 5566
## cor(cue1,cue1:diagnosis2) 15034 6271
## cor(diagnosis1,cue1:diagnosis2) 9124 6070
## cor(diagnosis2,cue1:diagnosis2) 7129 6208
## cor(diagnosis3,cue1:diagnosis2) 6894 6281
## cor(cue1:diagnosis1,cue1:diagnosis2) 6020 6368
## cor(Intercept,cue1:diagnosis3) 10944 5628
## cor(cue1,cue1:diagnosis3) 11833 5556
## cor(diagnosis1,cue1:diagnosis3) 8457 6691
## cor(diagnosis2,cue1:diagnosis3) 7239 6504
## cor(diagnosis3,cue1:diagnosis3) 7829 6571
## cor(cue1:diagnosis1,cue1:diagnosis3) 6017 6727
## cor(cue1:diagnosis2,cue1:diagnosis3) 6320 6748
##
## ~subID (Number of levels: 92)
## Estimate Est.Error l-95% CI u-95% CI Rhat Bulk_ESS Tail_ESS
## sd(Intercept) 0.21 0.02 0.18 0.25 1.00 992 1981
## sd(cue1) 0.02 0.00 0.01 0.02 1.00 4100 4812
## cor(Intercept,cue1) -0.04 0.14 -0.31 0.25 1.00 6863 6208
##
## Regression Coefficients:
## Estimate Est.Error l-95% CI u-95% CI Rhat Bulk_ESS Tail_ESS
## Intercept 5.37 0.03 5.30 5.44 1.01 1112 2284
## diagnosis1 0.03 0.03 -0.02 0.08 1.00 875 1591
## diagnosis2 0.06 0.03 0.00 0.11 1.00 853 1715
## diagnosis3 0.01 0.03 -0.04 0.06 1.00 997 1817
## cue1 -0.02 0.01 -0.03 -0.01 1.00 2008 3129
## diagnosis1:cue1 -0.01 0.00 -0.02 -0.01 1.00 7114 6917
## diagnosis2:cue1 0.01 0.00 0.00 0.02 1.00 6998 6946
## diagnosis3:cue1 0.01 0.00 -0.00 0.01 1.00 7086 6813
##
## Further Distributional Parameters:
## Estimate Est.Error l-95% CI u-95% CI Rhat Bulk_ESS Tail_ESS
## sigma 0.13 0.00 0.13 0.14 1.00 5353 5447
## ndt 185.27 5.48 174.05 195.53 1.00 5293 5409
##
## Draws were sampled using sample(hmc). For each parameter, Bulk_ESS
## and Tail_ESS are effective sample size measures, and Rhat is the potential
## scale reduction factor on split chains (at convergence, Rhat = 1).

# get the estimates and compute groups
df.m.fab = as_draws_df(m.fab) %>%
 select(starts_with("b_")) %>%
 mutate(
 b_COMP = - b_diagnosis1 - b_diagnosis2 - b_diagnosis3,
 ASD = b_Intercept + b_diagnosis2,
 ADHD = b_Intercept + b_diagnosis1,
 BOTH = b_Intercept + b_diagnosis3,
 COMP = b_Intercept + b_COMP
 )

# plot the posterior distributions
df.m.fab %>%
 select(starts_with("b_")) %>%
 pivot_longer(cols = starts_with("b_"), names_to = "coef", values_to = "estimate") %>%
 filter(coef != "b_Intercept") %>%
 mutate(
 coef = case_match(coef,
 "b_diagnosis1" ~ "ADHD",
 "b_diagnosis2" ~ "ASD",
 "b_diagnosis3" ~ "ADHD+ASD",
 "b_COMP" ~ "COMP",
 "b_cue1" ~ "Face",
 "b_diagnosis1:cue1" ~ "Interaction: ADHD",
 "b_diagnosis2:cue1" ~ "Interaction: ASD",
 "b_diagnosis3:cue1" ~ "Interaction: ADHD+ASD"
 ),
 coef = fct_reorder(coef, desc(estimate))
 ) %>%
 group_by(coef) %>%
 mutate(
 cred = case_when(
 (mean(estimate) < 0 & quantile(estimate, probs = 0.975) < 0) |
 (mean(estimate) > 0 & quantile(estimate, probs = 0.025) > 0) ~ "credible",
 T ~ "not credible"
 )
 ) %>% ungroup() %>%
 ggplot(aes(x = estimate, y = coef, fill = cred)) +
 geom_vline(xintercept = 0, linetype = 'dashed') +
 ggdist::stat_halfeye(alpha = 0.7) + ylab(NULL) + theme_bw() +
 scale_fill_manual(values = c(credible = c_dark, c_light)) +
 theme(legend.position = "none")


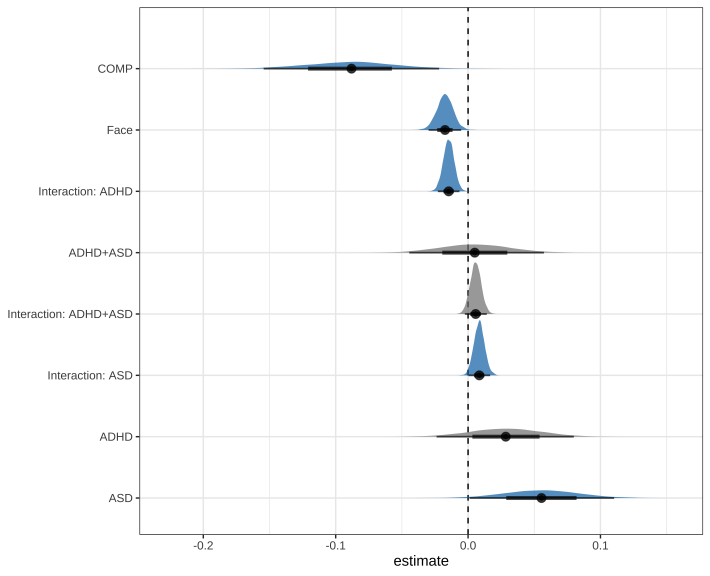


# H1a: FAB effect in COMP
h1a = hypothesis(m.fab,
 "0 < 2*(diagnosis1:cue1 + diagnosis2:cue1 + diagnosis3:cue1 - cue1)")
h1a

## Hypothesis Tests for class b:
## Hypothesis Estimate Est.Error CI.Lower CI.Upper Evid.Ratio
## 1 (0)-(2*(diagnosis... < 0 -0.03 0.02 -0.06 -0.01 66.8
## Post.Prob Star
## 1 0.99 *
## ---
## 'CI': 90%-CI for one-sided and 95%-CI for two-sided hypotheses.
## '*': For one-sided hypotheses, the posterior probability exceeds 95%;
## for two-sided hypotheses, the value tested against lies outside the 95%-CI.
## Posterior probabilities of point hypotheses assume equal prior probabilities.

# H1b: ADHD slower than COMP
h1b = hypothesis(m.fab,
 "0 < 2*diagnosis1 + diagnosis2 + diagnosis3")
h1b

## Hypothesis Tests for class b:
## Hypothesis Estimate Est.Error CI.Lower CI.Upper Evid.Ratio
## 1 (0)-(2*diagnosis1... < 0 -0.12 0.05 -0.2 -0.03 100.27
## Post.Prob Star
## 1 0.99 *
## ---
## 'CI': 90%-CI for one-sided and 95%-CI for two-sided hypotheses.
## '*': For one-sided hypotheses, the posterior probability exceeds 95%;
## for two-sided hypotheses, the value tested against lies outside the 95%-CI.
## Posterior probabilities of point hypotheses assume equal prior probabilities.

# H1c: ASD slower than COMP
h1c = hypothesis(m.fab,
 "0 < 2*diagnosis2 + diagnosis1 + diagnosis3")
h1c

## Hypothesis Tests for class b:
## Hypothesis Estimate Est.Error CI.Lower CI.Upper Evid.Ratio
## 1 (0)-(2*diagnosis2... < 0 -0.14 0.05 -0.23 -0.06 189.48
## Post.Prob Star
## 1 0.99 *
## ---
## 'CI': 90%-CI for one-sided and 95%-CI for two-sided hypotheses.
## '*': For one-sided hypotheses, the posterior probability exceeds 95%;
## for two-sided hypotheses, the value tested against lies outside the 95%-CI.
## Posterior probabilities of point hypotheses assume equal prior probabilities.

# H1d: FAB in ASD decreased compared to COMP
h1d = hypothesis(m.fab,
 "0 < 4*diagnosis2:cue1 + 2*diagnosis1:cue1 + 2*diagnosis3:cue1")
h1d

## Hypothesis Tests for class b:
## Hypothesis Estimate Est.Error CI.Lower CI.Upper Evid.Ratio
## 1 (0)-(4*diagnosis2... < 0 -0.02 0.01 -0.04 0.01 7.02
## Post.Prob Star
## 1 0.88
## ---
## 'CI': 90%-CI for one-sided and 95%-CI for two-sided hypotheses.
## '*': For one-sided hypotheses, the posterior probability exceeds 95%;
## for two-sided hypotheses, the value tested against lies outside the 95%-CI.
## Posterior probabilities of point hypotheses assume equal prior probabilities.

# H1e: FAB in ADHD differs from FAB in COMP (undirected)
h1e = hypothesis(m.fab,
 "0 > 4*diagnosis1:cue1 + 2*diagnosis2:cue1 + 2*diagnosis3:cue1",
 alpha = 0.025)
h1e

## Hypothesis Tests for class b:
## Hypothesis Estimate Est.Error CI.Lower CI.Upper Evid.Ratio
## 1 (0)-(4*diagnosis1... > 0 0.03 0.01 0 0.06 65.12
## Post.Prob Star
## 1 0.98 *
## ---
## 'CI': 95%-CI for one-sided and 97.5%-CI for two-sided hypotheses.
## '*': For one-sided hypotheses, the posterior probability exceeds 97.5%;
## for two-sided hypotheses, the value tested against lies outside the 97.5%-CI.
## Posterior probabilities of point hypotheses assume equal prior probabilities.

# Exploration

# E1: FAB generally
e1 = hypothesis(m.fab, "2*cue1 < 0", alpha = 0.025)
e1

## Hypothesis Tests for class b:
## Hypothesis Estimate Est.Error CI.Lower CI.Upper Evid.Ratio Post.Prob Star
## 1 (2*cue1) < 0 -0.03 0.01 -0.06 -0.01 379.95 1 *
## ---
## 'CI': 95%-CI for one-sided and 97.5%-CI for two-sided hypotheses.
## '*': For one-sided hypotheses, the posterior probability exceeds 97.5%;
## for two-sided hypotheses, the value tested against lies outside the 97.5%-CI.
## Posterior probabilities of point hypotheses assume equal prior probabilities.

# E2: FAB effect in ADHD
e2 = hypothesis(m.fab, "0 < -2*cue1 - 2*diagnosis1:cue1", alpha = 0.025)
e2

## Hypothesis Tests for class b:
## Hypothesis Estimate Est.Error CI.Lower CI.Upper Evid.Ratio
## 1 (0)-(-2*cue1-2*di... < 0 -0.06 0.01 -0.09 -0.04 Inf
## Post.Prob Star
## 1 1 *
## ---
## 'CI': 95%-CI for one-sided and 97.5%-CI for two-sided hypotheses.
## '*': For one-sided hypotheses, the posterior probability exceeds 97.5%;
## for two-sided hypotheses, the value tested against lies outside the 97.5%-CI.
## Posterior probabilities of point hypotheses assume equal prior probabilities.

# E3: FAB effect in ASD
e3 = hypothesis(m.fab, "0 < -2*cue1 - 2*diagnosis2:cue1", alpha = 0.025)
e3

## Hypothesis Tests for class b:
## Hypothesis Estimate Est.Error CI.Lower CI.Upper Evid.Ratio
## 1 (0)-(-2*cue1-2*di... < 0 -0.02 0.01 -0.05 0.01 7.89
## Post.Prob Star
## 1 0.89
## ---
## 'CI': 95%-CI for one-sided and 97.5%-CI for two-sided hypotheses.
## '*': For one-sided hypotheses, the posterior probability exceeds 97.5%;
## for two-sided hypotheses, the value tested against lies outside the 97.5%-CI.
## Posterior probabilities of point hypotheses assume equal prior probabilities.

# E4: FAB effect in ADHD+ASD
e4 = hypothesis(m.fab, "0 < -2*cue1 - 2*diagnosis3:cue1", alpha = 0.025)
e4

## Hypothesis Tests for class b:
## Hypothesis Estimate Est.Error CI.Lower CI.Upper Evid.Ratio
## 1 (0)-(-2*cue1-2*di... < 0 -0.02 0.01 -0.05 0.01 16.94
## Post.Prob Star
## 1 0.94
## ---
## 'CI': 95%-CI for one-sided and 97.5%-CI for two-sided hypotheses.
## '*': For one-sided hypotheses, the posterior probability exceeds 97.5%;
## for two-sided hypotheses, the value tested against lies outside the 97.5%-CI.
## Posterior probabilities of point hypotheses assume equal prior probabilities.

# E5: FAB in ADHD differs from FAB in ASD
e5 = hypothesis(m.fab,
 "0 < -2*diagnosis1:cue1 + 2*diagnosis2:cue1", alpha = 0.025)
e5

## Hypothesis Tests for class b:
## Hypothesis Estimate Est.Error CI.Lower CI.Upper Evid.Ratio
## 1 (0)-(-2*diagnosis... < 0 -0.05 0.01 -0.07 -0.02 1141.86
## Post.Prob Star
## 1 1 *
## ---
## 'CI': 95%-CI for one-sided and 97.5%-CI for two-sided hypotheses.
## '*': For one-sided hypotheses, the posterior probability exceeds 97.5%;
## for two-sided hypotheses, the value tested against lies outside the 97.5%-CI.
## Posterior probabilities of point hypotheses assume equal prior probabilities.

# E6: FAB in ADHD differs from FAB in BOTH
e6 = hypothesis(m.fab,
 "0 < -2*diagnosis1:cue1 + 2*diagnosis3:cue1", alpha = 0.025)
e6

## Hypothesis Tests for class b:
## Hypothesis Estimate Est.Error CI.Lower CI.Upper Evid.Ratio
## 1 (0)-(-2*diagnosis... < 0 -0.04 0.01 -0.07 -0.01 570.43
## Post.Prob Star
## 1 1 *
## ---
## 'CI': 95%-CI for one-sided and 97.5%-CI for two-sided hypotheses.
## '*': For one-sided hypotheses, the posterior probability exceeds 97.5%;
## for two-sided hypotheses, the value tested against lies outside the 97.5%-CI.
## Posterior probabilities of point hypotheses assume equal prior probabilities.

# E7: FAB in ASD differs from FAB in BOTH
e7 = hypothesis(m.fab,
 "0 > -2*diagnosis2:cue1 + 2*diagnosis3:cue1", alpha = 0.025)
e7

## Hypothesis Tests for class b:
## Hypothesis Estimate Est.Error CI.Lower CI.Upper Evid.Ratio
## 1 (0)-(-2*diagnosis... > 0 0.01 0.01 -0.02 0.03 1.95
## Post.Prob Star
## 1 0.66
## ---
## 'CI': 95%-CI for one-sided and 97.5%-CI for two-sided hypotheses.
## '*': For one-sided hypotheses, the posterior probability exceeds 97.5%;
## for two-sided hypotheses, the value tested against lies outside the 97.5%-CI.
## Posterior probabilities of point hypotheses assume equal prior probabilities.

# E8: FAB in COMP differs from FAB in BOTH
e8 = hypothesis(m.fab,
 "0 < 2*diagnosis1:cue1 + 2*diagnosis2:cue1 + 4*diagnosis3:cue1",
 alpha = 0.025)
e8

## Hypothesis Tests for class b:
## Hypothesis Estimate Est.Error CI.Lower CI.Upper Evid.Ratio
## 1 (0)-(2*diagnosis1... < 0 -0.01 0.01 -0.04 0.02 3.37
## Post.Prob Star
## 1 0.77
## ---
## 'CI': 95%-CI for one-sided and 97.5%-CI for two-sided hypotheses.
## '*': For one-sided hypotheses, the posterior probability exceeds 97.5%;
## for two-sided hypotheses, the value tested against lies outside the 97.5%-CI.
## Posterior probabilities of point hypotheses assume equal prior probabilities.

# E9: face in COMP versus face in ADHD
e9 = hypothesis(m.fab,
 "0 < 2*diagnosis1 + diagnosis2 + diagnosis3 +
 2*diagnosis1:cue1 + diagnosis2:cue1 + diagnosis3:cue1",
 alpha = 0.025)
e9

## Hypothesis Tests for class b:
## Hypothesis Estimate Est.Error CI.Lower CI.Upper Evid.Ratio
## 1 (0)-(2*diagnosis1... < 0 -0.1 0.05 -0.2 0 44.45
## Post.Prob Star
## 1 0.98 *
## ---
## 'CI': 95%-CI for one-sided and 97.5%-CI for two-sided hypotheses.
## '*': For one-sided hypotheses, the posterior probability exceeds 97.5%;
## for two-sided hypotheses, the value tested against lies outside the 97.5%-CI.
## Posterior probabilities of point hypotheses assume equal prior probabilities.

# E10: object in COMP versus object in ADHD
e10 = hypothesis(m.fab,
 "0 < 2*diagnosis1 + diagnosis2 + diagnosis3 -
 (2*diagnosis1:cue1 + diagnosis2:cue1 + diagnosis3:cue1)",
 alpha = 0.025)
e10

## Hypothesis Tests for class b:
## Hypothesis Estimate Est.Error CI.Lower CI.Upper Evid.Ratio
## 1 (0)-(2*diagnosis1... < 0 -0.13 0.05 -0.23 -0.03 189.48
## Post.Prob Star
## 1 0.99 *
## ---
## 'CI': 95%-CI for one-sided and 97.5%-CI for two-sided hypotheses.
## '*': For one-sided hypotheses, the posterior probability exceeds 97.5%;
## for two-sided hypotheses, the value tested against lies outside the 97.5%-CI.
## Posterior probabilities of point hypotheses assume equal prior probabilities.

# E11: E10 > E9
e11 = hypothesis(m.fab,
 "2*diagnosis1 + diagnosis2 + diagnosis3 -
 (2*diagnosis1:cue1 + diagnosis2:cue1 + diagnosis3:cue1) >
 2*diagnosis1 + diagnosis2 + diagnosis3 +
 (2*diagnosis1:cue1 + diagnosis2:cue1 + diagnosis3:cue1)",
 alpha = 0.025)
e11

## Hypothesis Tests for class b:
## Hypothesis Estimate Est.Error CI.Lower CI.Upper Evid.Ratio
## 1 (2*diagnosis1+dia... > 0 0.03 0.01 0 0.06 65.12
## Post.Prob Star
## 1 0.98 *
## ---
## 'CI': 95%-CI for one-sided and 97.5%-CI for two-sided hypotheses.
## '*': For one-sided hypotheses, the posterior probability exceeds 97.5%;
## for two-sided hypotheses, the value tested against lies outside the 97.5%-CI.
## Posterior probabilities of point hypotheses assume equal prior probabilities.

# extract predicted differences in ms instead of log data
df.new = df.fab %>%
 select(diagnosis, cue) %>%
 distinct() %>%
 mutate(
 condition = paste(diagnosis, cue, sep = "_")
 )
df.ms = as.data.frame(
 fitted(m.fab, summary = F,
 newdata = df.new %>% select(diagnosis, cue),
 re_formula = NA))
colnames(df.ms) = df.new$condition

st(df.ms,
 summ = c('mean(x)','sd(x)','min(x)','pctile(x)[2.5]',
 'pctile(x)[97.5]','max(x)'))

Summary Statistics

| Variable | Mean | Sd | Min | Pctile[2.5] | Pctile[97.5] | Max |
| --- | --- | --- | --- | --- | --- | --- |
| ADHD_face | 402 | 7.6 | 378 | 387 | 416 | 428 |
| ADHD_object | 416 | 8.2 | 388 | 399 | 432 | 446 |
| COMP_face | 381 | 8 | 352 | 365 | 396 | 414 |
| COMP_object | 387 | 8.5 | 360 | 371 | 404 | 421 |
| ASD_face | 413 | 8.1 | 384 | 397 | 429 | 447 |
| ASD_object | 417 | 8.3 | 387 | 401 | 433 | 448 |
| BOTH_face | 401 | 7.5 | 377 | 387 | 415 | 438 |
| BOTH_object | 406 | 7.8 | 383 | 391 | 421 | 446 |

# calculate our difference columns
df.ms = df.ms %>%
 mutate(
 COMP = rowMeans(select(., matches("COMP_.*")), na.rm = T),
 ADHD = rowMeans(select(., matches("ADHD_.*")), na.rm = T),
 ASD = rowMeans(select(., matches("ASD_.*")), na.rm = T),
 BOTH = rowMeans(select(., matches("BOTH_.*")), na.rm = T),
 FAB = rowMeans(select(., matches(".*_object")), na.rm = T) -
 rowMeans(select(., matches(".*_face")), na.rm = T),
 FAB_COMP = COMP_object - COMP_face,
 FAB_ADHD = ADHD_object - ADHD_face,
 FAB_ASD = ASD_object - ASD_face,
 FAB_BOTH = BOTH_object - BOTH_face,
 h1b = ADHD - COMP,
 h1c = ASD - COMP,
 h1d = FAB_COMP - FAB_ASD,
 h1e = FAB_ADHD - FAB_COMP,
 BOTH_COMP = BOTH - COMP
 )

st(df.ms %>%
 mutate(
 face = rowMeans(select(., matches(".*_face")), na.rm = T),
 object = rowMeans(select(., matches(".*_object")), na.rm = T),
 ADHDvCOMP_face = ADHD_face - COMP_face,
 ADHDvCOMP_object = ADHD_object - COMP_object,
 diff_ADHDvCOMP = ADHDvCOMP_object - ADHDvCOMP_face
 ) %>% select(face, object, FAB, ADHDvCOMP_face, ADHDvCOMP_object, diff_ADHDvCOMP),
 summ = c('mean(x)','sd(x)','min(x)','pctile(x)[2.5]','pctile(x)[97.5]','max(x)'))

Summary Statistics

| Variable | Mean | Sd | Min | Pctile[2.5] | Pctile[97.5] | Max |
| --- | --- | --- | --- | --- | --- | --- |
| face | 399 | 4.8 | 380 | 390 | 408 | 418 |
| object | 407 | 5.2 | 390 | 397 | 417 | 425 |
| FAB | 7.6 | 2.7 | -6.5 | 1.9 | 13 | 18 |
| ADHDvCOMP_face | 21 | 10 | -20 | -0.37 | 40 | 62 |
| ADHDvCOMP_object | 29 | 11 | -12 | 6.3 | 49 | 74 |
| diff_ADHDvCOMP | 7.6 | 3 | -3.3 | 1.5 | 13 | 18 |

As hypothesised, both autistic adults and adults with ADHD exhibited increased overall reaction times compared with the COMP group (COMP - ADHD: *estimate* = -0.12 [-0.2, -0.03], *posterior probability* = 99.01%; COMP - ASD: *estimate* = -0.14 [-0.23, -0.06], *posterior probability* = 99.48%). The model predicts that participants in the comparison group react 24.74ms [4.15, 45.24] faster than the participants in the ADHD group and 30.78ms [8.27, 52.27] faster than autistic participants. Additionally, the model predicts that the participants in the comparison group react 19.54ms [-0.89, 40.35] faster than adults in the ADHD+ASD group.

Our Bayesian linear mixed model with the median of correct reaction times as the outcome and diagnostic status, cue (face or object) and their interaction confirmed a face attention bias in our comparison group: COMP participants reacted faster in response to targets appearing on the side of the face compared to targets appearing on the side of the object (*estimate* = -0.03 [-0.06, -0.01], *posterior probability* = 98.52%). FAB was not credibly decreased in ASD participants compared to COMP participants (*estimate* = -0.02 [-0.04, 0.01], *posterior probability* = 87.52%). However, FAB was credibly higher in the ADHD than the COMP group (*estimate* = 0.03 [0, 0.06], *posterior probability* = 98.49%). Specifically, predicted reaction times based on the model estimate a FAB of 6.77ms [0.78, 12.98] in the COMP group, 14.35ms [7.89, 20.93] in the ADHD group, 4.13ms [-2.58, 10.82] in the ASD group as well as 5.12ms [-1.21, 11.46] in the ADHD+ASD group. These estimates are reflected in our exploration of FAB over all groups (*estimate* = -0.03 [-0.06, -0.01], *posterior probability* = 99.74%) as well as in the separate clinical groups with our model revealing a credible FAB effect in the ADHD (*estimate* = -0.06 [-0.09, -0.04], *posterior probability* = 100%) but not the ASD (*estimate* = -0.02 [-0.05, 0.01], *posterior probability* = 88.75%) and the ADHD+ASD group (*estimate* = -0.02 [-0.05, 0.01], *posterior probability* = 94.42%).

Exploration regarding comparison between ADHD and COMP on face and object separately:

- Face: *estimate* = -0.1 [-0.2, 0], *posterior probability* = 97.8%
- Object: *estimate* = -0.13 [-0.23, -0.03], *posterior probability* = 99.48%
- Object(ADHD-COMP) > Face(ADHD-COMP): *estimate* = 0.03 [0, 0.06], *posterior probability* = 98.49%

## 2.3 Plots

# overall median reaction times
p = df.fab %>%
 group_by(subID, diagnosis, cue) %>%
 summarise(
 rt.cor = mean(rt.cor, na.rm = T)
 ) %>%
 mutate(
 diagnosis = recode(diagnosis, "BOTH" = "ADHD+ASD"),
 Target = recode(cue, "face" = "Face-cued", "object" = "Object-cued")
 ) %>%
 ggplot(aes(diagnosis, rt.cor, fill = Target, colour = Target)) + #
 geom_rain(rain.side = 'r',
boxplot.args = list(color = "black", outlier.shape = NA, show_guide = FALSE, alpha = .8),
violin.args = list(color = "black", outlier.shape = NA, alpha = .8),
boxplot.args.pos = list(
 position = ggpp::position_dodgenudge(x = 0, width = 0.3), width = 0.3
),
point.args = list(show_guide = FALSE, alpha = .5),
violin.args.pos = list(
 width = 0.6, position = position_nudge(x = 0.16)),
point.args.pos = list(position = ggpp::position_dodgenudge(x = -0.25, width = 0.1))) +
 ylim(0, 700) +
 scale_fill_manual(values = custom.col2) +
 scale_color_manual(values = custom.col2) +
 labs(title = "",
 x = "",
 y = "Reaction time (ms)") +
 theme_bw() +
 theme(legend.position = "inside", legend.position.inside = c(0.5, 0.2),
 plot.title = element_text(hjust = 0.5),
 legend.direction = "horizontal",
 legend.title=element_blank(), axis.title.y = element_text(size = 28),
 axis.text.x = element_text(size = 28, colour = "black"),
 axis.text.y = element_text(size = 24),
 text = element_text(family = "Merriweather", size = 32))

ggsave("Fig3_rts.tif", plot = p,
 units = "mm",
 width = 170,
 height = 100,
 dpi = 300)

# 3 Explorative analysis of RTs considering number of saccades

Since saccadic behaviour may have influenced reaction times, we rerun the model concerning reaction times with a separate predictor coding the number of saccades of this stimulus pair.

# merge behaviour and saccades together
df.sac.fab = merge(df.fab.full,
 df.sac %>% group_by(subID, diagnosis, trl) %>% summarise(n.sac = n()),
 all.x = T) %>%
 # compute median rt.cor
 group_by(subID, diagnosis, cue, stm) %>%
 summarise(
 rt.cor = median(rt.cor, na.rm = T),
 n.sac = sum(n.sac, na.rm = T)
 )

## `summarise()` has grouped output by 'subID', 'diagnosis'. You can override
## using the `.groups` argument.
## `summarise()` has grouped output by 'subID', 'diagnosis', 'cue'. You can
## override using the `.groups` argument.

# set the contrasts
contrasts(df.sac.fab$cue) = contr.sum(2)
contrasts(df.sac.fab$cue)

## [,1]
## face 1
## object -1

contrasts(df.sac.fab$diagnosis) = contr.sum(4)
contrasts(df.sac.fab$diagnosis)

## [,1] [,2] [,3]
## ADHD 1 0 0
## ASD 0 1 0
## BOTH 0 0 1
## COMP -1 -1 -1

# run the model > more iterations due to some suboptimal rhats in the first try
set.seed(1357)
m.rtsac = brm(rt.cor ~ diagnosis * cue + n.sac + (cue | subID) + (cue * diagnosis | stm),
 df.sac.fab, prior = priors,
 family = shifted_lognormal,
 iter = iter*2, warmup = warm*2,
 backend = "cmdstanr", threads = threading(8), file = "m_fab_sac"
 )
rstan::check_hmc_diagnostics(m.rtsac$fit)

##
## Divergences:

## 0 of 16000 iterations ended with a divergence.

##
## Tree depth:

## 0 of 16000 iterations saturated the maximum tree depth of 10.

##
## Energy:

## E-BFMI indicated no pathological behavior.

# check that rhats are below 1.01
sum(brms::rhat(m.rtsac) >= 1.01, na.rm = T)

## [1] 0

# check the trace plots
post.draws = as_draws_df(m.rtsac)
mcmc_trace(post.draws, regex_pars = "^b_",
 facet_args = list(ncol = 3)) +
 scale_x_continuous(breaks=scales::pretty_breaks(n = 3)) +
 scale_y_continuous(breaks=scales::pretty_breaks(n = 3))

## Scale for x is already present.
## Adding another scale for x, which will replace the existing scale.


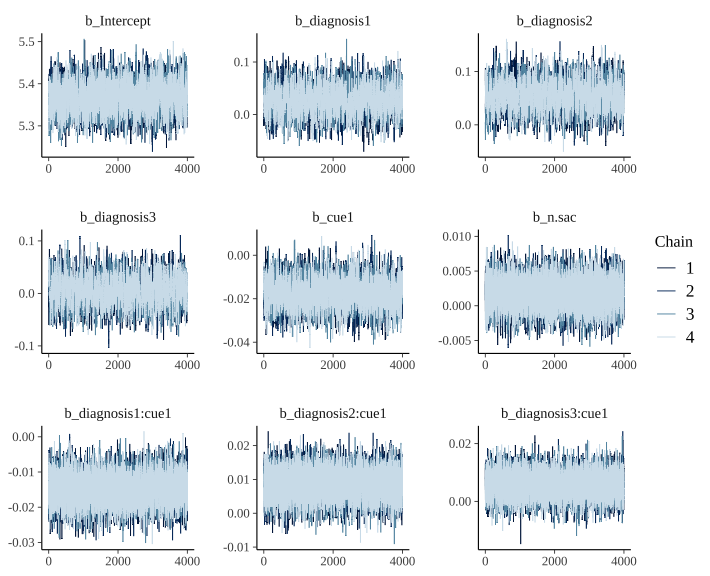


# get posterior predictions
post.pred = posterior_predict(m.rtsac, ndraws = nsim)

# check the fit of the predicted data compared to the real data
p1 = pp_check(m.rtsac, ndraws = nsim) +
 theme_bw() + theme(legend.position = "none")

df.sac.fab = df.sac.fab %>% drop_na()

# distributions of means compared to the real values per group
p2 = ppc_stat_grouped(df.sac.fab$rt.cor, post.pred, df.sac.fab$diagnosis) +
 theme_bw() + theme(legend.position = "none")

p = ggarrange(p1, p2,
 nrow = 2, ncol = 1, labels = "AUTO")
annotate_figure(p,
 top = text_grob("Posterior predictive checks: RTs of trials with saccade covariate",
 face = "bold", size = 14))


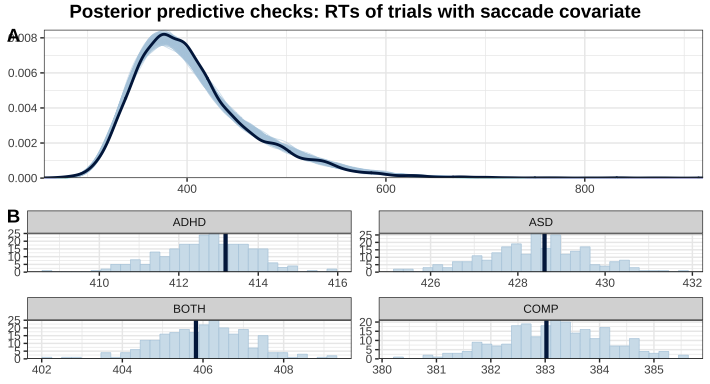


# print a summary
summary(m.rtsac)

## Family: shifted_lognormal
## Links: mu = identity; sigma = identity; ndt = identity
## Formula: rt.cor ~ diagnosis * cue + n.sac + (cue | subID) + (cue * diagnosis | stm)
## Data: df.sac.fab (Number of observations: 6624)
## Draws: 4 chains, each with iter = 6000; warmup = 2000; thin = 1;
## total post-warmup draws = 16000
##
## Multilevel Hyperparameters:
## ~stm (Number of levels: 36)
## Estimate Est.Error l-95% CI u-95% CI Rhat
## sd(Intercept) 0.03 0.00 0.02 0.04 1.00
## sd(cue1) 0.03 0.00 0.03 0.04 1.00
## sd(diagnosis1) 0.01 0.00 0.00 0.02 1.00
## sd(diagnosis2) 0.00 0.00 0.00 0.01 1.00
## sd(diagnosis3) 0.01 0.00 0.00 0.01 1.00
## sd(cue1:diagnosis1) 0.00 0.00 0.00 0.01 1.00
## sd(cue1:diagnosis2) 0.00 0.00 0.00 0.01 1.00
## sd(cue1:diagnosis3) 0.01 0.00 0.00 0.01 1.00
## cor(Intercept,cue1) -0.31 0.15 -0.58 0.01 1.00
## cor(Intercept,diagnosis1) -0.12 0.26 -0.59 0.42 1.00
## cor(cue1,diagnosis1) 0.01 0.26 -0.50 0.50 1.00
## cor(Intercept,diagnosis2) -0.09 0.29 -0.62 0.50 1.00
## cor(cue1,diagnosis2) -0.04 0.29 -0.58 0.54 1.00
## cor(diagnosis1,diagnosis2) -0.01 0.30 -0.58 0.57 1.00
## cor(Intercept,diagnosis3) 0.07 0.28 -0.48 0.58 1.00
## cor(cue1,diagnosis3) 0.17 0.28 -0.41 0.65 1.00
## cor(diagnosis1,diagnosis3) -0.13 0.31 -0.68 0.49 1.00
## cor(diagnosis2,diagnosis3) -0.07 0.30 -0.63 0.53 1.00
## cor(Intercept,cue1:diagnosis1) 0.03 0.29 -0.52 0.58 1.00
## cor(cue1,cue1:diagnosis1) -0.04 0.28 -0.58 0.51 1.00
## cor(diagnosis1,cue1:diagnosis1) 0.00 0.30 -0.57 0.57 1.00
## cor(diagnosis2,cue1:diagnosis1) 0.01 0.30 -0.57 0.58 1.00
## cor(diagnosis3,cue1:diagnosis1) -0.02 0.30 -0.59 0.56 1.00
## cor(Intercept,cue1:diagnosis2) -0.16 0.29 -0.67 0.44 1.00
## cor(cue1,cue1:diagnosis2) -0.05 0.28 -0.57 0.51 1.00
## cor(diagnosis1,cue1:diagnosis2) 0.03 0.30 -0.54 0.59 1.00
## cor(diagnosis2,cue1:diagnosis2) 0.04 0.30 -0.55 0.61 1.00
## cor(diagnosis3,cue1:diagnosis2) -0.05 0.30 -0.60 0.54 1.00
## cor(cue1:diagnosis1,cue1:diagnosis2) -0.07 0.31 -0.64 0.54 1.00
## cor(Intercept,cue1:diagnosis3) -0.12 0.28 -0.62 0.45 1.00
## cor(cue1,cue1:diagnosis3) -0.16 0.27 -0.65 0.40 1.00
## cor(diagnosis1,cue1:diagnosis3) 0.06 0.30 -0.53 0.61 1.00
## cor(diagnosis2,cue1:diagnosis3) 0.07 0.30 -0.53 0.62 1.00
## cor(diagnosis3,cue1:diagnosis3) -0.01 0.30 -0.58 0.56 1.00
## cor(cue1:diagnosis1,cue1:diagnosis3) -0.08 0.31 -0.64 0.53 1.00
## cor(cue1:diagnosis2,cue1:diagnosis3) -0.01 0.30 -0.57 0.56 1.00
## Bulk_ESS Tail_ESS
## sd(Intercept) 5517 9259
## sd(cue1) 4842 9387
## sd(diagnosis1) 4157 5899
## sd(diagnosis2) 8220 8076
## sd(diagnosis3) 5643 7912
## sd(cue1:diagnosis1) 6971 8262
## sd(cue1:diagnosis2) 7929 7331
## sd(cue1:diagnosis3) 6451 7329
## cor(Intercept,cue1) 3458 6962
## cor(Intercept,diagnosis1) 23743 11429
## cor(cue1,diagnosis1) 25498 12290
## cor(Intercept,diagnosis2) 30347 12059
## cor(cue1,diagnosis2) 30653 12458
## cor(diagnosis1,diagnosis2) 22011 11736
## cor(Intercept,diagnosis3) 26342 11764
## cor(cue1,diagnosis3) 24760 11835
## cor(diagnosis1,diagnosis3) 13936 12640
## cor(diagnosis2,diagnosis3) 14508 13143
## cor(Intercept,cue1:diagnosis1) 28278 11416
## cor(cue1,cue1:diagnosis1) 30576 12352
## cor(diagnosis1,cue1:diagnosis1) 18194 13379
## cor(diagnosis2,cue1:diagnosis1) 15612 12785
## cor(diagnosis3,cue1:diagnosis1) 14525 13518
## cor(Intercept,cue1:diagnosis2) 25666 11945
## cor(cue1,cue1:diagnosis2) 28313 11313
## cor(diagnosis1,cue1:diagnosis2) 20782 12759
## cor(diagnosis2,cue1:diagnosis2) 16363 13241
## cor(diagnosis3,cue1:diagnosis2) 15503 13335
## cor(cue1:diagnosis1,cue1:diagnosis2) 12375 13060
## cor(Intercept,cue1:diagnosis3) 25460 11935
## cor(cue1,cue1:diagnosis3) 23560 12516
## cor(diagnosis1,cue1:diagnosis3) 19594 12131
## cor(diagnosis2,cue1:diagnosis3) 14364 13359
## cor(diagnosis3,cue1:diagnosis3) 14322 12731
## cor(cue1:diagnosis1,cue1:diagnosis3) 12229 13830
## cor(cue1:diagnosis2,cue1:diagnosis3) 12440 13054
##
## ~subID (Number of levels: 92)
## Estimate Est.Error l-95% CI u-95% CI Rhat Bulk_ESS Tail_ESS
## sd(Intercept) 0.21 0.02 0.18 0.25 1.00 2454 4627
## sd(cue1) 0.02 0.00 0.01 0.02 1.00 7352 10575
## cor(Intercept,cue1) -0.03 0.14 -0.31 0.25 1.00 16266 13844
##
## Regression Coefficients:
## Estimate Est.Error l-95% CI u-95% CI Rhat Bulk_ESS Tail_ESS
## Intercept 5.37 0.03 5.30 5.44 1.00 2078 4770
## diagnosis1 0.03 0.03 -0.02 0.08 1.00 1902 3867
## diagnosis2 0.06 0.03 0.00 0.11 1.00 1892 3726
## diagnosis3 0.01 0.03 -0.05 0.06 1.00 1860 3959
## cue1 -0.02 0.01 -0.03 -0.01 1.00 3932 6920
## n.sac 0.00 0.00 -0.00 0.01 1.00 33237 11017
## diagnosis1:cue1 -0.01 0.00 -0.02 -0.01 1.00 14772 13354
## diagnosis2:cue1 0.01 0.00 0.00 0.02 1.00 14556 12851
## diagnosis3:cue1 0.01 0.00 -0.00 0.01 1.00 14891 12256
##
## Further Distributional Parameters:
## Estimate Est.Error l-95% CI u-95% CI Rhat Bulk_ESS Tail_ESS
## sigma 0.13 0.00 0.13 0.14 1.00 10674 11494
## ndt 185.49 5.47 174.29 195.80 1.00 10487 10634
##
## Draws were sampled using sample(hmc). For each parameter, Bulk_ESS
## and Tail_ESS are effective sample size measures, and Rhat is the potential
## scale reduction factor on split chains (at convergence, Rhat = 1).

# H1a: FAB effect in COMP
h1a = hypothesis(m.rtsac,
 "0 < 2*(diagnosis1:cue1 + diagnosis2:cue1 + diagnosis3:cue1 - cue1)")
h1a

## Hypothesis Tests for class b:
## Hypothesis Estimate Est.Error CI.Lower CI.Upper Evid.Ratio
## 1 (0)-(2*(diagnosis... < 0 -0.03 0.02 -0.06 -0.01 65.39
## Post.Prob Star
## 1 0.98 *
## ---
## 'CI': 90%-CI for one-sided and 95%-CI for two-sided hypotheses.
## '*': For one-sided hypotheses, the posterior probability exceeds 95%;
## for two-sided hypotheses, the value tested against lies outside the 95%-CI.
## Posterior probabilities of point hypotheses assume equal prior probabilities.

# H1b: ADHD slower than COMP
h1b = hypothesis(m.rtsac,
 "0 < 2*diagnosis1 + diagnosis2 + diagnosis3")
h1b

## Hypothesis Tests for class b:
## Hypothesis Estimate Est.Error CI.Lower CI.Upper Evid.Ratio
## 1 (0)-(2*diagnosis1... < 0 -0.12 0.05 -0.2 -0.04 109.34
## Post.Prob Star
## 1 0.99 *
## ---
## 'CI': 90%-CI for one-sided and 95%-CI for two-sided hypotheses.
## '*': For one-sided hypotheses, the posterior probability exceeds 95%;
## for two-sided hypotheses, the value tested against lies outside the 95%-CI.
## Posterior probabilities of point hypotheses assume equal prior probabilities.

# H1c: ASD slower than COMP
h1c = hypothesis(m.rtsac,
 "0 < 2*diagnosis2 + diagnosis1 + diagnosis3")
h1c

## Hypothesis Tests for class b:
## Hypothesis Estimate Est.Error CI.Lower CI.Upper Evid.Ratio
## 1 (0)-(2*diagnosis2... < 0 -0.15 0.05 -0.23 -0.06 469.59
## Post.Prob Star
## 1 1 *
## ---
## 'CI': 90%-CI for one-sided and 95%-CI for two-sided hypotheses.
## '*': For one-sided hypotheses, the posterior probability exceeds 95%;
## for two-sided hypotheses, the value tested against lies outside the 95%-CI.
## Posterior probabilities of point hypotheses assume equal prior probabilities.

# H1d: FAB in ASD decreased compared to COMP
h1d = hypothesis(m.rtsac,
 "0 < 4*diagnosis2:cue1 + 2*diagnosis1:cue1 + 2*diagnosis3:cue1")
h1d

## Hypothesis Tests for class b:
## Hypothesis Estimate Est.Error CI.Lower CI.Upper Evid.Ratio
## 1 (0)-(4*diagnosis2... < 0 -0.02 0.01 -0.04 0.01 7.9
## Post.Prob Star
## 1 0.89
## ---
## 'CI': 90%-CI for one-sided and 95%-CI for two-sided hypotheses.
## '*': For one-sided hypotheses, the posterior probability exceeds 95%;
## for two-sided hypotheses, the value tested against lies outside the 95%-CI.
## Posterior probabilities of point hypotheses assume equal prior probabilities.

# H1e: FAB in ADHD differs from FAB in COMP (undirected)
h1e = hypothesis(m.rtsac,
 "0 > 4*diagnosis1:cue1 + 2*diagnosis2:cue1 + 2*diagnosis3:cue1",
 alpha = 0.025)
h1e

## Hypothesis Tests for class b:
## Hypothesis Estimate Est.Error CI.Lower CI.Upper Evid.Ratio
## 1 (0)-(4*diagnosis1... > 0 0.03 0.01 0 0.06 75.56
## Post.Prob Star
## 1 0.99 *
## ---
## 'CI': 95%-CI for one-sided and 97.5%-CI for two-sided hypotheses.
## '*': For one-sided hypotheses, the posterior probability exceeds 97.5%;
## for two-sided hypotheses, the value tested against lies outside the 97.5%-CI.
## Posterior probabilities of point hypotheses assume equal prior probabilities.

This model confirmed the same hypotheses.

# 4 Explorative analysis of subject-specific FAB

## 4.1 Overall RT, RADS and ASRS

# merge with the questionnaire values
df.que = df.fab.full %>%
 group_by(subID, diagnosis, stm, cue, ASRS_total, RAADS_total, adhd.meds, gender) %>%
 # summarise the median reaction time for each stimulus pair
 summarise(
 rt.cor = median(rt.cor, na.rm = T)
 ) %>%
 pivot_wider(names_from = cue, values_from = rt.cor) %>%
 # calculate the fab purely based on reaction times
 mutate(
 fab = object - face,
 overall = (object + face) / 2
 ) %>% group_by(subID, diagnosis, ASRS_total, RAADS_total, adhd.meds, gender) %>%
 # calculate the mean FAB per person
 summarise(
 fab = mean(fab),
 overall = mean(overall)
 ) %>% ungroup() %>%
 select(subID, diagnosis, overall, fab, ASRS_total, RAADS_total, adhd.meds, gender)

# check normal distributions
p1 = ggplot(df.que, aes(sample = ASRS_total)) +
 stat_qq(alpha = 0.75, colour = c_mid_highlight) +
 stat_qq_line() +
 theme_bw()
p2 = ggplot(df.que, aes(sample = RAADS_total)) +
 stat_qq(alpha = 0.75, colour = c_mid_highlight) +
 stat_qq_line() +
 theme_bw()
p3 = ggplot(df.que, aes(sample = fab)) +
 stat_qq(alpha = 0.75, colour = c_mid_highlight) +
 stat_qq_line() +
 theme_bw()
p4 = ggplot(df.que, aes(sample = overall)) +
 stat_qq(alpha = 0.75, colour = c_mid_highlight) +
 stat_qq_line() +
 theme_bw()
ggarrange(p1, p2, p3, p4,
 nrow = 2, ncol = 2, labels = "AUTO")


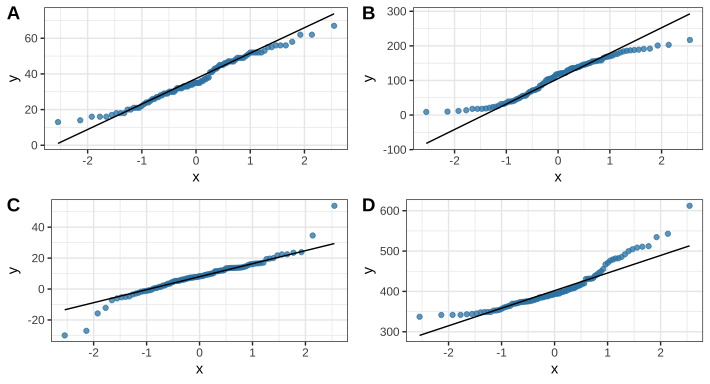


# do a Bayesian Spearman correlation: https://osf.io/j5wud
source("./helpers/rankBasedCommonFunctions.R")
source("./helpers/spearmanSampler.R")

# Default beta prior width is set to a = b = 1 for the sampler
if (file.exists("rho_ASRS.rds")) {
 rhoSamples.asrs = readRDS("rho_ASRS.rds")
} else {
 set.seed(5468)
 rhoSamples.asrs =
 spearmanGibbsSampler(xVals = df.que$ASRS_total,
 yVals = df.que$fab,
 nSamples = 5e3)
 saveRDS(rhoSamples.asrs, file = "rho_ASRS.rds")
}
if (file.exists("rho_RADS.rds")) {
 rhoSamples.rads = readRDS("rho_RADS.rds")
} else {
 set.seed(5468)
 rhoSamples.rads =
 spearmanGibbsSampler(xVals = df.que$RAADS_total,
 yVals = df.que$fab,
 nSamples = 5e3)
 saveRDS(rhoSamples.rads, file = "rho_RADS.rds")
}
if (file.exists("rho_RT.rds")) {
 rhoSamples.rt = readRDS("rho_RT.rds")
} else {
 set.seed(5478)
 rhoSamples.rt =
 spearmanGibbsSampler(xVals = df.que$overall,
 yVals = df.que$fab,
 nSamples = 5e3)
 saveRDS(rhoSamples.rt, file = "rho_RT.rds")
}

# give the posterior samples for rho to the function below to compute BF01
asrs.bf = computeBayesFactorOneZero(rhoSamples.asrs$rhoSamples,
 whichTest = "Spearman",
 priorParameter = 1)
rads.bf = computeBayesFactorOneZero(rhoSamples.rads$rhoSamples,
 whichTest = "Spearman",
 priorParameter = 1)
RT.bf = computeBayesFactorOneZero(rhoSamples.rt$rhoSamples,
 whichTest = "Spearman",
 priorParameter = 1)

Furthermore, Bayesian Spearman correlations revealed moderate evidence against associations between FAB and questionnaires assessing ASD (RADS: log(*BF*) = -1.72) or ADHD (ASRS: log(*BF*) = -1.57). Last, we explored whether FAB was associated with overall reaction times to assess whether attenuated FAB leads to better or worse task performance. A Bayesian Spearman correlation revealed moderate evidence against an association (log(*BF*) = -1.3).

# focus on the FAB effect
p = df.que %>%
 mutate(
 diagnosis = recode(diagnosis, "BOTH" = "ADHD+ASD"),
 diagnosis = factor(diagnosis, levels = c("ADHD", "ADHD+ASD", "ASD", "COMP")),
 diagnosis_med = as.factor(case_when(
 adhd.meds & diagnosis == "ADHD" ~ "ADHD medicated",
 adhd.meds & diagnosis == "ADHD+ASD" ~ "ADHD medicated+ASD",
 diagnosis == "ADHD" ~ "ADHD unmedicated",
 diagnosis == "ADHD+ASD" ~ "ADHD unmedicated+ASD",
 T ~ diagnosis
 ))
 ) %>%
 ggplot(aes(diagnosis, fab, fill = diagnosis_med, colour = diagnosis_med)) + #
 geom_rain(rain.side = 'r',
boxplot.args = list(color = "black", outlier.shape = NA, show_guide = FALSE, alpha = .8),
violin.args = list(color = "black", outlier.shape = NA, alpha = .8),
boxplot.args.pos = list(
 position = ggpp::position_dodgenudge(x = 0, width = 0.3), width = 0.3
),
point.args = list(show_guide = FALSE, alpha = .5),
violin.args.pos = list(
 width = 0.6, position = position_nudge(x = 0.16)),
point.args.pos = list(position = ggpp::position_dodgenudge(x = -0.25, width = 0.1))) +
 geom_hline(yintercept = 0) +
 scale_fill_manual(values = custom.col) +
 scale_color_manual(values = custom.col) +
 labs(title = "Face attention bias",
 x = "",
 y = "Difference (ms)") +
 theme_bw() +
 theme(legend.position = "inside", legend.position.inside = c(0.6, 0.8),
 plot.title = element_text(hjust = 0.5),
 legend.direction = "horizontal",
 legend.title=element_blank(), axis.title.y = element_text(size = 28),
 axis.text.x = element_text(size = 28, colour = "black"),
 axis.text.y = element_text(size = 24),
 text = element_text(family = "Merriweather", size = 32))

ggsave("Fig4_FAB.tif", plot = p,
 units = "mm",
 width = 170,
 height = 100,
 dpi = 300)

# focus on the FAB effect
df.que %>%
 mutate(
 diagnosis = recode(diagnosis, "BOTH" = "ADHD+ASD"),
 diagnosis = factor(diagnosis, levels = c("ADHD", "ADHD+ASD", "ASD", "COMP"))
 ) %>%
 ggplot(aes(diagnosis, fab, fill = diagnosis, colour = diagnosis)) + #
 geom_rain(rain.side = 'r',
boxplot.args = list(color = "black", outlier.shape = NA, show_guide = FALSE, alpha = .8),
violin.args = list(color = "black", outlier.shape = NA, alpha = .8),
boxplot.args.pos = list(
 position = ggpp::position_dodgenudge(x = 0, width = 0.3), width = 0.3
),
point.args = list(show_guide = FALSE, alpha = .5),
violin.args.pos = list(
 width = 0.6, position = position_nudge(x = 0.16)),
point.args.pos = list(position = ggpp::position_dodgenudge(x = -0.25, width = 0.1))) +
 geom_hline(yintercept = 0) +
 scale_fill_manual(values = custom.col[c(3, 4, 5, 6)]) +
 scale_color_manual(values = custom.col[c(3, 4, 5, 6)]) +
 labs(title = "Face attention bias",
 x = "",
 y = "Difference (ms)") +
 theme_bw() +
 theme(legend.position = "bottom",
 plot.title = element_text(hjust = 0.5),
 legend.direction = "horizontal",
 text = element_text(family = "Merriweather", size = 15),
 legend.title=element_blank())


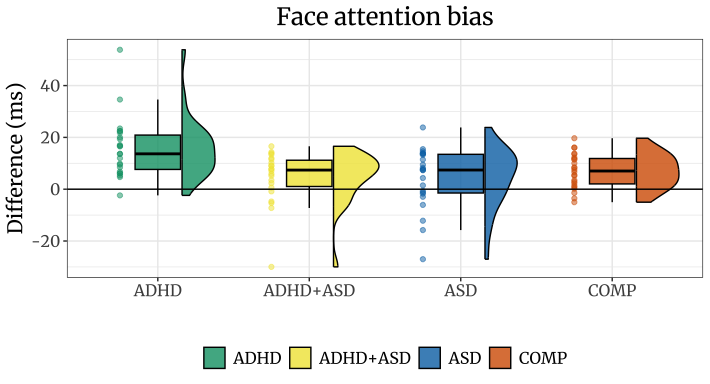


# plot the associations
df.que %>%
 pivot_longer(cols = c(ASRS_total, RAADS_total), names_to = "questionnaire") %>%
 ggplot(., aes(y = fab, x = value)) +
 geom_point(colour = c_mid_highlight, alpha = 0.75) +
 geom_smooth(method = "lm",
 formula = y ~ x,
 colour = c_dark_highlight) +
 facet_grid(. ~ questionnaire, scale = "free_x") +
 theme_bw()


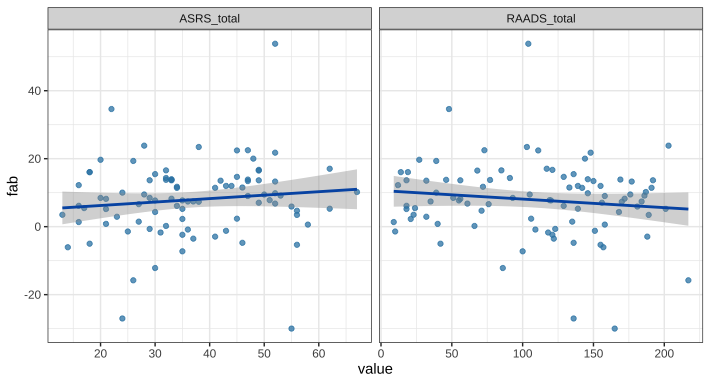


## 4.2 Gender and medication

df.que %>%
 mutate(fab = fab > 0) %>%
 group_by(diagnosis, gender, fab) %>% count()

## # A tibble: 16 × 4
## # Groups: diagnosis, gender, fab [16]
## diagnosis gender fab n
## <fct> <fct> <lgl> <int>
## 1 ADHD dan TRUE 2
## 2 ADHD fem TRUE 9
## 3 ADHD mal FALSE 1
## 4 ADHD mal TRUE 11
## 5 ASD fem FALSE 4
## 6 ASD fem TRUE 8
## 7 ASD mal FALSE 4
## 8 ASD mal TRUE 7
## 9 BOTH dan TRUE 3
## 10 BOTH fem FALSE 4
## 11 BOTH fem TRUE 8
## 12 BOTH mal FALSE 1
## 13 BOTH mal TRUE 6
## 14 COMP fem TRUE 11
## 15 COMP mal FALSE 3
## 16 COMP mal TRUE 10

set.seed(1234)
aov.gen = anovaBF(fab ~ gender * diagnosis, data = df.que %>% filter(gender != "dan"))
aov.gen@bayesFactor %>% mutate(interpretation = interpret_bf(bf, log = T))

## bf error
## diagnosis 2.8176916 4.331993e-05
## gender -1.0637247 1.768721e-04
## diagnosis + gender 1.5724113 1.081567e-02
## diagnosis + gender + diagnosis:gender 0.2966024 1.155677e-02
## time code
## diagnosis Fri Sep 26 11:27:43 2025 39b3fc67ee8
## gender Fri Sep 26 11:27:43 2025 39b330b5b96f
## diagnosis + gender Fri Sep 26 11:27:43 2025 39b348f4ed
## diagnosis + gender + diagnosis:gender Fri Sep 26 11:27:43 2025 39b35a53ddd5
## interpretation
## diagnosis strong evidence in favour of
## gender anecdotal evidence against
## diagnosis + gender moderate evidence in favour of
## diagnosis + gender + diagnosis:gender anecdotal evidence in favour of

df.que %>% filter(diagnosis %in% c("ADHD", "BOTH")) %>%
 mutate(fab = fab > 0) %>%
 group_by(adhd.meds, fab) %>% count()

## # A tibble: 4 × 3
## # Groups: adhd.meds, fab [4]
## adhd.meds fab n
## <lgl> <lgl> <int>
## 1 FALSE FALSE 3
## 2 FALSE TRUE 16
## 3 TRUE FALSE 3
## 4 TRUE TRUE 23

aov.med = anovaBF(fab ~ adhd.meds, data = df.que %>%
 filter(diagnosis %in% c("ADHD", "BOTH")) %>%
 mutate(adhd.meds = as.factor(adhd.meds)))
aov.med@bayesFactor %>% mutate(interpretation = interpret_bf(bf, log = T))

## bf error time code
## adhd.meds -1.210979 5.669869e-05 Fri Sep 26 11:27:43 2025 39b33808f947
## interpretation
## adhd.meds moderate evidence against

## 4.3 FAB as percentage of total reaction time

Since ADHD participants took longer on both cues, we want to make sure that the effect of FAB is actually increased and not just larger because they take longer to response.

df.fab.wide = df.fab %>%
 pivot_wider(names_from = cue, values_from = rt.cor) %>%
 mutate(fab.perc = (object - face)*100/(object + face))

# set weakly informed priors
priors = c(
 prior(normal(0, 0.50), class = Intercept),
 prior(normal(1.0, 1.00), class = sd),
 prior(lkj(2), class = cor),
 prior(normal(0, 1.00), class = b),
 prior(normal(0, 10.00), class = sigma)
)

m.fab.perc = brm(fab.perc ~ diagnosis + (1 | subID) + (diagnosis | stm) ,
 df.fab.wide, prior = priors,
 iter = iter, warmup = warm,
 backend = "cmdstanr", threads = threading(8),
 file = "m_fab_perc",
 seed = 222
 )
rstan::check_hmc_diagnostics(m.fab.perc$fit)

##
## Divergences:

##
## Tree depth:

##
## Energy:

# check that rhats are below 1.01
sum(brms::rhat(m.fab.perc) >= 1.01, na.rm = T)

## [1] 0

# print the summary
summary(m.fab.perc)

## Family: gaussian
## Links: mu = identity; sigma = identity
## Formula: fab.perc ~ diagnosis + (1 | subID) + (diagnosis | stm)
## Data: df.fab.wide (Number of observations: 3312)
## Draws: 4 chains, each with iter = 3000; warmup = 1000; thin = 1;
## total post-warmup draws = 8000
##
## Multilevel Hyperparameters:
## ~stm (Number of levels: 36)
## Estimate Est.Error l-95% CI u-95% CI Rhat Bulk_ESS
## sd(Intercept) 1.76 0.23 1.36 2.26 1.00 2060
## sd(diagnosis1) 0.31 0.21 0.01 0.77 1.00 2613
## sd(diagnosis2) 0.23 0.17 0.01 0.61 1.00 3534
## sd(diagnosis3) 0.28 0.19 0.01 0.70 1.00 2822
## cor(Intercept,diagnosis1) -0.01 0.33 -0.65 0.62 1.00 10018
## cor(Intercept,diagnosis2) 0.02 0.35 -0.66 0.67 1.00 9300
## cor(diagnosis1,diagnosis2) -0.13 0.39 -0.79 0.65 1.00 6174
## cor(Intercept,diagnosis3) -0.15 0.34 -0.73 0.55 1.00 10128
## cor(diagnosis1,diagnosis3) -0.18 0.39 -0.82 0.61 1.00 5590
## cor(diagnosis2,diagnosis3) -0.04 0.38 -0.72 0.69 1.00 6798
## Tail_ESS
## sd(Intercept) 3624
## sd(diagnosis1) 3600
## sd(diagnosis2) 4671
## sd(diagnosis3) 3202
## cor(Intercept,diagnosis1) 5916
## cor(Intercept,diagnosis2) 5607
## cor(diagnosis1,diagnosis2) 5105
## cor(Intercept,diagnosis3) 5843
## cor(diagnosis1,diagnosis3) 5480
## cor(diagnosis2,diagnosis3) 6634
##
## ~subID (Number of levels: 92)
## Estimate Est.Error l-95% CI u-95% CI Rhat Bulk_ESS Tail_ESS
## sd(Intercept) 0.82 0.13 0.57 1.09 1.00 3053 4447
##
## Regression Coefficients:
## Estimate Est.Error l-95% CI u-95% CI Rhat Bulk_ESS Tail_ESS
## Intercept 0.67 0.27 0.13 1.20 1.00 1793 3242
## diagnosis1 0.80 0.21 0.36 1.22 1.00 4831 5036
## diagnosis2 -0.42 0.21 -0.83 0.01 1.00 5698 4716
## diagnosis3 -0.31 0.22 -0.74 0.11 1.00 4893 4773
##
## Further Distributional Parameters:
## Estimate Est.Error l-95% CI u-95% CI Rhat Bulk_ESS Tail_ESS
## sigma 5.09 0.07 4.97 5.23 1.00 10076 5423
##
## Draws were sampled using sample(hmc). For each parameter, Bulk_ESS
## and Tail_ESS are effective sample size measures, and Rhat is the potential
## scale reduction factor on split chains (at convergence, Rhat = 1).

# FAB in ADHD > FAB in COMP
hypothesis(m.fab.perc, "diagnosis1 > - diagnosis1 - diagnosis2 - diagnosis3")

## Hypothesis Tests for class b:
## Hypothesis Estimate Est.Error CI.Lower CI.Upper Evid.Ratio
## 1 (diagnosis1)-(-di... > 0 0.87 0.36 0.28 1.46 124
## Post.Prob Star
## 1 0.99 *
## ---
## 'CI': 90%-CI for one-sided and 95%-CI for two-sided hypotheses.
## '*': For one-sided hypotheses, the posterior probability exceeds 95%;
## for two-sided hypotheses, the value tested against lies outside the 95%-CI.
## Posterior probabilities of point hypotheses assume equal prior probabilities.

# print an overview
kable(df.fab.wide %>% group_by(diagnosis, subID) %>%
 summarise(fab.perc = mean(fab.perc)) %>%
 group_by(diagnosis) %>%
 summarise(
 fab.perc.avg = mean(fab.perc),
 fab.perc.se = sd(fab.perc)/sqrt(n())
 ) %>% arrange(desc(fab.perc.avg)))

| diagnosis | fab.perc.avg | fab.perc.se |
| --- | --- | --- |
| ADHD | 1.8046277 | 0.2552834 |
| COMP | 0.8870717 | 0.1772152 |
| BOTH | 0.6075702 | 0.2310650 |
| ASD | 0.5096168 | 0.3028107 |

# 5 Explorative analysis of errors

Last but not least, we are going to explore possible differences with regards to mean accuracies using a bernoulli distribution.

Next, we are going to explore possible differences with regards to accuracy. We use a bernoulli distribution to model the threshold between correct and incorrect trials. We computed the SBC outside of this script in batches to avoid running out of memory. Then, we combined it and load the results in here.

## 5.1 Simulation-based calibration

# figure out slopes for subject
kable(head(df.fab.full %>% count(subID, cue)))

| subID | cue | n |
| --- | --- | --- |
| 1 | face | 216 |
| 1 | object | 216 |
| 10 | face | 216 |
| 10 | object | 216 |
| 11 | face | 216 |
| 11 | object | 216 |

kable(head(df.fab.full %>% count(stm, cue, diagnosis)))

| stm | cue | diagnosis | n |
| --- | --- | --- | --- |
| 1_10 | face | ADHD | 138 |
| 1_10 | face | ASD | 138 |
| 1_10 | face | BOTH | 132 |
| 1_10 | face | COMP | 144 |
| 1_10 | object | ADHD | 138 |
| 1_10 | object | ASD | 138 |

code = "FAB_err"

# increase iterations a bit to improve rhats
iter = 4000
warm = 2000

# code accuracy to track errors
df.fab.full = df.fab.full %>%
 mutate(
 error = if_else(acc,0,1)
 )

# set the formula
f.err = brms::bf(error ~ diagnosis * cue + (cue | subID) + (diagnosis * cue | stm) )

# set weakly informed priors
priors = c(
 prior(normal(6.0, 1.00), class = Intercept),
 prior(normal(1.0, 0.50), class = sd),
 prior(lkj(2), class = cor),
 # no specific expectations for the rest of the effects
 prior(normal(0, 1.00), class = b)
)

# check if the SBC already exists
if (file.exists(file.path(cache_dir, sprintf("df_res_%s.rds", code)))) {
 # load in the resultsn of the SBC
 df.results = readRDS(file.path(cache_dir, sprintf("df_res_%s.rds", code)))
 df.backend = readRDS(file.path(cache_dir, sprintf("df_div_%s.rds", code)))
 dat = readRDS(file.path(cache_dir, sprintf("dat_%s.rds", code)))
} else {
 # perform SBC
 gen = SBC_generator_brms(f.err, data = df.fab.full, prior = priors,
 thin = 50, warmup = 10000, refresh = 2000,
 generate_lp = TRUE, family = bernoulli, init = 0.1)
 bck = SBC_backend_brms_from_generator(gen, chains = 4, thin = 1,
 warmup = warm, iter = iter)
 if (!file.exists(file.path(cache_dir, sprintf("dat_%s.rds", code)))) {
 dat = generate_datasets(gen, nsim)
 saveRDS(dat, file.path(cache_dir, sprintf("dat_%s.rds", code)))
 } else {
 dat = readRDS(file.path(cache_dir, sprintf("dat_%s.rds", code)))
 }
 res = compute_SBC(dat,
 bck,
 cache_mode = "results",
 cache_location = file.path(cache_dir, sprintf("res_%s", code)))
 df.results = res$stats
 df.backend = res$backend_diagnostics
 saveRDS(df.results, file = file.path(cache_dir, paste0("df_res_", code, ".rds")))
 saveRDS(df.backend, file = file.path(cache_dir, paste0("df_div_", code, ".rds")))
}

Looking at the rhats and divergent transitions shows that 0 of 250 simulations had at least one parameter that had an rhat of at least 1.05 and 2 had divergent samples. Therefore, we continue with this model and plot the simulated values to perform prior predictive checks.

# create a matrix out of generated data
dvname = gsub(" ", "", gsub("[\\|~].*", "", f.err)[1])
dvfakemat = matrix(NA, nrow(dat[['generated']][[1]]), length(dat[['generated']]))
for (i in 1:length(dat[['generated']])) {
 dvfakemat[,i] = dat[['generated']][[i]][[dvname]]
}
truePars = dat$variables

# compute one histogram per simulated data-set
options = c(0, 1)
histmat = matrix(NA, ncol = nrow(truePars), length(options))
for (i in 1:nrow(truePars)) {
 for (j in 1:length(options))
 {
 histmat[j,i] = sum(dvfakemat[,i] == options[j])
 }
}
# for each bin, compute quantiles across histograms
probs = seq(0.1, 0.9, 0.1)
quantmat= as.data.frame(matrix(NA, nrow=dim(histmat)[1], ncol = length(probs)))
names(quantmat) = paste0("p", probs)
for (i in 1:dim(histmat)[1]) {
 quantmat[i,] = quantile(histmat[i,], p = probs)
}
quantmat$x = c("error", "correct")
p0 = ggplot(data = quantmat, aes(x = x)) +
 geom_bar(aes(y = p0.9), fill = c_light, stat = "identity") +
 geom_bar(aes(y = p0.8), fill = c_light_highlight, stat = "identity") +
 geom_bar(aes(y = p0.7), fill = c_mid, stat = "identity") +
 geom_bar(aes(y = p0.6), fill = c_mid_highlight, stat = "identity") +
 geom_bar(aes(y = p0.5), fill = c_dark, stat = "identity") +
 labs(title = "Prior predictive distribution", y = "", x = "") +
 theme_bw()

# get simulation numbers with issues
check = merge(df.results %>%
 group_by(sim_id) %>% summarise(rhat = max(rhat, na.rm = T), max_rank = min(max_rank)) %>%
 filter(rhat >= 1.05 | max_rank != max(max_rank)),
 df.backend %>% filter(n_divergent > 0), all = T)

# plot SBC with functions from the SBC package focusing on population-level parameters
df.results.b = df.results %>%
 filter(substr(variable, 1, 2) == "b_") %>%
 filter(!(sim_id %in% check$sim_id))
p1 = plot_ecdf_diff(df.results.b) + theme_bw() + theme(legend.position = "none") +
 scale_x_continuous(breaks=scales::pretty_breaks(n = 3)) +
 scale_y_continuous(breaks=scales::pretty_breaks(n = 3))
p2 = plot_rank_hist(df.results.b, bins = 20) + theme_bw() +
 scale_x_continuous(breaks=scales::pretty_breaks(n = 3)) +
 scale_y_continuous(breaks=scales::pretty_breaks(n = 3))
p3 = plot_sim_estimated(df.results.b, alpha = 0.5) + theme_bw() +
 scale_x_continuous(breaks=scales::pretty_breaks(n = 3)) +
 scale_y_continuous(breaks=scales::pretty_breaks(n = 3))

prior_sd = setNames(rep(1, length(unique(df.results.b$variable))), # all same SD
 unique(df.results.b$variable))

p4 = plot_contraction(df.results.b, prior_sd = prior_sd) + theme_bw() +
 scale_x_continuous(breaks=scales::pretty_breaks(n = 3))

p = ggarrange(p0, p1, p2, p3, p4,
 labels = "AUTO", ncol = 1, nrow = 5,
 heights = c(1, 2, 2, 2, 2))
annotate_figure(p,
 top = text_grob("Prior predictive checks and SBC",
 face = "bold", size = 14))

Everything looks good with the wider priors, so we continue and run the model.

## 5.2 Posterior predictive checks

As the next step, we fit the model, check whether there are divergence or rhat issues, and then check whether the chains have converged.

# fit the final model
set.seed(1234)
m.err = brm(f.err,
 df.fab.full, prior = priors,
 iter = iter, warmup = warm,
 family = "bernoulli",
 backend = "cmdstanr", threads = threading(8),
 file = "m_err",
 save_pars = save_pars(all = TRUE)
 )
rstan::check_hmc_diagnostics(m.err$fit)

##
## Divergences:

## 0 of 8000 iterations ended with a divergence.

##
## Tree depth:

## 0 of 8000 iterations saturated the maximum tree depth of 10.

##
## Energy:

## E-BFMI indicated no pathological behavior.

# check that rhats are below 1.01
sum(brms::rhat(m.err) >= 1.01, na.rm = T)

## [1] 0

# check the trace plots
post.draws = as_draws_df(m.err)
mcmc_trace(post.draws, regex_pars = "^b_",
 facet_args = list(ncol = 4)) +
 scale_x_continuous(breaks=scales::pretty_breaks(n = 3)) +
 scale_y_continuous(breaks=scales::pretty_breaks(n = 3))

## Scale for x is already present.
## Adding another scale for x, which will replace the existing scale.


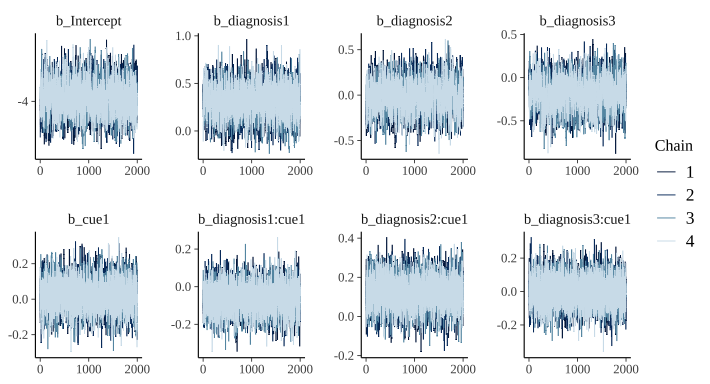


Again, we use the function brms::pp_check() with 250 draws to check whether the predicted data resembles the actual data as well as the ppc_stat_grouped function from the bayesplot package to check posterior fit for each diagnostic group separately. The model seems to be a good fit with the predicted data closely mirroring the real data.

# get posterior predictions
post.pred = posterior_predict(m.err, ndraws = nsim)

# check the fit of the predicted data compared to the real data
p1 = pp_check(m.err, ndraws = nsim, type = "bars") +
 theme_bw() + theme(legend.position = "none") + labs(y = "")

# distributions of means compared to the real values per group
p2 = ppc_stat_grouped(df.fab.full$error, post.pred, df.fab.full$diagnosis) +
 theme_bw() + theme(legend.position = "none")

p = ggarrange(p1, p2,
 nrow = 2, ncol = 1, labels = "AUTO")
annotate_figure(p,
 top = text_grob("Posterior predictive checks: errors",
 face = "bold", size = 14))


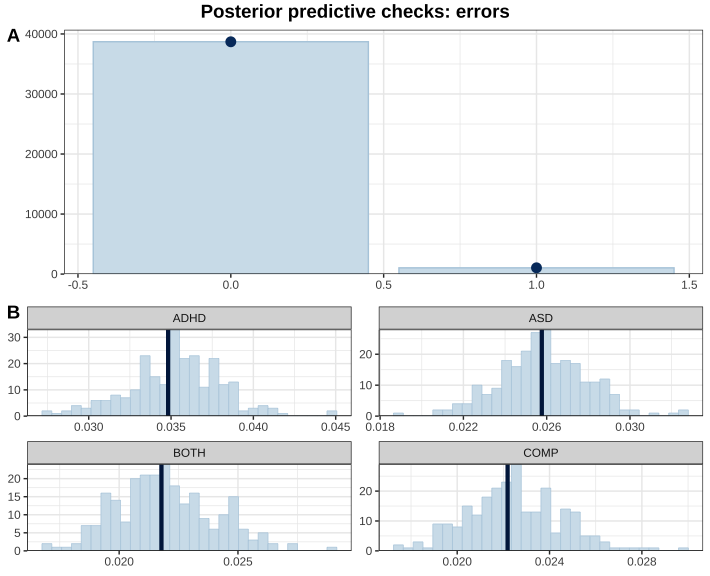


Our model fits the data very well.

## 5.3 Inferences

Now that we are convinced that we can trust our model, we have a look at its estimate and use the hypothesis function to perform explorative tests.

# print a summary
summary(m.err)

## Family: bernoulli
## Links: mu = logit
## Formula: error ~ diagnosis * cue + (cue | subID) + (diagnosis * cue | stm)
## Data: df.fab.full (Number of observations: 39744)
## Draws: 4 chains, each with iter = 4000; warmup = 2000; thin = 1;
## total post-warmup draws = 8000
##
## Multilevel Hyperparameters:
## ~stm (Number of levels: 36)
## Estimate Est.Error l-95% CI u-95% CI Rhat
## sd(Intercept) 0.27 0.06 0.17 0.40 1.00
## sd(diagnosis1) 0.10 0.06 0.00 0.23 1.00
## sd(diagnosis2) 0.08 0.06 0.00 0.21 1.00
## sd(diagnosis3) 0.10 0.07 0.00 0.26 1.00
## sd(cue1) 0.41 0.07 0.29 0.56 1.00
## sd(diagnosis1:cue1) 0.10 0.07 0.00 0.24 1.00
## sd(diagnosis2:cue1) 0.10 0.07 0.01 0.25 1.00
## sd(diagnosis3:cue1) 0.20 0.09 0.03 0.38 1.00
## cor(Intercept,diagnosis1) -0.09 0.28 -0.61 0.49 1.00
## cor(Intercept,diagnosis2) 0.10 0.29 -0.49 0.64 1.00
## cor(diagnosis1,diagnosis2) -0.03 0.30 -0.60 0.55 1.00
## cor(Intercept,diagnosis3) 0.07 0.29 -0.51 0.60 1.00
## cor(diagnosis1,diagnosis3) -0.01 0.30 -0.57 0.55 1.00
## cor(diagnosis2,diagnosis3) -0.05 0.31 -0.62 0.55 1.00
## cor(Intercept,cue1) -0.15 0.20 -0.53 0.26 1.00
## cor(diagnosis1,cue1) 0.09 0.28 -0.48 0.60 1.01
## cor(diagnosis2,cue1) 0.10 0.30 -0.50 0.64 1.00
## cor(diagnosis3,cue1) -0.01 0.28 -0.55 0.54 1.00
## cor(Intercept,diagnosis1:cue1) -0.08 0.30 -0.62 0.51 1.00
## cor(diagnosis1,diagnosis1:cue1) 0.01 0.31 -0.58 0.60 1.00
## cor(diagnosis2,diagnosis1:cue1) -0.00 0.30 -0.58 0.57 1.00
## cor(diagnosis3,diagnosis1:cue1) -0.03 0.30 -0.61 0.55 1.00
## cor(cue1,diagnosis1:cue1) -0.01 0.29 -0.56 0.54 1.00
## cor(Intercept,diagnosis2:cue1) -0.12 0.29 -0.65 0.46 1.00
## cor(diagnosis1,diagnosis2:cue1) 0.07 0.30 -0.52 0.63 1.00
## cor(diagnosis2,diagnosis2:cue1) -0.03 0.30 -0.60 0.56 1.00
## cor(diagnosis3,diagnosis2:cue1) 0.06 0.30 -0.54 0.63 1.00
## cor(cue1,diagnosis2:cue1) 0.04 0.28 -0.52 0.57 1.00
## cor(diagnosis1:cue1,diagnosis2:cue1) -0.06 0.30 -0.62 0.53 1.00
## cor(Intercept,diagnosis3:cue1) -0.18 0.26 -0.65 0.35 1.00
## cor(diagnosis1,diagnosis3:cue1) -0.03 0.29 -0.58 0.54 1.00
## cor(diagnosis2,diagnosis3:cue1) -0.01 0.30 -0.58 0.57 1.00
## cor(diagnosis3,diagnosis3:cue1) 0.01 0.30 -0.56 0.59 1.00
## cor(cue1,diagnosis3:cue1) 0.14 0.24 -0.36 0.59 1.00
## cor(diagnosis1:cue1,diagnosis3:cue1) 0.02 0.29 -0.54 0.58 1.00
## cor(diagnosis2:cue1,diagnosis3:cue1) 0.00 0.29 -0.55 0.57 1.00
## Bulk_ESS Tail_ESS
## sd(Intercept) 3180 4943
## sd(diagnosis1) 3037 3160
## sd(diagnosis2) 4164 3892
## sd(diagnosis3) 2639 3595
## sd(cue1) 4461 5351
## sd(diagnosis1:cue1) 2894 3630
## sd(diagnosis2:cue1) 3559 3888
## sd(diagnosis3:cue1) 3044 2652
## cor(Intercept,diagnosis1) 11464 6131
## cor(Intercept,diagnosis2) 12676 5841
## cor(diagnosis1,diagnosis2) 10568 5984
## cor(Intercept,diagnosis3) 10830 5808
## cor(diagnosis1,diagnosis3) 9519 6325
## cor(diagnosis2,diagnosis3) 7752 6125
## cor(Intercept,cue1) 3418 4852
## cor(diagnosis1,cue1) 1314 2330
## cor(diagnosis2,cue1) 1175 2265
## cor(diagnosis3,cue1) 1698 3942
## cor(Intercept,diagnosis1:cue1) 11169 6189
## cor(diagnosis1,diagnosis1:cue1) 8951 6180
## cor(diagnosis2,diagnosis1:cue1) 7464 6489
## cor(diagnosis3,diagnosis1:cue1) 6554 6153
## cor(cue1,diagnosis1:cue1) 11797 6838
## cor(Intercept,diagnosis2:cue1) 11154 5917
## cor(diagnosis1,diagnosis2:cue1) 8485 6081
## cor(diagnosis2,diagnosis2:cue1) 7835 6775
## cor(diagnosis3,diagnosis2:cue1) 6929 6569
## cor(cue1,diagnosis2:cue1) 11365 6347
## cor(diagnosis1:cue1,diagnosis2:cue1) 5844 5975
## cor(Intercept,diagnosis3:cue1) 8037 6164
## cor(diagnosis1,diagnosis3:cue1) 5547 5893
## cor(diagnosis2,diagnosis3:cue1) 4725 5764
## cor(diagnosis3,diagnosis3:cue1) 5023 5485
## cor(cue1,diagnosis3:cue1) 8883 6422
## cor(diagnosis1:cue1,diagnosis3:cue1) 6098 6517
## cor(diagnosis2:cue1,diagnosis3:cue1) 5441 6221
##
## ~subID (Number of levels: 92)
## Estimate Est.Error l-95% CI u-95% CI Rhat Bulk_ESS Tail_ESS
## sd(Intercept) 0.86 0.09 0.71 1.05 1.00 2188 3544
## sd(cue1) 0.21 0.07 0.05 0.33 1.00 1189 635
## cor(Intercept,cue1) 0.11 0.23 -0.37 0.54 1.00 5792 4821
##
## Regression Coefficients:
## Estimate Est.Error l-95% CI u-95% CI Rhat Bulk_ESS Tail_ESS
## Intercept -3.97 0.11 -4.19 -3.74 1.00 2326 3451
## diagnosis1 0.30 0.16 -0.02 0.63 1.00 2064 3624
## diagnosis2 -0.01 0.17 -0.34 0.33 1.00 2008 3333
## diagnosis3 -0.18 0.17 -0.53 0.16 1.00 2022 3971
## cue1 0.03 0.08 -0.14 0.19 1.00 4801 5423
## diagnosis1:cue1 -0.07 0.07 -0.21 0.08 1.00 7398 6505
## diagnosis2:cue1 0.12 0.08 -0.03 0.27 1.00 6430 6252
## diagnosis3:cue1 -0.00 0.09 -0.16 0.17 1.00 6506 5913
##
## Draws were sampled using sample(hmc). For each parameter, Bulk_ESS
## and Tail_ESS are effective sample size measures, and Rhat is the potential
## scale reduction factor on split chains (at convergence, Rhat = 1).

# plot the posterior distributions
as_draws_df(m.err) %>%
 select(starts_with("b_")) %>%
 mutate(
 b_COMP = - b_diagnosis1 - b_diagnosis2 - b_diagnosis3
 ) %>%
 pivot_longer(cols = starts_with("b_"), names_to = "coef", values_to = "estimate") %>%
 subset(!startsWith(coef, "b_Int")) %>%
 mutate(
 coef = substr(coef, 3, nchar(coef)),
 coef = str_replace_all(coef, ":", " x "),
 coef = str_replace_all(coef, "diagnosis1", "ADHD"),
 coef = str_replace_all(coef, "diagnosis2", "ASD"),
 coef = str_replace_all(coef, "diagnosis3", "ADHD+ASD"),
 coef = str_replace_all(coef, "cue1", "Face"),
 coef = fct_reorder(coef, desc(estimate))
 ) %>%
 group_by(coef) %>%
 mutate(
 cred = case_when(
 (mean(estimate) < 0 & quantile(estimate, probs = 0.975) < 0) |
 (mean(estimate) > 0 & quantile(estimate, probs = 0.025) > 0) ~ "credible",
 T ~ "not credible"
 )
 ) %>% ungroup() %>%
 ggplot(aes(x = estimate, y = coef, fill = cred)) +
 geom_vline(xintercept = 0, linetype = 'dashed') +
 ggdist::stat_halfeye(alpha = 0.7) + ylab(NULL) + theme_bw() +
 scale_fill_manual(values = c(c_light, c_dark)) + theme(legend.position = "none")


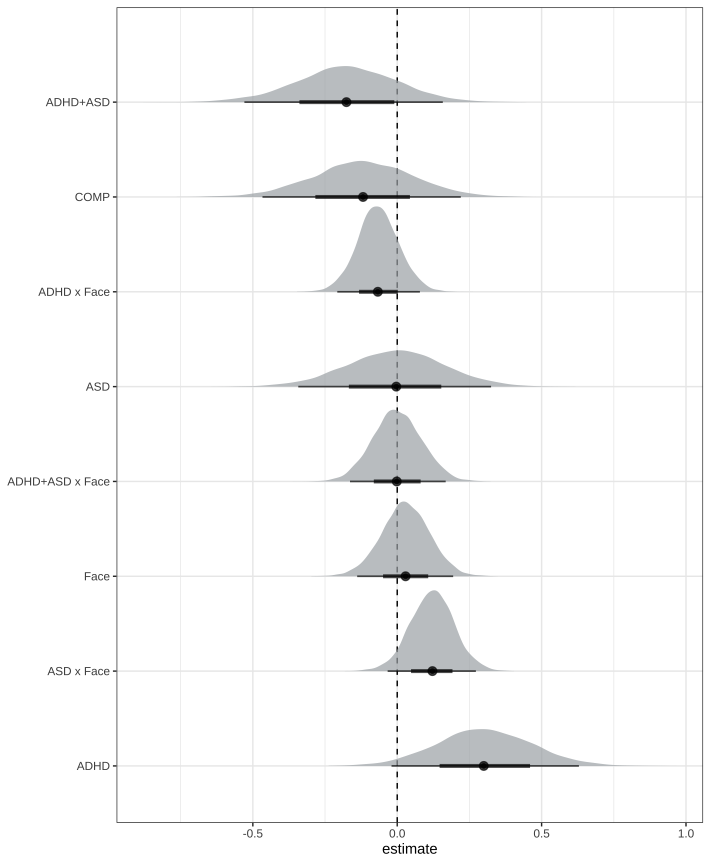


# COMP < ADHD
e1 = hypothesis(m.err, "0 < 2*diagnosis1 + diagnosis2 + diagnosis3", alpha = 0.025)
e1

## Hypothesis Tests for class b:
## Hypothesis Estimate Est.Error CI.Lower CI.Upper Evid.Ratio
## 1 (0)-(2*diagnosis1... < 0 -0.42 0.27 -0.96 0.11 15.7
## Post.Prob Star
## 1 0.94
## ---
## 'CI': 95%-CI for one-sided and 97.5%-CI for two-sided hypotheses.
## '*': For one-sided hypotheses, the posterior probability exceeds 97.5%;
## for two-sided hypotheses, the value tested against lies outside the 97.5%-CI.
## Posterior probabilities of point hypotheses assume equal prior probabilities.

# COMP < ASD
e2 = hypothesis(m.err, "0 < 2*diagnosis2 + diagnosis1 + diagnosis3", alpha = 0.025)
e2

## Hypothesis Tests for class b:
## Hypothesis Estimate Est.Error CI.Lower CI.Upper Evid.Ratio
## 1 (0)-(2*diagnosis2... < 0 -0.11 0.28 -0.68 0.44 1.92
## Post.Prob Star
## 1 0.66
## ---
## 'CI': 95%-CI for one-sided and 97.5%-CI for two-sided hypotheses.
## '*': For one-sided hypotheses, the posterior probability exceeds 97.5%;
## for two-sided hypotheses, the value tested against lies outside the 97.5%-CI.
## Posterior probabilities of point hypotheses assume equal prior probabilities.

# COMP < BOTH
e3 = hypothesis(m.err, "0 > 2*diagnosis3 + diagnosis1 + diagnosis2", alpha = 0.025)
e3

## Hypothesis Tests for class b:
## Hypothesis Estimate Est.Error CI.Lower CI.Upper Evid.Ratio
## 1 (0)-(2*diagnosis3... > 0 0.06 0.29 -0.5 0.62 1.42
## Post.Prob Star
## 1 0.59
## ---
## 'CI': 95%-CI for one-sided and 97.5%-CI for two-sided hypotheses.
## '*': For one-sided hypotheses, the posterior probability exceeds 97.5%;
## for two-sided hypotheses, the value tested against lies outside the 97.5%-CI.
## Posterior probabilities of point hypotheses assume equal prior probabilities.

# explore differences between cues
e4 = hypothesis(m.err, "0 < 2*cue1", alpha = 0.025)
e4

## Hypothesis Tests for class b:
## Hypothesis Estimate Est.Error CI.Lower CI.Upper Evid.Ratio Post.Prob
## 1 (0)-(2*cue1) < 0 -0.06 0.17 -0.39 0.28 1.79 0.64
## Star
## 1
## ---
## 'CI': 95%-CI for one-sided and 97.5%-CI for two-sided hypotheses.
## '*': For one-sided hypotheses, the posterior probability exceeds 97.5%;
## for two-sided hypotheses, the value tested against lies outside the 97.5%-CI.
## Posterior probabilities of point hypotheses assume equal prior probabilities.

# extract predicted differences
df.new = df.fab.full %>%
 select(diagnosis, cue) %>%
 mutate(
 condition = paste0(diagnosis, '_', cue)
 ) %>%
 distinct()
df.ms = as.data.frame(
 fitted(m.err, summary = F,
 newdata = df.new,
 re_formula = NA))
colnames(df.ms) = df.new$condition

st(df.ms,
 summ = c('mean(x)','sd(x)','min(x)','pctile(x)[2.5]',
 'pctile(x)[97.5]','max(x)'))

Summary Statistics

| Variable | Mean | Sd | Min | Pctile[2.5] | Pctile[97.5] | Max |
| --- | --- | --- | --- | --- | --- | --- |
| ADHD_face | 0.025 | 0.0054 | 0.011 | 0.015 | 0.036 | 0.05 |
| ADHD_object | 0.026 | 0.0057 | 0.012 | 0.016 | 0.039 | 0.057 |
| COMP_face | 0.017 | 0.0039 | 0.0064 | 0.0098 | 0.025 | 0.037 |
| COMP_object | 0.017 | 0.004 | 0.0065 | 0.01 | 0.026 | 0.038 |
| ASD_face | 0.022 | 0.0049 | 0.008 | 0.013 | 0.032 | 0.05 |
| ASD_object | 0.016 | 0.0037 | 0.0066 | 0.0098 | 0.024 | 0.037 |
| BOTH_face | 0.016 | 0.004 | 0.0061 | 0.0095 | 0.025 | 0.04 |
| BOTH_object | 0.016 | 0.0039 | 0.0056 | 0.009 | 0.024 | 0.036 |

st(df.ms %>%
 mutate(
 face = rowMeans(select(., matches(".*_face")), na.rm = T),
 object = rowMeans(select(., matches(".*_object")), na.rm = T),
 FAB = object - face
 ) %>% select(face, object, FAB),
 summ = c('mean(x)','sd(x)','min(x)','pctile(x)[2.5]','pctile(x)[97.5]','max(x)'))

Summary Statistics

| Variable | Mean | Sd | Min | Pctile[2.5] | Pctile[97.5] | Max |
| --- | --- | --- | --- | --- | --- | --- |
| face | 0.02 | 0.0027 | 0.012 | 0.015 | 0.025 | 0.032 |
| object | 0.019 | 0.0027 | 0.01 | 0.014 | 0.024 | 0.032 |
| FAB | -0.00094 | 0.0032 | -0.014 | -0.0073 | 0.0052 | 0.013 |

Accuracies were generally high, with a total of 2.61% inaccurate responses across diagnostic groups. The explorative analysis of the error rates revealed no credible differences between any of the diagnostic groups and no credible difference between cues (see supplementary materials).

## 5.4 Plots

# overall accuracies
df.fab.full %>%
 group_by(subID, diagnosis, cue) %>%
 summarise(error = 100*mean(error, na.rm = T)) %>%
 ggplot(aes(diagnosis, error, fill = cue, colour = cue)) + #
 geom_rain(rain.side = 'r',
boxplot.args = list(color = "black", outlier.shape = NA, show_guide = FALSE, alpha = .8),
violin.args = list(color = "black", outlier.shape = NA, alpha = .8),
boxplot.args.pos = list(
 position = ggpp::position_dodgenudge(x = 0, width = 0.3), width = 0.3
),
point.args = list(show_guide = FALSE, alpha = .5),
violin.args.pos = list(
 width = 0.6, position = position_nudge(x = 0.16)),
point.args.pos = list(position = ggpp::position_dodgenudge(x = -0.25, width = 0.1))) +
 ylim(0, 25) +
 scale_fill_manual(values = custom.col2) +
 scale_color_manual(values = custom.col2) +
 labs(title = "Mean error rates", x = "", y = "percent") +
 theme_bw() +
 theme(legend.position = "bottom",
 plot.title = element_text(hjust = 0.5),
 legend.direction = "horizontal",
 text = element_text(family = "Merriweather", size = 15))


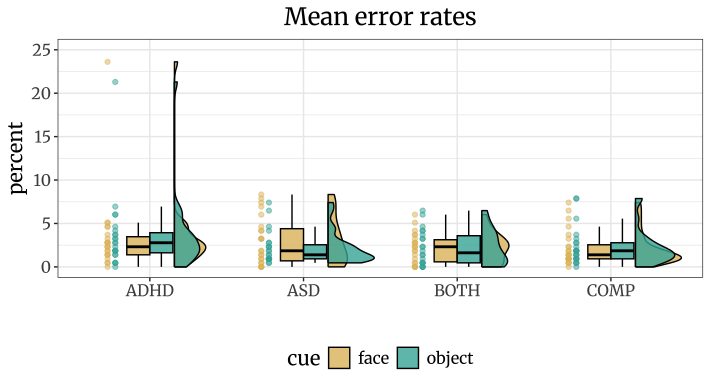


# 6 Summary table

# get grand average of accuracies and reaction times
df.agg = rbind(
 df.fab.full %>%
 group_by(subID, diagnosis, cue) %>%
 summarise(error = 100*mean(error, na.rm = T)) %>%
 group_by(diagnosis, cue) %>%
 summarise(mean = mean(error, na.rm = T), se = sd(error, na.rm = T)/sqrt(n())) %>%
 mutate(measure = "accuracy") %>%
 mutate(
 value = sprintf("%.2f ±%.2f", mean, se)
 ) %>% select(measure, diagnosis, cue, value) %>%
 pivot_wider(names_from = c(diagnosis, cue), values_from = value),
 df.fab.full %>%
 group_by(subID, diagnosis, cue) %>%
 summarise(rt.cor = mean(rt.cor, na.rm = T)) %>%
 group_by(diagnosis, cue) %>%
 summarise(mean = mean(rt.cor, na.rm = T), se = sd(rt.cor, na.rm = T)/sqrt(n())) %>%
 mutate(measure = "rt.cor") %>%
 mutate(
 value = sprintf("%.0f ±%.0f", mean, se)
 ) %>% select(measure, diagnosis, cue, value) %>%
 pivot_wider(names_from = c(diagnosis, cue), values_from = value))

read_docx() %>%
 body_add_table(df.agg) %>%
 print(target = "FAB_tbl2.docx")

# 7 Preparation for eye-tracking analysis

First, we reload the data. Second, we preprocess the latencies of the saccades and divide them into saccades elicited by the cues and saccades elicited by the target. To do so, we use the knowledge that latencies below 100ms are extremely unlikely and use the global minimum in the density function.

# number of simulations
nsim = 250

# set the seed
set.seed(2468)

# load the data
load("FAB_data.RData")

# combine both behavioural datasets
df.fab = rbind(df.fab, df.exp)
df.fab$diagnosis = factor(df.fab$diagnosis,
 levels = c("ADHD", "ASD", "BOTH", "COMP"))

# compute subject specific FAB
df.fab.agg = df.fab %>%
 group_by(subID, diagnosis, stm, cue) %>%
 # summarise the median reaction time for each stimulus pair
 summarise(
 rt.cor = median(rt.cor, na.rm = T)
 ) %>%
 pivot_wider(names_from = cue, values_from = rt.cor) %>%
 # calculate the fab purely based on reaction times
 mutate(
 fab = object - face
 ) %>% group_by(subID, diagnosis) %>%
 # calculate the mean FAB per person
 summarise(
 fab = mean(fab)
 ) %>% ungroup()

# remove participants without any data
df.sac = df.sac %>% filter(!is.na(lat)) %>% ungroup() %>%
 # remove unbelievably short and extremely long saccades
 filter(lat <= quantile(lat, probs = 0.99) &
 lat > 100) %>%
 # recode so that it is more consistent
 mutate(
 direction = if_else(dir_face, "face", "object")
 )

# divide into cue and target saccades
criticalpoints = function(density, threshold = 1){
 up = sapply(1:threshold, function(n) c(density$y[-(seq(n))], rep(NA, n)))
 down = sapply(-1:-threshold,
 function(n) c(rep(NA,abs(n)),
 density$y[-seq(length(density$y),
 length(density$y) - abs(n) + 1)]))
 a = cbind(density$y,up,down)
 minima = round(density$x[which(apply(a, 1, min) == a[,1])])
 maxima = round(density$x[which(apply(a, 1, max) == a[,1])])
 return(list(minima = minima, maxima = maxima))
}

points = criticalpoints(density(df.sac$lat))

# get the density of the latencies
dd = with(density(df.sac$lat), data.frame(x,y))

# find which point is the global minimum
lat.points = dd$y[points$minima]
idx = which.min(lat.points)

# print the latency
points$minima[idx]

## [1] 331

# plot it all
p = ggplot(dd, aes(x = x, y = y)) +
 geom_line() +
 geom_vline(xintercept = points$minima[idx], linetype=3) +
 geom_ribbon(data = subset(dd, x <= points$minima[idx]),
 aes(ymax = y, fill = "cue-elicited"), ymin = 0,
 colour = "black", alpha = .8) +
 geom_ribbon(data = subset(dd, x >= points$minima[idx]),
 aes(ymax = y, fill = "target-elicited"), ymin = 0,
 colour = "black", alpha = .8) +
scale_fill_manual(name = "test",
values = c("cue-elicited" = custom.col[7], "target-elicited" = custom.col[3]),
labels = c("Cue-elicited", "Target-elicited")) +
 geom_vline(xintercept = 200) +
 labs(title = "Classification of saccades", x = "Latency", y = "Density") +
 xlim(0, 800) +
 theme_bw() +
 theme(legend.position = "inside", legend.position.inside = c(0.125, 0.8),
 plot.title = element_text(hjust = 0.5),
 legend.title = element_blank(), axis.title = element_text(size = 28),
 axis.text = element_text(size = 28),
 text = element_text(family = "Merriweather", size = 32))

ggsave("Fig2_densLatency.tif", plot = p,
 units = "mm",
 width = 170,
 height = 100,
 dpi = 300)

The graph above shows the density of the latencies with zero on the x-axis being the onset of the cue. After 200ms, the cue disappears and the target is presented on the screen (solid line). We can see that there is a minimum about 130ms after the target appears (dotted line). We can assume that saccades produced before this were in response to the cue (pink) and saccades after were in response to the target (green). Therefore, we divide the saccades accordingly. Then, we aggregate the data per subject and cue. Last, we set all predictors to sum contrasts.

# summarise overall saccade count based on direction: whole trial
df.cnt = df.sac %>%
 group_by(subID, direction) %>%
 summarise(
 n.sac = n()
 )

# add a zero if no saccades were produced
subID = rep(as.character(unique(df.sac$subID)),
 each = length(unique(df.sac$direction)))
direction = rep(as.character(unique(df.sac$direction)),
 times = length(unique(df.sac$subID)))
df.cnt = merge(df.cnt, data.frame(subID, direction), all = T) %>%
 mutate(
 n.sac = if_else(is.na(n.sac), 0, n.sac)
 ) %>%
 # merge with behavioural data
 merge(., df.fab.agg) %>%
 mutate_if(is.character, as.factor)

# code whether or not cue associated saccade occured and capture latencies
df.cue = merge(df.fab,
 df.sac %>% filter(lat <= points$minima[idx] & sac_trl == 1),
 all = T) %>%
 select(subID, diagnosis, trl, stm, cue, rt.cor, acc, direction, lat) %>%
 mutate(
 sac = if_else(is.na(direction),0,1)
 ) %>%
 mutate_if(is.character, as.factor)

# preprocess target latencies
df.lat = df.sac %>%
 # only keep latencies associated with target
 filter(lat > points$minima[idx]) %>%
 # only keep the first target saccade latency of each trial
 group_by(subID, diagnosis, trl, cue) %>%
 filter(sac_trl == min(sac_trl)) %>%
 merge(., df.fab) %>%
 mutate_if(is.character, as.factor)

# set and print the contrasts
contrasts(df.lat$cue) = contr.sum(2)
contrasts(df.lat$cue)

## [,1]
## face 1
## object -1

contrasts(df.lat$diagnosis) = contr.sum(4)
contrasts(df.lat$diagnosis)

## [,1] [,2] [,3]
## ADHD 1 0 0
## ASD 0 1 0
## BOTH 0 0 1
## COMP -1 -1 -1

contrasts(df.cue$direction) = contr.sum(2)
contrasts(df.cue$direction)

## [,1]
## face 1
## object -1

contrasts(df.cue$diagnosis) = contr.sum(4)
contrasts(df.cue$diagnosis)

## [,1] [,2] [,3]
## ADHD 1 0 0
## ASD 0 1 0
## BOTH 0 0 1
## COMP -1 -1 -1

contrasts(df.cnt$direction) = contr.sum(2)
contrasts(df.cnt$direction)

## [,1]
## face 1
## object -1

contrasts(df.cnt$diagnosis) = contr.sum(4)
contrasts(df.cnt$diagnosis)

## [,1] [,2] [,3]
## ADHD 1 0 0
## ASD 0 1 0
## BOTH 0 0 1
## COMP -1 -1 -1

# 8 Number of saccades towards face during trial

First, we are going to assess the saccades that are produced in the direction of the face throughout the full trial: cue and target presentation. Based on Entzmann et al. (2021), we hypothesised that COMP participants produce more saccades towards the face than towards the object cues during the trials.

## 8.1 Specify the model

Since we are counting the number of saccades, we use a poisson distribution for our model. We add an group-level intercept for each subject, and assess the influence of the predictors diagnostic status and whether the saccade was directed towards the side of the face or object as well as the interaction. We set our priors very wide because we do not have strong prior assumptions apart from people producing fewer saccades than there are trials.

code = "CNT"

# set the formula
f.cnt = brms::bf(n.sac ~ diagnosis * direction + (1 | subID))

# set priors based on study design
priors = c(
 prior(normal(3, 1.5), class = Intercept),
 prior(normal(0, 1.0), class = sd),
 prior(normal(0, 1.0), class = b)
)

# set number of iterations and warmup for models
iter = 4500
warm = 1500

## 8.2 Simulation-based calibration

# check if the SBC already exists
if (file.exists(file.path(cache_dir, sprintf("df_res_%s.rds", code)))) {
 # load in the results of the SBC
 df.results = readRDS(file.path(cache_dir, sprintf("df_res_%s.rds", code)))
 df.backend = readRDS(file.path(cache_dir, sprintf("df_div_%s.rds", code)))
 dat = readRDS(file.path(cache_dir, sprintf("dat_%s.rds", code)))
} else {
 # perform the SBC
 set.seed(2468)
 gen = SBC_generator_brms(f.cnt, data = df.cnt, prior = priors,
 thin = 50, warmup = 10000, refresh = 2000,
 generate_lp = TRUE, family = poisson(), init = 0.1)
 bck = SBC_backend_brms_from_generator(gen, chains = 4, thin = 1,
 warmup = warm, iter = iter)
 dat = generate_datasets(gen, nsim)
 saveRDS(dat, file.path(cache_dir, sprintf("dat_%s.rds", code)))
 res = compute_SBC(dat,
 bck,
 cache_mode = "results",
 cache_location = file.path(cache_dir, sprintf("res_%s", code)))
 df.results = res$stats
 df.backend = res$backend_diagnostics
 saveRDS(df.results, file = file.path(cache_dir, paste0("df_res_", code, ".rds")))
 saveRDS(df.backend, file = file.path(cache_dir, paste0("df_div_", code, ".rds")))
}

We start by investigating the rhats and the number of divergent samples. This shows that 6 of 250 simulations had at least one parameter that had an rhat of at least 1.05, and only 0 models had divergent samples. This suggests that this model performs well.

## 8.3 Prior predictive checks

Next, we can plot the simulated values to perform prior predictive checks.

# get the true values
truePars = dat[["variables"]]

# create a matrix out of generated data
dvname = gsub(" ", "", gsub("[\\|~].*", "", f.cnt)[1])
dvfakemat = matrix(NA, nrow(dat[['generated']][[1]]), length(dat[['generated']]))
for (i in 1:length(dat[['generated']])) {
 dvfakemat[,i] = dat[['generated']][[i]][[dvname]]
}

# set very large data points to a value of 432
dvfakematH = dvfakemat;
dvfakematH[dvfakematH > 432] = 432
# compute one histogram per simulated data-set
breaks = seq(0, max(dvfakematH, na.rm=T), length.out = 100)
binwidth = round(breaks[2] - breaks[1])
breaks = seq(0, max(dvfakematH, na.rm=T), binwidth)
histmat = matrix(NA, ncol = nrow(truePars) + binwidth, nrow = length(breaks)-1)
for (i in 1:nrow(truePars)) {
 histmat[,i] = hist(dvfakematH[,i], breaks = breaks, plot = F)$counts
}
# for each bin, compute quantiles across histograms
probs = seq(0.1, 0.9, 0.1)
quantmat= as.data.frame(matrix(NA, nrow=dim(histmat)[1], ncol = length(probs)))
names(quantmat) = paste0("p", probs)
for (i in 1:dim(histmat)[1]) {
 quantmat[i,] = quantile(histmat[i,], p = probs, na.rm = T)
}
quantmat$x = breaks[2:length(breaks)] - binwidth/2 # add bin mean
p1 = ggplot(data = quantmat, aes(x = x)) +
 geom_ribbon(aes(ymax = p0.9, ymin = p0.1), fill = c_light) +
 geom_ribbon(aes(ymax = p0.8, ymin = p0.2), fill = c_light_highlight) +
 geom_ribbon(aes(ymax = p0.7, ymin = p0.3), fill = c_mid) +
 geom_ribbon(aes(ymax = p0.6, ymin = p0.4), fill = c_mid_highlight) +
 geom_line(aes(y = p0.5), colour = c_dark, linewidth = 1) +
 labs(title = "Prior predictive distribution", y = "", x = "number of saccades") +
 theme_bw()

tmpM = apply(dvfakematH, 2, mean) # mean
tmpSD = apply(dvfakematH, 2, sd)
p2 = ggplot() +
 stat_bin(aes(x = tmpM), fill = c_dark) +
 labs(x = "Mean number of saccades", title = "Means of simulated data") +
 theme_bw()
p3 = ggplot() +
 stat_bin(aes(x = tmpSD), fill = c_dark) +
 labs(x = "SD number of saccades", title = "Standard deviations of simulated data") +
 theme_bw()

p = ggarrange(p1,
 ggarrange(p2, p3, ncol = 2, labels = c("B", "C")),
 nrow = 2, labels = "A")
annotate_figure(p, top = text_grob("Prior predictive checks", face = "bold", size = 14))


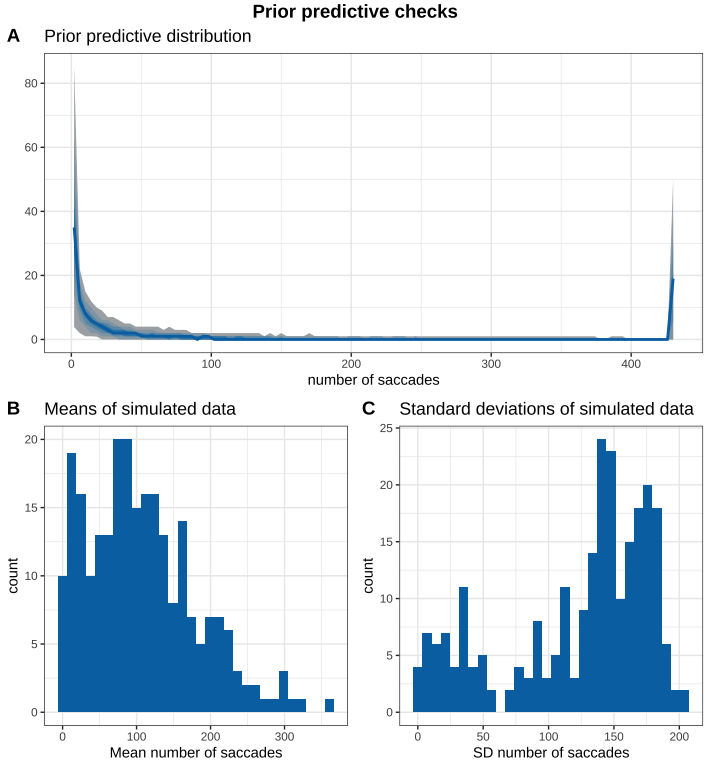


Since our priors were set very wide, we do get wide prior predictive distributions. We accept this and continue with our checks.

## 8.4 Computational faithfulness and model sensitivity

# get simulation numbers with issues
des_rank = max(df.results$max_rank)
check = merge(df.results %>%
 group_by(sim_id) %>%
 summarise(
 rhat = max(rhat, na.rm = T),
 mean_rank = max(max_rank)
 ) %>%
 filter(rhat >= 1.05 | mean_rank < des_rank),
 df.backend %>% filter(n_divergent > 0), all = T)

# plot SBC with functions from the SBC package focusing on population-level parameters

df.results.b = df.results %>%
 filter(substr(variable, 1, 2) == "b_") %>%
 filter(!(sim_id %in% check$sim_id)) %>%
 ungroup() %>%
 mutate(
 max_rank = max(rank)
 )
p1 = plot_ecdf_diff(df.results.b) + theme_bw() + theme(legend.position = "none") +
 scale_x_continuous(breaks=scales::pretty_breaks(n = 3)) +
 scale_y_continuous(breaks=scales::pretty_breaks(n = 3))
p2 = plot_rank_hist(df.results.b, bins = 20) + theme_bw() +
 scale_x_continuous(breaks=scales::pretty_breaks(n = 3)) +
 scale_y_continuous(breaks=scales::pretty_breaks(n = 3))
p3 = plot_sim_estimated(df.results.b, alpha = .8) + theme_bw() +
 scale_x_continuous(breaks=scales::pretty_breaks(n = 3)) +
 scale_y_continuous(breaks=scales::pretty_breaks(n = 3))
p4 = plot_contraction(df.results.b,
 prior_sd = setNames(
 c(as.numeric(
 gsub(".*, (.+)\\).*", "\\1",
 priors[priors$class == "Intercept",]$prior)),
 rep(
 as.numeric(
 gsub(".*, (.+)\\).*", "\\1",
 priors[priors$class == "b",]$prior)),
 length(unique(df.results.b$variable))-1)),
 unique(df.results.b$variable))) +
 theme_bw() +
 scale_x_continuous(breaks=scales::pretty_breaks(n = 3)) +
 scale_y_continuous(breaks=scales::pretty_breaks(n = 3))

p = ggarrange(p1, p2, p3, p4, labels = "AUTO", ncol = 1, nrow = 4)
annotate_figure(p,
 top = text_grob("Computational faithfulness and model sensitivity",
 face = "bold", size = 14))


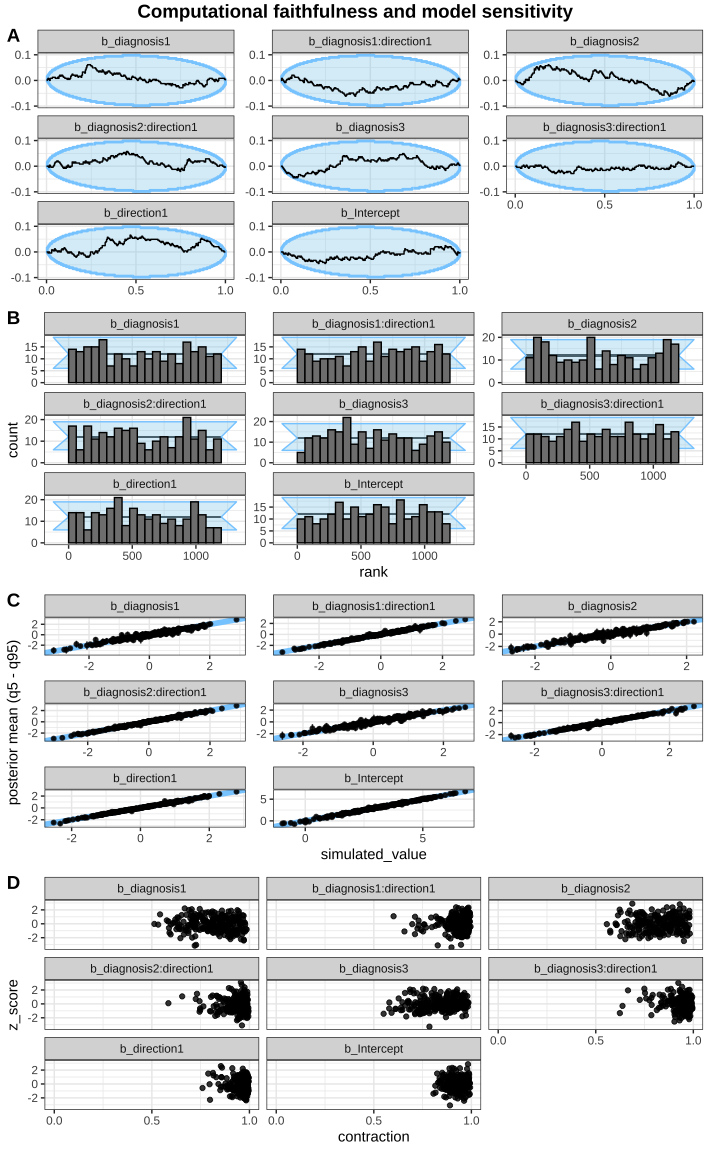


All of this looks good. Despite our wide priors, the contraction shows a bit of a distribution which increases our trust that the wide priors are appropriate.

## 8.5 Posterior predictive checks

As the next step, we fit the model and check whether the chains have converged, which they seem to have. We then perform posterior predictive checks on the model using the bayesplot package.

# fit the model
set.seed(2468)
m.cnt = brm(f.cnt,
 df.cnt, prior = priors,
 iter = iter, warmup = warm,
 backend = "cmdstanr", threads = threading(8),
 file = "m_cnt-face",
 family = "poisson",
 save_pars = save_pars(all = TRUE)
 )
rstan::check_hmc_diagnostics(m.cnt$fit)

##
## Divergences:

## 0 of 12000 iterations ended with a divergence.

##
## Tree depth:

## 0 of 12000 iterations saturated the maximum tree depth of 10.

##
## Energy:

## E-BFMI indicated no pathological behavior.

# check that rhats are below 1.01
sum(brms::rhat(m.cnt) >= 1.01, na.rm = T)

## [1] 0

# check the trace plots
post.draws = as_draws_df(m.cnt)
mcmc_trace(post.draws, regex_pars = "^b_",
 facet_args = list(ncol = 3)) +
 scale_x_continuous(breaks=scales::pretty_breaks(n = 3)) +
 scale_y_continuous(breaks=scales::pretty_breaks(n = 3))

## Scale for x is already present.
## Adding another scale for x, which will replace the existing scale.


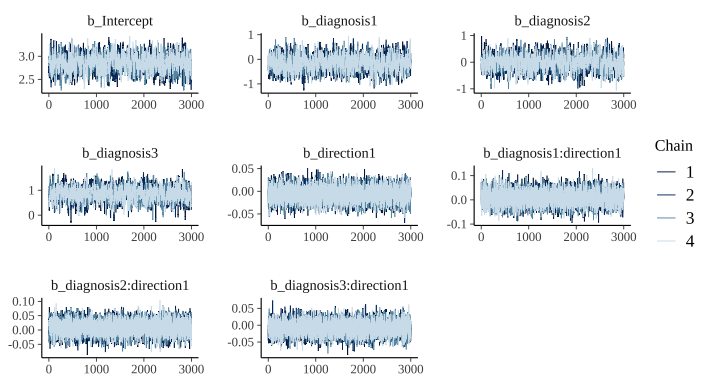


This model has no divergent samples and no rhats that are higher or equal to 1.01. Therefore, we go ahead and perform our posterior predictive checks.

# get the posterior predictions
post.pred = posterior_predict(m.cnt, ndraws = nsim)

# check the fit of the predicted data compared to the real data
p1 = pp_check(m.cnt, ndraws = nsim) +
 theme_bw()

# distributions of means and sds compared to the real values per group
p2 = ppc_stat_grouped(df.cnt$n.sac, post.pred, df.cnt$diagnosis) +
 theme_bw()

p = ggarrange(p1, p2,
 nrow = 2, ncol = 1, labels = "AUTO")
annotate_figure(p,
 top = text_grob("Posterior predictive checks: number of saccades towards face",
 face = "bold", size = 14))


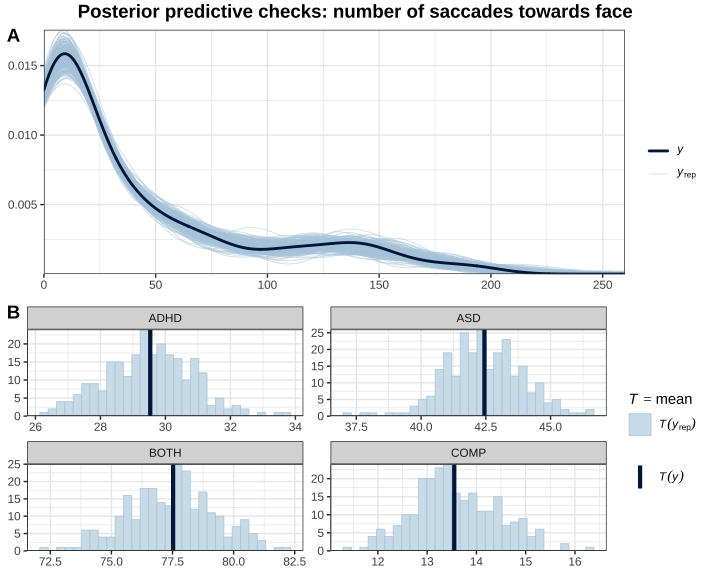


The predictions based on the model capture the data well. This further increases our trust in the model.

## 8.6 Inferences

Now that we are convinced that we can trust our model, we have a look at the model and its estimates.

# print a summary
summary(m.cnt)

## Family: poisson
## Links: mu = log
## Formula: n.sac ~ diagnosis * direction + (1 | subID)
## Data: df.cnt (Number of observations: 150)
## Draws: 4 chains, each with iter = 4500; warmup = 1500; thin = 1;
## total post-warmup draws = 12000
##
## Multilevel Hyperparameters:
## ~subID (Number of levels: 75)
## Estimate Est.Error l-95% CI u-95% CI Rhat Bulk_ESS Tail_ESS
## sd(Intercept) 1.40 0.13 1.18 1.68 1.00 1641 3249
##
## Regression Coefficients:
## Estimate Est.Error l-95% CI u-95% CI Rhat Bulk_ESS
## Intercept 2.86 0.17 2.52 3.19 1.00 1307
## diagnosis1 -0.15 0.30 -0.73 0.43 1.00 1381
## diagnosis2 -0.01 0.27 -0.55 0.53 1.00 1119
## diagnosis3 0.84 0.27 0.29 1.36 1.00 1112
## direction1 -0.01 0.02 -0.04 0.02 1.00 7199
## diagnosis1:direction1 0.01 0.03 -0.05 0.06 1.00 8978
## diagnosis2:direction1 0.01 0.02 -0.04 0.05 1.00 8923
## diagnosis3:direction1 -0.01 0.02 -0.05 0.03 1.00 8022
## Tail_ESS
## Intercept 2576
## diagnosis1 2640
## diagnosis2 2341
## diagnosis3 1912
## direction1 7950
## diagnosis1:direction1 7990
## diagnosis2:direction1 8625
## diagnosis3:direction1 8414
##
## Draws were sampled using sample(hmc). For each parameter, Bulk_ESS
## and Tail_ESS are effective sample size measures, and Rhat is the potential
## scale reduction factor on split chains (at convergence, Rhat = 1).

# get the estimates and compute groups
df.m.cnt = as_draws_df(m.cnt) %>%
 select(starts_with("b_")) %>%
 mutate(
 b_COMP = - b_diagnosis1 - b_diagnosis2 - b_diagnosis3,
 ASD = b_Intercept + b_diagnosis2,
 ADHD = b_Intercept + b_diagnosis1,
 BOTH = b_Intercept + b_diagnosis3,
 COMP = b_Intercept + b_COMP
 )

# plot the posterior distributions
df.m.cnt %>%
 select(starts_with("b_")) %>%
 pivot_longer(cols = starts_with("b_"), names_to = "coef", values_to = "estimate") %>%
 filter(coef != "b_Intercept") %>%
 mutate(
 coef = case_match(coef,
 "b_diagnosis1" ~ "ADHD",
 "b_diagnosis2" ~ "ASD",
 "b_diagnosis3" ~ "ADHD+ASD",
 "b_COMP" ~ "COMP",
 "b_direction1" ~ "Face",
 "b_diagnosis1:direction1" ~ "Interaction: ADHD",
 "b_diagnosis2:direction1" ~ "Interaction: ASD",
 "b_diagnosis3:direction1" ~ "Interaction: ADHD+ASD"
 ),
 coef = fct_reorder(coef, desc(estimate))
 ) %>%
 group_by(coef) %>%
 mutate(
 cred = case_when(
 (mean(estimate) < 0 & quantile(estimate, probs = 0.975) < 0) |
 (mean(estimate) > 0 & quantile(estimate, probs = 0.025) > 0) ~ "credible",
 T ~ "not credible"
 )
 ) %>% ungroup() %>%
 ggplot(aes(x = estimate, y = coef, fill = cred)) +
 geom_vline(xintercept = 0, linetype = 'dashed') +
 ggdist::stat_halfeye(alpha = 0.7) + ylab(NULL) + theme_bw() +
 scale_fill_manual(values = c(credible = c_dark, c_light)) +
 theme(legend.position = "none")


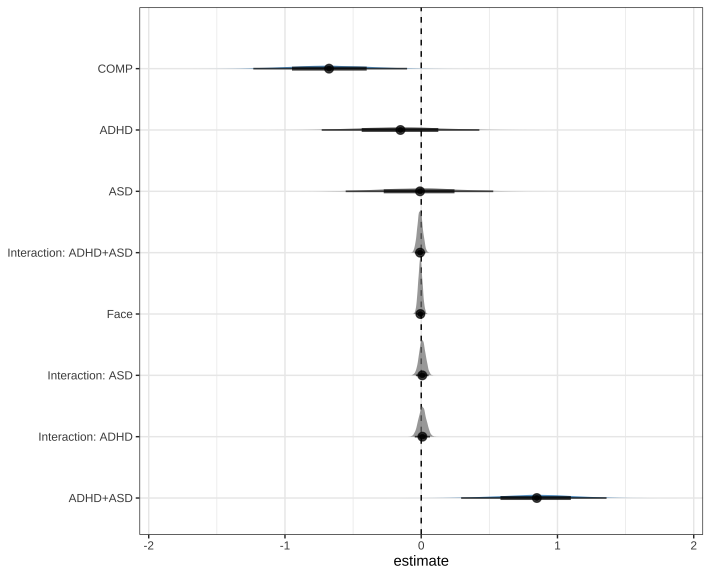


# H2a: COMP: face > object
h2a = hypothesis(m.cnt,
 "0 < 2*(direction1 - diagnosis1:direction1 -
 diagnosis2:direction1 - diagnosis3:direction1)")
h2a

## Hypothesis Tests for class b:
## Hypothesis Estimate Est.Error CI.Lower CI.Upper Evid.Ratio
## 1 (0)-(2*(direction... < 0 0.03 0.09 -0.11 0.17 0.57
## Post.Prob Star
## 1 0.36
## ---
## 'CI': 90%-CI for one-sided and 95%-CI for two-sided hypotheses.
## '*': For one-sided hypotheses, the posterior probability exceeds 95%;
## for two-sided hypotheses, the value tested against lies outside the 95%-CI.
## Posterior probabilities of point hypotheses assume equal prior probabilities.

# explore general FAB
e1 = hypothesis(m.cnt, "0 < 2*direction1",
 alpha = 0.025)
e1

## Hypothesis Tests for class b:
## Hypothesis Estimate Est.Error CI.Lower CI.Upper Evid.Ratio
## 1 (0)-(2*direction1) < 0 0.01 0.03 -0.05 0.07 0.52
## Post.Prob Star
## 1 0.34
## ---
## 'CI': 95%-CI for one-sided and 97.5%-CI for two-sided hypotheses.
## '*': For one-sided hypotheses, the posterior probability exceeds 97.5%;
## for two-sided hypotheses, the value tested against lies outside the 97.5%-CI.
## Posterior probabilities of point hypotheses assume equal prior probabilities.

# extract predicted differences
df.new = df.cnt %>%
 select(diagnosis, direction) %>%
 distinct() %>%
 mutate(
 condition = paste(diagnosis, direction, sep = "_")
 )
df.ms = as.data.frame(
 fitted(m.cnt, summary = F,
 newdata = df.new %>% select(diagnosis, direction),
 re_formula = NA))
colnames(df.ms) = df.new$condition

st(df.ms,
 summ = c('mean(x)','sd(x)','min(x)','pctile(x)[2.5]',
 'pctile(x)[97.5]','max(x)'))

Summary Statistics

| Variable | Mean | Sd | Min | Pctile[2.5] | Pctile[97.5] | Max |
| --- | --- | --- | --- | --- | --- | --- |
| COMP_face | 9.2 | 3.1 | 2.7 | 4.4 | 16 | 31 |
| COMP_object | 9.5 | 3.2 | 2.5 | 4.5 | 17 | 30 |
| ADHD_face | 16 | 5.8 | 3.6 | 7.2 | 29 | 54 |
| ADHD_object | 16 | 5.8 | 4 | 7.1 | 29 | 55 |
| ASD_face | 18 | 6 | 5.4 | 8.8 | 31 | 61 |
| ASD_object | 18 | 6 | 5.3 | 8.8 | 31 | 62 |
| BOTH_face | 42 | 13 | 8.8 | 21 | 70 | 134 |
| BOTH_object | 43 | 13 | 9.8 | 21 | 73 | 136 |

st(df.ms %>%
 mutate(
 face = rowMeans(select(., matches(".*_face")), na.rm = T),
 object = rowMeans(select(., matches(".*_object")), na.rm = T),
 FAB = object - face
 ) %>% select(face, object, FAB),
 summ = c('mean(x)','sd(x)','min(x)','pctile(x)[2.5]','pctile(x)[97.5]','max(x)'))

Summary Statistics

| Variable | Mean | Sd | Min | Pctile[2.5] | Pctile[97.5] | Max |
| --- | --- | --- | --- | --- | --- | --- |
| face | 21 | 4 | 11 | 14 | 30 | 44 |
| object | 22 | 4.1 | 11 | 15 | 30 | 44 |
| FAB | 0.35 | 0.59 | -2.8 | -0.81 | 1.5 | 3 |

Our hypothesis regarding the number of saccades towards face or object cues was not confirmed: there was no credible difference in our COMP group (*estimate* = 0.03 [-0.11, 0.17], *posterior probability* = 36.33%). Exploration of FAB effect regardless of the group similarly indicates no FAB in the form of a larger number of saccades produced towards the faces over the whole trial (*estimate* = 0.01 [-0.05, 0.07], *posterior probability* = 34.08%).

## 8.7 Plots

As a next step, we can now finally plot our data.

# rain cloud plot
p = df.cnt %>%
 mutate(
 diagnosis = recode(diagnosis, "BOTH" = "ADHD+ASD"),
 Direction = recode(direction, "face" = "Towards face cue", "object" = "Towards object cue")
 ) %>%
 ggplot(aes(diagnosis, n.sac, fill = Direction, colour = Direction)) + #
 geom_rain(rain.side = 'r',
boxplot.args = list(color = "black", outlier.shape = NA, show_guide = FALSE, alpha = .8),
violin.args = list(color = "black", outlier.shape = NA, alpha = .8),
boxplot.args.pos = list(
 position = ggpp::position_dodgenudge(x = 0, width = 0.3), width = 0.3
),
point.args = list(show_guide = FALSE, alpha = .5),
violin.args.pos = list(
 width = 0.6, position = position_nudge(x = 0.16)),
point.args.pos = list(position = ggpp::position_dodgenudge(x = -0.25, width = 0.1))) +
 scale_fill_manual(values = custom.col2) +
 scale_color_manual(values = custom.col2) +
 labs(title = "Number of saccades", x = "", y = "Count") +
 theme_bw() +
 theme(legend.position = "inside", legend.position.inside = c(0.84, 0.8),
 plot.title = element_text(hjust = 0.5),
 #legend.direction = "horizontal",
 legend.title=element_blank(),
 axis.title.y = element_text(size = 28),
 axis.text.x = element_text(size = 28, colour = "black"),
 axis.text.y = element_text(size = 24),
 text = element_text(family = "Merriweather", size = 32))

ggsave("Fig5_nrSac.tif", plot = p,
 units = "mm",
 width = 170,
 height = 100,
 dpi = 300)

# 9 Latencies of target-elicited saccades

Next, we focus on the latencies of the saccades that are produced during the presentation of the targets to assess whether cue type, diagnostic group or their interaction influence latencies. We hypothesised that ASD participants show an increased latency compared to COMP participants when producing saccades towards targets appearing at the location of a face.

## 9.1 Full model

We start with the model containing all latencies of saccades produced during the target presentation. We choose a shifted lognormal distribution because saccade latencies below 100ms are very unlikely. Additionally, latencies are determined based on the onset of the cue presentation (200ms). The SBC was run on three groups.

### 9.1.1 Setting up and assessing the model

code = "LAT"

# set the formula
f.lat = brms::bf(lat ~ diagnosis * cue + (cue | subID) + (diagnosis * cue | stm))

# set weakly informative priors
priors = c(
 prior(normal(5, 0.75), class = Intercept),
 prior(normal(0, 0.25), class = sd),
 prior(normal(0, 0.25), class = b),
 prior(normal(0.5, 0.50), class = sigma),
 prior(normal(350, 50.00), class = ndt), # threshold between target and cue saccades
 prior(lkj(2), class = cor)
)

# set number of iterations and warmup for models
iter = 3000
warm = 1000

if (file.exists(file.path(cache_dir, paste0("df_res_", code, ".rds")))) {
 # load in the results of the SBC
 df.results = readRDS(file.path(cache_dir, paste0("df_res_", code, ".rds")))
 df.backend = readRDS(file = file.path(cache_dir, paste0("df_div_", code, ".rds")))
 dat = readRDS(file = file.path(cache_dir, paste0("dat_", code, ".rds")))
} else {
 # create the data and the results
 set.seed(2468)
 gen = SBC_generator_brms(f.lat, data = df.lat, prior = priors,
 family = "shifted_lognormal",
 thin = 50, warmup = 10000, refresh = 2000,
 generate_lp = TRUE)
 dat = generate_datasets(gen, nsim)
 saveRDS(dat, file = file.path(cache_dir, paste0("dat_", code, ".rds")))
 backend = SBC_backend_brms_from_generator(gen, chains = 4, thin = 1,
 warmup = 1000, iter = 3000)
 results = compute_SBC(dat, backend,
 cache_mode = "results",
 cache_location = file.path(cache_dir, paste0("res_", code)))
 saveRDS(results$stats,
 file = file.path(cache_dir, paste0("df_res_", code, ".rds")))
 saveRDS(results$backend_diagnostics,
 file = file.path(cache_dir, paste0("df_div_", code, ".rds")))
}

We start by investigating the rhats and the number of divergent samples. This shows that 0 of 250 simulations had at least one parameter that had an rhat of at least 1.05 and only 1 model had divergent samples (mean number of samples of the simulations with divergent samples: 1). This suggests that this model performs well.

### 9.1.2 Prior predictive checks

Next, we can plot the simulated values to perform prior predictive checks.

# get the true values
truePars = dat[["variables"]]

# create a matrix out of generated data
dvname = gsub(" ", "", gsub("[\\|~].*", "", f.lat)[1])
dvfakemat = matrix(NA, nrow(dat[['generated']][[1]]), length(dat[['generated']]))
for (i in 1:length(dat[['generated']])) {
 dvfakemat[,i] = dat[['generated']][[i]][[dvname]]
}

# set very large data points to a value of 1500
dvfakematH = dvfakemat;
dvfakematH[dvfakematH < 0] = 0
dvfakematH[dvfakematH > 1500] = 1500
# compute one histogram per simulated data-set
breaks = seq(0, 1500, length.out = 101)
binwidth = breaks[2] - breaks[1]
histmat = matrix(NA, ncol = nrow(truePars) + binwidth, nrow = length(breaks)-1)
for (i in 1:nrow(truePars)) {
 histmat[,i] = hist(dvfakematH[,i], breaks = breaks, plot = F)$counts
}
# for each bin, compute quantiles across histograms
probs = seq(0.1, 0.9, 0.1)
quantmat= as.data.frame(matrix(NA, nrow=dim(histmat)[1], ncol = length(probs)))
names(quantmat) = paste0("p", probs)
for (i in 1:dim(histmat)[1]) {
 quantmat[i,] = quantile(histmat[i,], p = probs, na.rm = T)
}
quantmat$x = breaks[2:length(breaks)] - binwidth/2 # add bin mean
p1 = ggplot(data = quantmat, aes(x = x)) +
 geom_ribbon(aes(ymax = p0.9, ymin = p0.1), fill = c_light) +
 geom_ribbon(aes(ymax = p0.8, ymin = p0.2), fill = c_light_highlight) +
 geom_ribbon(aes(ymax = p0.7, ymin = p0.3), fill = c_mid) +
 geom_ribbon(aes(ymax = p0.6, ymin = p0.4), fill = c_mid_highlight) +
 geom_line(aes(y = p0.5), colour = c_dark, linewidth = 1) +
 labs(title = "Prior predictive distribution", y = "", x = "latency of saccades") +
 theme_bw()

tmpM = apply(dvfakemat, 2, mean) # mean
tmpSD = apply(dvfakemat, 2, sd)
p2 = ggplot() +
 stat_bin(aes(x = tmpM), fill = c_dark) +
 labs(x = "Mean latency of saccades", title = "Means of simulated data") +
 theme_bw()
p3 = ggplot() +
 stat_bin(aes(x = tmpSD), fill = c_dark) +
 labs(x = "SD latency of saccades", title = "Standard deviations of simulated data") +
 theme_bw()

p = ggarrange(p1,
 ggarrange(p2, p3, ncol = 2, labels = c("B", "C")),
 nrow = 2, labels = "A")
annotate_figure(p,
 top = text_grob("Prior predictive checks: latency",
 face = "bold", size = 14))


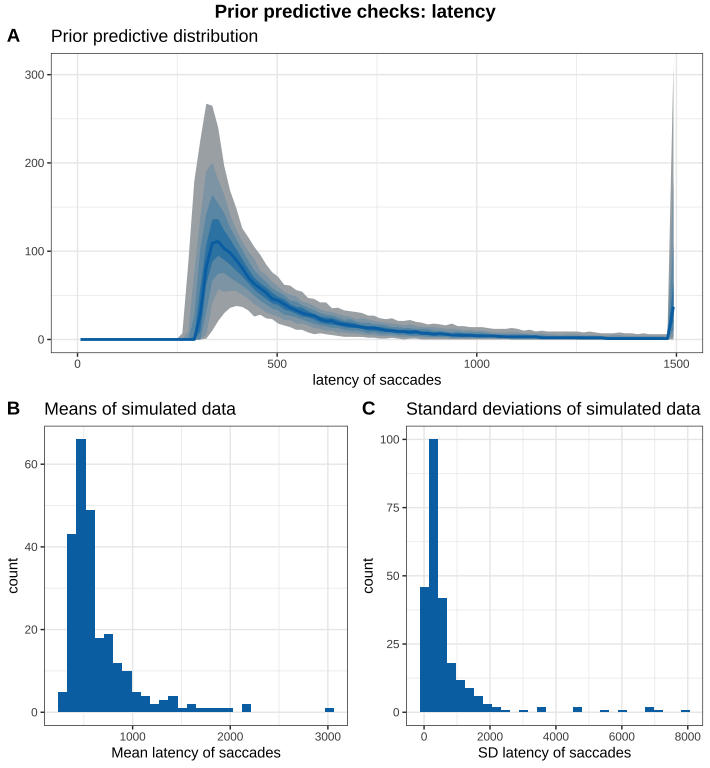


First, we assess whether the simulated values fit our expectations of the distribution of the data. Previous literature has found that saccade latencies are around 200ms with few saccades being produced faster than 100ms. If we add 200ms from the cue presentation, this means we expect most latencies to be above 300ms and centered around 400ms. Our simulated datasets seem to capture this well.

### 9.1.3 Computational faithfulness and model sensitivity

# get simulation numbers with issues
des_rank = max(df.results$max_rank)
check = merge(df.results %>%
 group_by(sim_id) %>%
 summarise(
 rhat = max(rhat, na.rm = T),
 mean_rank = max(max_rank)
 ) %>%
 filter(rhat >= 1.05 | mean_rank < des_rank),
 df.backend %>% filter(n_divergent > 0), all = T)

# plot SBC with functions from the SBC package focusing on population-level parameters

df.results.b = df.results %>%
 filter(substr(variable, 1, 2) == "b_") %>%
 filter(!(sim_id %in% check$sim_id)) %>%
 ungroup() %>%
 mutate(
 max_rank = max(rank)
 )
p1 = plot_ecdf_diff(df.results.b) + theme_bw() + theme(legend.position = "none") +
 scale_x_continuous(breaks=scales::pretty_breaks(n = 3)) +
 scale_y_continuous(breaks=scales::pretty_breaks(n = 3))
p2 = plot_rank_hist(df.results.b, bins = 20) + theme_bw() +
 scale_x_continuous(breaks=scales::pretty_breaks(n = 3)) +
 scale_y_continuous(breaks=scales::pretty_breaks(n = 3))
p3 = plot_sim_estimated(df.results.b, alpha = .8) + theme_bw() +
 scale_x_continuous(breaks=scales::pretty_breaks(n = 3)) +
 scale_y_continuous(breaks=scales::pretty_breaks(n = 3))
p4 = plot_contraction(df.results.b,
 prior_sd = setNames(
 c(as.numeric(
 gsub(".*, (.+)\\).*", "\\1",
 priors[priors$class == "Intercept",]$prior)),
 rep(
 as.numeric(
 gsub(".*, (.+)\\).*", "\\1",
 priors[priors$class == "b",]$prior)),
 length(unique(df.results.b$variable))-1)),
 unique(df.results.b$variable))) +
 theme_bw() +
 scale_x_continuous(breaks=scales::pretty_breaks(n = 3)) +
 scale_y_continuous(breaks=scales::pretty_breaks(n = 3))

p = ggarrange(p1, p2, p3, p4, labels = "AUTO", ncol = 1, nrow = 4)
annotate_figure(p,
 top = text_grob("Computational faithfulness and model sensitivity",
 face = "bold", size = 14))


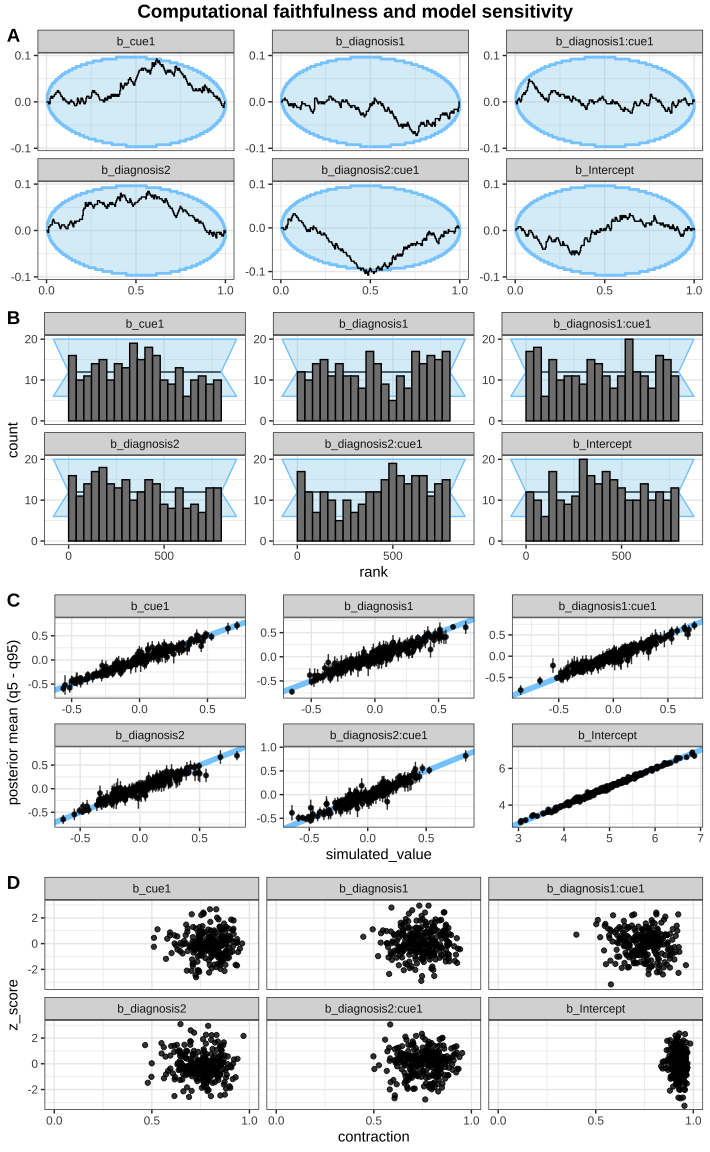


All looks acceptable here.

### 9.1.4 Posterior predictive checks

As the next step, we fit the model and check whether the chains have converged, which they seem to have. We then perform posterior predictive checks on the model using the bayesplot package.

# fit the maximal model
set.seed(2587)
m.lat = brm(f.lat,
 df.lat, prior = priors,
 iter = iter, warmup = warm,
 backend = "cmdstanr", threads = threading(8),
 family = "shifted_lognormal",
 file = "m_lat",
 save_pars = save_pars(all = TRUE)
 )
rstan::check_hmc_diagnostics(m.lat$fit)

##
## Divergences:

## 0 of 8000 iterations ended with a divergence.

##
## Tree depth:

## 0 of 8000 iterations saturated the maximum tree depth of 10.

##
## Energy:

## E-BFMI indicated no pathological behavior.

# check that rhats are below 1.01
sum(brms::rhat(m.lat) >= 1.01, na.rm = T)

## [1] 5

# check the trace plots
post.draws = as_draws_df(m.lat)
mcmc_trace(post.draws, regex_pars = "^b_",
 facet_args = list(ncol = 3)) +
 scale_x_continuous(breaks=scales::pretty_breaks(n = 3)) +
 scale_y_continuous(breaks=scales::pretty_breaks(n = 3))

## Scale for x is already present.
## Adding another scale for x, which will replace the existing scale.


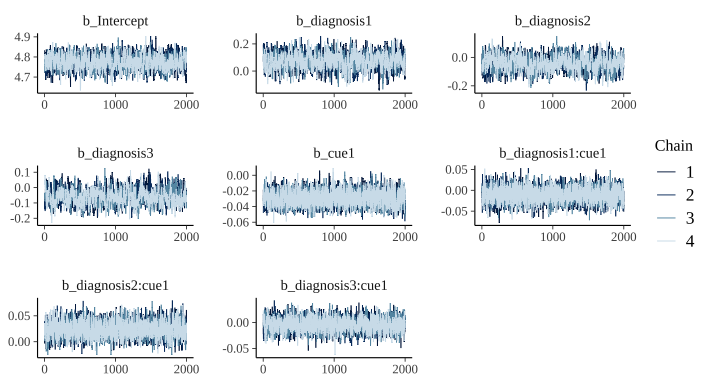


The model does not have any divergent transitions nor high rhats. The trace plots also look good, therefore, we move on to the posterior predictive checks.

# get the posterior predictions
post.pred = posterior_predict(m.lat, ndraws = nsim)

# check the fit of the predicted data compared to the real data
p1 = pp_check(m.lat, ndraws = nsim) +
 theme_bw() + theme(legend.position = "none")

# distributions of means and sds compared to the real values per group
p2 = ppc_stat_grouped(df.lat$lat, post.pred, df.lat$diagnosis) +
 theme_bw() + theme(legend.position = "none")

p = ggarrange(p1, p2,
 nrow = 2, ncol = 1, labels = "AUTO")
annotate_figure(p,
 top = text_grob("Posterior predictive checks: latency",
 face = "bold", size = 14))


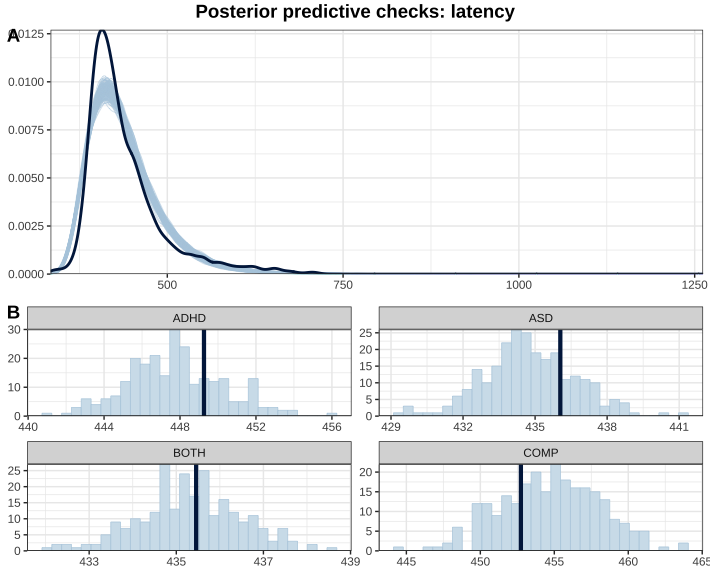


The simulated data based on the model does not fit our data very well: it is wider and seems to underestimate latencies for COMP while overestimating for ADHD and ASD with the dark blue line showing the mean of the actual dataset and the light blue bars showing the distribution of the predicted data.

## 9.2 Aggregated model

Since we want to base our inferences on the estimates, we go back to the drawing board and aggregate our data to see whether this resolves these issues.

### 9.2.1 Setting up and assessing the model

code = "LAT_agg"

# aggregate the data
df.lat.agg = df.lat %>%
 group_by(subID, cue, diagnosis) %>%
 summarise(lat = median(lat, na.rm = T))

# set the formula
f.lat = brms::bf(lat ~ diagnosis * cue + (1 | subID) )

# set weakly informative priors
priors = priors %>% filter(class != "cor")

# set number of iterations and warmup for models
iter = 3000
warm = 1000

Again, we ran the SBC based on the three original, preregistered groups.

if (file.exists(file.path(cache_dir, paste0("df_res_", code, ".rds")))) {
 # load in the results of the SBC
 df.results = readRDS(file.path(cache_dir, paste0("df_res_", code, ".rds")))
 df.backend = readRDS(file = file.path(cache_dir, paste0("df_div_", code, ".rds")))
 dat = readRDS(file = file.path(cache_dir, paste0("dat_", code, ".rds")))
} else {
 # create the data and the results
 set.seed(2468)
 gen = SBC_generator_brms(f.lat, data = df.lat.agg, prior = priors,
 family = "shifted_lognormal",
 thin = 50, warmup = 10000, refresh = 2000,
 generate_lp = TRUE)
 dat = generate_datasets(gen, nsim)
 saveRDS(dat, file = file.path(cache_dir, paste0("dat_", code, ".rds")))
 backend = SBC_backend_brms_from_generator(gen, chains = 4, thin = 1,
 warmup = 1000, iter = 3000)
 results = compute_SBC(dat, backend,
 cache_mode = "results",
 cache_location = file.path(cache_dir, paste0("res_", code)))
 saveRDS(results$stats,
 file = file.path(cache_dir, paste0("df_res_", code, ".rds")))
 saveRDS(results$backend_diagnostics,
 file = file.path(cache_dir, paste0("df_div_", code, ".rds")))
}

We start by investigating the rhats and the number of divergent samples. This shows that 1 of 250 simulations had at least one parameter that had an rhat of at least 1.05, but 105 models had divergent samples (mean number of samples of the simulations with divergent samples: 7.21). This is something to look out for in the final model.

### 9.2.2 Prior predictive checks

Next, we can plot the simulated values to perform prior predictive checks.

# get the true values
truePars = dat[["variables"]]

# create a matrix out of generated data
dvname = gsub(" ", "", gsub("[\\|~].*", "", f.lat)[1])
dvfakemat = matrix(NA, nrow(dat[['generated']][[1]]), length(dat[['generated']]))
for (i in 1:length(dat[['generated']])) {
 dvfakemat[,i] = dat[['generated']][[i]][[dvname]]
}

# set very large data points to a value of 1500
dvfakematH = dvfakemat;
dvfakematH[dvfakematH < 0] = 0
dvfakematH[dvfakematH > 1500] = 1500
# compute one histogram per simulated data-set
breaks = seq(0, 1500, length.out = 101)
binwidth = breaks[2] - breaks[1]
histmat = matrix(NA, ncol = nrow(truePars) + binwidth, nrow = length(breaks)-1)
for (i in 1:nrow(truePars)) {
 histmat[,i] = hist(dvfakematH[,i], breaks = breaks, plot = F)$counts
}
# for each bin, compute quantiles across histograms
probs = seq(0.1, 0.9, 0.1)
quantmat= as.data.frame(matrix(NA, nrow=dim(histmat)[1], ncol = length(probs)))
names(quantmat) = paste0("p", probs)
for (i in 1:dim(histmat)[1]) {
 quantmat[i,] = quantile(histmat[i,], p = probs, na.rm = T)
}
quantmat$x = breaks[2:length(breaks)] - binwidth/2 # add bin mean
p1 = ggplot(data = quantmat, aes(x = x)) +
 geom_ribbon(aes(ymax = p0.9, ymin = p0.1), fill = c_light) +
 geom_ribbon(aes(ymax = p0.8, ymin = p0.2), fill = c_light_highlight) +
 geom_ribbon(aes(ymax = p0.7, ymin = p0.3), fill = c_mid) +
 geom_ribbon(aes(ymax = p0.6, ymin = p0.4), fill = c_mid_highlight) +
 geom_line(aes(y = p0.5), colour = c_dark, linewidth = 1) +
 labs(title = "Prior predictive distribution", y = "", x = "latency of saccades") +
 theme_bw()

tmpM = apply(dvfakemat, 2, mean) # mean
tmpSD = apply(dvfakemat, 2, sd)
p2 = ggplot() +
 stat_bin(aes(x = tmpM), fill = c_dark) +
 labs(x = "Mean latency of saccades", title = "Means of simulated data") +
 theme_bw()
p3 = ggplot() +
 stat_bin(aes(x = tmpSD), fill = c_dark) +
 labs(x = "SD latency of saccades", title = "Standard deviations of simulated data") +
 theme_bw()

p = ggarrange(p1,
 ggarrange(p2, p3, ncol = 2, labels = c("B", "C")),
 nrow = 2, labels = "A")
annotate_figure(p,
 top = text_grob("Prior predictive checks: latency",
 face = "bold", size = 14))


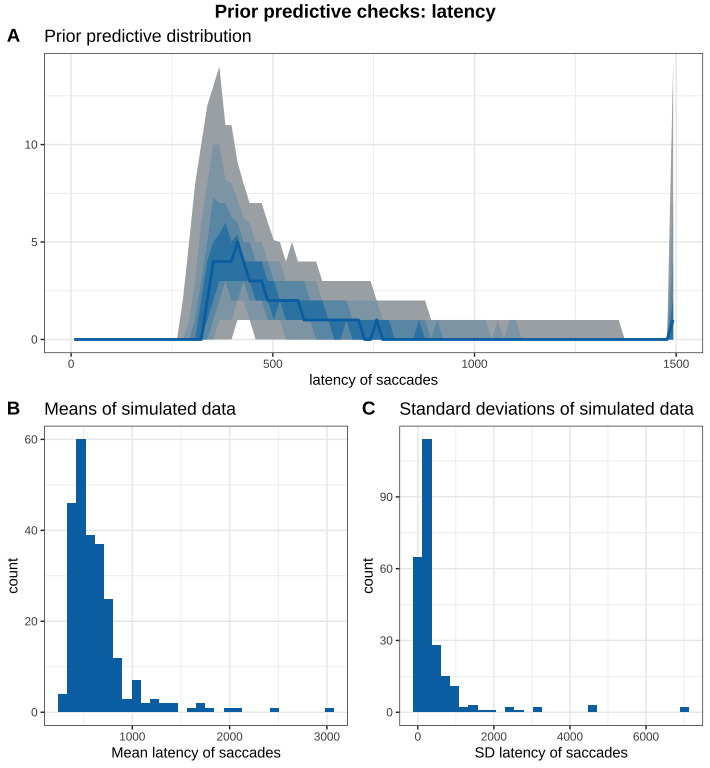


Again, our simulated datasets seem to capture well what we know about saccade latencies.

### 9.2.3 Computational faithfulness and model sensitivity

# get simulation numbers with issues
des_rank = max(df.results$max_rank)
check = merge(df.results %>%
 group_by(sim_id) %>%
 summarise(
 rhat = max(rhat, na.rm = T),
 mean_rank = max(max_rank)
 ) %>%
 filter(rhat >= 1.05 | mean_rank < des_rank),
 df.backend %>% filter(n_divergent > 0), all = T)

# plot SBC with functions from the SBC package focusing on population-level parameters

df.results.b = df.results %>%
 filter(substr(variable, 1, 2) == "b_") %>%
 filter(!(sim_id %in% check$sim_id)) %>%
 ungroup() %>%
 mutate(
 max_rank = max(rank)
 )
p1 = plot_ecdf_diff(df.results.b) + theme_bw() + theme(legend.position = "none") +
 scale_x_continuous(breaks=scales::pretty_breaks(n = 3)) +
 scale_y_continuous(breaks=scales::pretty_breaks(n = 3))
p2 = plot_rank_hist(df.results.b, bins = 20) + theme_bw() +
 scale_x_continuous(breaks=scales::pretty_breaks(n = 3)) +
 scale_y_continuous(breaks=scales::pretty_breaks(n = 3))
p3 = plot_sim_estimated(df.results.b, alpha = .8) + theme_bw() +
 scale_x_continuous(breaks=scales::pretty_breaks(n = 3)) +
 scale_y_continuous(breaks=scales::pretty_breaks(n = 3))
p4 = plot_contraction(df.results.b,
 prior_sd = setNames(
 c(as.numeric(
 gsub(".*, (.+)\\).*", "\\1",
 priors[priors$class == "Intercept",]$prior)),
 rep(
 as.numeric(
 gsub(".*, (.+)\\).*", "\\1",
 priors[priors$class == "b",]$prior)),
 length(unique(df.results.b$variable))-1)),
 unique(df.results.b$variable))) +
 theme_bw() +
 scale_x_continuous(breaks=scales::pretty_breaks(n = 3)) +
 scale_y_continuous(breaks=scales::pretty_breaks(n = 3))

p = ggarrange(p1, p2, p3, p4, labels = "AUTO", ncol = 1, nrow = 4)
annotate_figure(p,
 top = text_grob("Computational faithfulness and model sensitivity",
 face = "bold", size = 14))


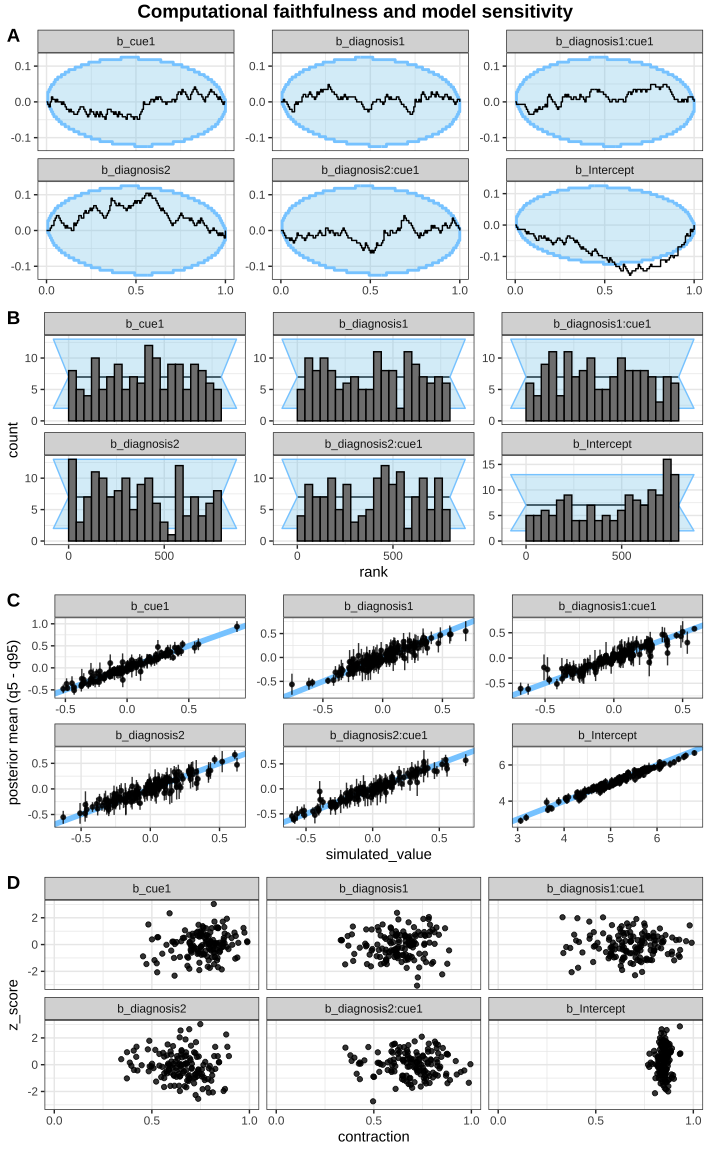


The intercept looks slightly off here, the model could have a slight tendency to underestimate it.

### 9.2.4 Posterior predictive checks

As the next step, we fit the model and check whether the chains have converged, which they seem to have. We then perform posterior predictive checks on the model using the bayesplot package.

# fit the maximal model
set.seed(7799)
m.lat = brm(f.lat,
 df.lat.agg, prior = priors,
 iter = iter, warmup = warm,
 backend = "cmdstanr", threads = threading(8),
 family = "shifted_lognormal",
 file = "m_lat_agg",
 save_pars = save_pars(all = TRUE)
 )
rstan::check_hmc_diagnostics(m.lat$fit)

##
## Divergences:

## 0 of 8000 iterations ended with a divergence.

##
## Tree depth:

## 0 of 8000 iterations saturated the maximum tree depth of 10.

##
## Energy:

## E-BFMI indicated no pathological behavior.

# check that rhats are below 1.01
sum(brms::rhat(m.lat) >= 1.01, na.rm = T)

## [1] 0

# check the trace plots
post.draws = as_draws_df(m.lat)
mcmc_trace(post.draws, regex_pars = "^b_",
 facet_args = list(ncol = 3)) +
 scale_x_continuous(breaks=scales::pretty_breaks(n = 3)) +
 scale_y_continuous(breaks=scales::pretty_breaks(n = 3))

## Scale for x is already present.
## Adding another scale for x, which will replace the existing scale.


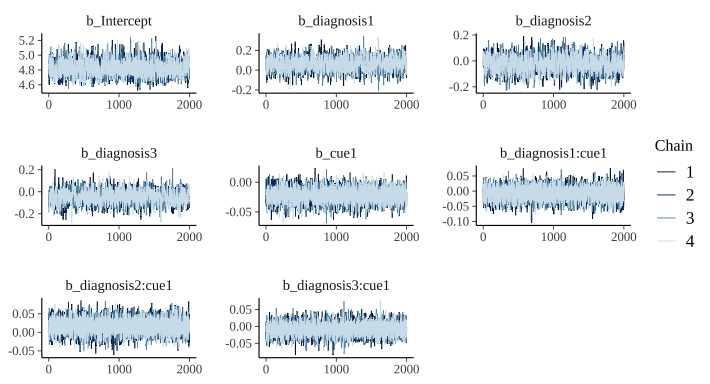


The final model does not exhibit any divergence issues or suboptimal rhats.

# get the posterior predictions
post.pred = posterior_predict(m.lat, ndraws = nsim)

# check the fit of the predicted data compared to the real data
p1 = pp_check(m.lat, ndraws = nsim) +
 theme_bw() + theme(legend.position = "none")

# distributions of means and sds compared to the real values per group
p2 = ppc_stat_grouped(df.lat.agg$lat, post.pred, df.lat.agg$diagnosis) +
 theme_bw() + theme(legend.position = "none")

p = ggarrange(p1, p2,
 nrow = 2, ncol = 1, labels = "AUTO")
annotate_figure(p,
 top = text_grob("Posterior predictive checks: latency",
 face = "bold", size = 14))


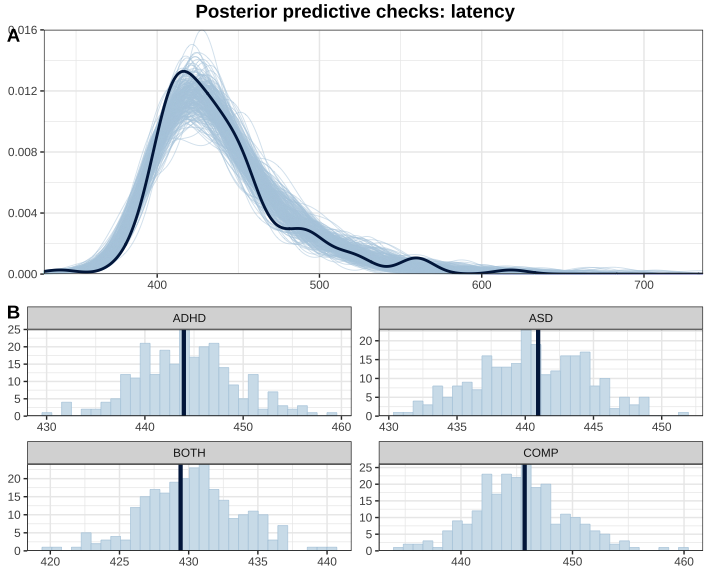


This looks much better with the simulated data based on the model capturing our actual data well.

### 9.2.5 Inferences

Now that we are convinced that we can trust our model, we have a look at the model and its estimates.

# print a summary
summary(m.lat)

## Family: shifted_lognormal
## Links: mu = identity; sigma = identity; ndt = identity
## Formula: lat ~ diagnosis * cue + (1 | subID)
## Data: df.lat.agg (Number of observations: 142)
## Draws: 4 chains, each with iter = 3000; warmup = 1000; thin = 1;
## total post-warmup draws = 8000
##
## Multilevel Hyperparameters:
## ~subID (Number of levels: 75)
## Estimate Est.Error l-95% CI u-95% CI Rhat Bulk_ESS Tail_ESS
## sd(Intercept) 0.30 0.04 0.22 0.40 1.00 1256 1927
##
## Regression Coefficients:
## Estimate Est.Error l-95% CI u-95% CI Rhat Bulk_ESS Tail_ESS
## Intercept 4.82 0.10 4.65 5.04 1.00 1496 2392
## diagnosis1 0.06 0.07 -0.07 0.19 1.00 1253 2101
## diagnosis2 -0.02 0.06 -0.14 0.10 1.00 988 2165
## diagnosis3 -0.05 0.06 -0.16 0.07 1.01 1003 1867
## cue1 -0.02 0.01 -0.05 0.00 1.00 8491 5794
## diagnosis1:cue1 -0.01 0.02 -0.05 0.03 1.00 5273 5549
## diagnosis2:cue1 0.02 0.02 -0.02 0.05 1.00 5909 5217
## diagnosis3:cue1 -0.01 0.02 -0.04 0.03 1.00 6645 5798
##
## Further Distributional Parameters:
## Estimate Est.Error l-95% CI u-95% CI Rhat Bulk_ESS Tail_ESS
## sigma 0.13 0.02 0.10 0.16 1.00 2521 3666
## ndt 308.93 11.12 281.81 325.98 1.00 1724 2856
##
## Draws were sampled using sample(hmc). For each parameter, Bulk_ESS
## and Tail_ESS are effective sample size measures, and Rhat is the potential
## scale reduction factor on split chains (at convergence, Rhat = 1).

# plot the posterior distributions
as_draws_df(m.lat) %>%
 select(starts_with("b_")) %>%
 mutate(
 b_COMP = - b_diagnosis1 - b_diagnosis2 - b_diagnosis3
 ) %>%
 pivot_longer(cols = starts_with("b_"), names_to = "coef", values_to = "estimate") %>%
 filter(coef != "b_Intercept") %>%
 mutate(
 coef = case_match(coef,
 "b_cue1" ~ "Face",
 "b_diagnosis1" ~ "ADHD",
 "b_diagnosis2" ~ "ASD",
 "b_diagnosis3" ~ "ADHD+ASD",
 "b_COMP" ~ "COMP",
 "b_diagnosis1:cue1" ~ "Interaction: ADHD x cue",
 "b_diagnosis2:cue1" ~ "Interaction: ASD x cue",
 "b_diagnosis3:cue1" ~ "Interaction: ADHD+ASD x cue"
 ),
 coef = fct_reorder(coef, desc(estimate))
 ) %>%
 group_by(coef) %>%
 mutate(
 cred = case_when(
 (mean(estimate) < 0 & quantile(estimate, probs = 0.975) < 0) |
 (mean(estimate) > 0 & quantile(estimate, probs = 0.025) > 0) ~ "credible",
 T ~ "not credible"
 )
 ) %>% ungroup() %>%
 ggplot(aes(x = estimate, y = coef, fill = cred)) +
 geom_vline(xintercept = 0, linetype = 'dashed') +
 ggdist::stat_halfeye(alpha = 0.7) + ylab(NULL) + theme_bw() +
 scale_fill_manual(values = c(credible = c_dark, c_light)) +
 theme(legend.position = "none")


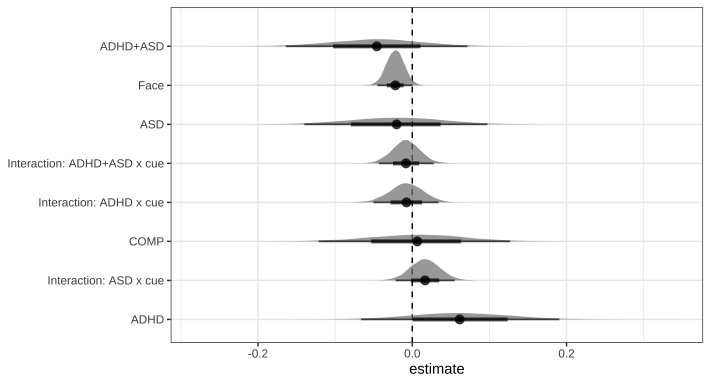


# H2b: ASD(face) > COMP(face)
h2b = hypothesis(m.lat,
 "0 < diagnosis1 + diagnosis3 + 2*diagnosis2 +
 diagnosis1:cue1 + diagnosis3:cue1 + 2*diagnosis2:cue1")
h2b

## Hypothesis Tests for class b:
## Hypothesis Estimate Est.Error CI.Lower CI.Upper Evid.Ratio
## 1 (0)-(diagnosis1+d... < 0 0.01 0.11 -0.17 0.18 0.85
## Post.Prob Star
## 1 0.46
## ---
## 'CI': 90%-CI for one-sided and 95%-CI for two-sided hypotheses.
## '*': For one-sided hypotheses, the posterior probability exceeds 95%;
## for two-sided hypotheses, the value tested against lies outside the 95%-CI.
## Posterior probabilities of point hypotheses assume equal prior probabilities.

# explore: overall faster towards face-cued targets
e = hypothesis(m.lat, "0 > 2*cue1", alpha = 0.025)
e

## Hypothesis Tests for class b:
## Hypothesis Estimate Est.Error CI.Lower CI.Upper Evid.Ratio Post.Prob
## 1 (0)-(2*cue1) > 0 0.04 0.02 0 0.09 36.74 0.97
## Star
## 1
## ---
## 'CI': 95%-CI for one-sided and 97.5%-CI for two-sided hypotheses.
## '*': For one-sided hypotheses, the posterior probability exceeds 97.5%;
## for two-sided hypotheses, the value tested against lies outside the 97.5%-CI.
## Posterior probabilities of point hypotheses assume equal prior probabilities.

# extract predicted differences
df.new = df.lat %>%
 select(diagnosis, cue) %>%
 distinct() %>%
 mutate(
 condition = paste(diagnosis, cue, sep = "_")
 )
df.ms = as.data.frame(
 fitted(m.lat, summary = F,
 newdata = df.new %>% select(diagnosis, cue),
 re_formula = NA))
colnames(df.ms) = df.new$condition

st(df.ms,
 summ = c('mean(x)','sd(x)','min(x)','pctile(x)[2.5]',
 'pctile(x)[97.5]','max(x)'))

Summary Statistics

| Variable | Mean | Sd | Min | Pctile[2.5] | Pctile[97.5] | Max |
| --- | --- | --- | --- | --- | --- | --- |
| COMP_face | 433 | 9.2 | 401 | 415 | 451 | 474 |
| COMP_object | 439 | 9.6 | 408 | 420 | 458 | 478 |
| ADHD_face | 439 | 11 | 405 | 419 | 460 | 490 |
| ADHD_object | 447 | 11 | 409 | 425 | 470 | 501 |
| ASD_face | 432 | 9.1 | 397 | 414 | 450 | 470 |
| ASD_object | 433 | 9.3 | 398 | 415 | 452 | 470 |
| BOTH_face | 426 | 8.1 | 400 | 410 | 442 | 461 |
| BOTH_object | 433 | 8.5 | 405 | 417 | 450 | 474 |

# calculate our difference columns
df.ms = df.ms %>%
 mutate(
 e = rowMeans(select(., matches(".*_object")), na.rm = T) -
 rowMeans(select(., matches(".*_face")), na.rm = T)
 )

st(df.ms %>%
 mutate(
 # get the face and object latencies -200, so they start with target onset
 face = rowMeans(select(., matches(".*_face")), na.rm = T) - 200,
 object = rowMeans(select(., matches(".*_object")), na.rm = T) - 200,
 FAB = object - face
 ) %>% select(face, object, FAB),
 summ = c('mean(x)','sd(x)','min(x)','pctile(x)[2.5]','pctile(x)[97.5]','max(x)'))

Summary Statistics

| Variable | Mean | Sd | Min | Pctile[2.5] | Pctile[97.5] | Max |
| --- | --- | --- | --- | --- | --- | --- |
| face | 233 | 4.9 | 214 | 223 | 242 | 253 |
| object | 238 | 5.1 | 220 | 228 | 248 | 257 |
| FAB | 5.6 | 2.9 | -6 | -0.36 | 11 | 16 |

Our hypothesis that target-elicited saccades towards the faces have a longer latency in ASD than COMP adults was not confirmed by the data (*estimate* = 0.01 [-0.17, 0.18], *posterior probability* = 45.93%). Our exploration revealed that that latencies of target-elicited saccades where faster in response to face-cued compared to object-cued targets regardless of the diagnostic group (*estimate* = 0.04 [0, 0.09], *posterior probability* = 97.35%). Specifically, the model predicted a 5.55ms [-0.1, 11.1] shorter latency of for saccades produced towards a face-cued compared to an object-cued target.

### 9.2.6 Plots

# rain cloud plot for the
p = df.lat.agg %>%
 mutate(
 diagnosis = recode(diagnosis, "BOTH" = "ADHD+ASD"),
 Target = recode(cue, "face" = "Face-cued", "object" = "Object-cued")
 ) %>%
 ggplot(aes(diagnosis, lat, fill = Target, colour = Target)) + #
 geom_rain(rain.side = 'r',
boxplot.args = list(color = "black", outlier.shape = NA, show_guide = FALSE, alpha = .8),
violin.args = list(color = "black", outlier.shape = NA, alpha = .8),
boxplot.args.pos = list(
 position = ggpp::position_dodgenudge(x = 0, width = 0.3), width = 0.3
),
point.args = list(show_guide = FALSE, alpha = .5),
violin.args.pos = list(
 width = 0.6, position = position_nudge(x = 0.16)),
point.args.pos = list(position = ggpp::position_dodgenudge(x = -0.25, width = 0.1))) +
 scale_fill_manual(values = custom.col2) +
 scale_color_manual(values = custom.col2) +
 labs(title = "Latencies of target-elicited saccades", x = "", y = "Latency (ms)") +
 theme_bw() + ylim(300, 625) +
 theme(legend.position = "inside", legend.position.inside = c(0.5, 0.1),
 plot.title = element_text(hjust = 0.5),
 legend.direction = "horizontal",
 legend.title=element_blank(), axis.title.y = element_text(size = 28),
 axis.text.x = element_text(size = 28, colour = "black"),
 axis.text.y = element_text(size = 24),
 text = element_text(family = "Merriweather", size = 32))

ggsave("Fig6_latSac.tif", plot = p,
 units = "mm",
 width = 170,
 height = 100,
 dpi = 300)

# 10 Correlation with reaction times: number of saccades

Last, we hypothesised that the FAB effect on reaction times may be associated with saccades produced towards the face. To investigate this, we use a Bayesian Spearman correlation as both FAB effect and number of saccades are not normally distributed.

# only keep saccades towards faces
df.diff = df.cnt %>% filter(direction == "face")

# check the distribution plot > not normally distributed
p1 = ggplot(df.diff, aes(sample = n.sac)) +
 stat_qq(alpha = 0.75, colour = c_mid_highlight) +
 stat_qq_line() +
 theme_bw()
p2 = ggplot(df.diff, aes(sample = fab)) +
 stat_qq(alpha = 0.75, colour = c_mid_highlight) +
 stat_qq_line() +
 theme_bw()
ggarrange(p1, p2,
 nrow = 1, ncol = 2, labels = "AUTO")


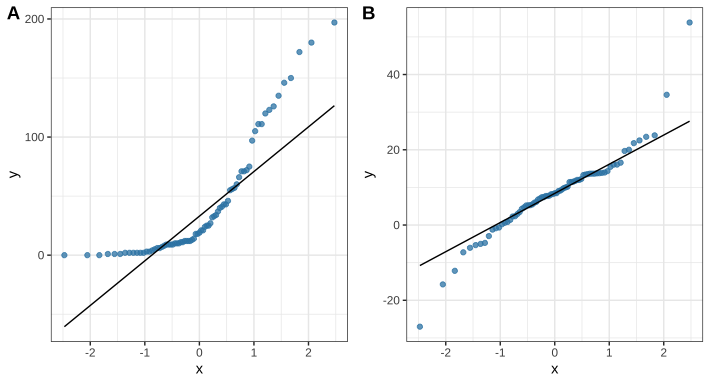


# do a Bayesian Spearman correlation: https://osf.io/j5wud
source("./helpers/rankBasedCommonFunctions.R")
source("./helpers/spearmanSampler.R")

# Default beta prior width is set to a = b = 1 for the sampler
if (file.exists("rho_CNT.rds")) {
 rhoSamples.cnt = readRDS("rho_CNT.rds")
} else {
 set.seed(1597)
 rhoSamples.cnt =
 spearmanGibbsSampler(xVals = df.diff$n.sac,
 yVals = df.diff$fab,
 nSamples = 5e3)
 saveRDS(rhoSamples.cnt, file = "rho_CNT.rds")
}

# give the posterior samples for rho to the function below to compute BF01
cor.bf = computeBayesFactorOneZero(rhoSamples.cnt$rhoSamples,
 whichTest = "Spearman",
 priorParameter = 1)

# visualise it
ggplot(data = df.diff, aes(x = n.sac, y = fab)) +
 geom_point(colour = c_mid_highlight) +
 geom_smooth(method = "lm",
 formula = y ~ x,
 geom = "smooth", colour = c_dark_highlight) +
 theme_bw()


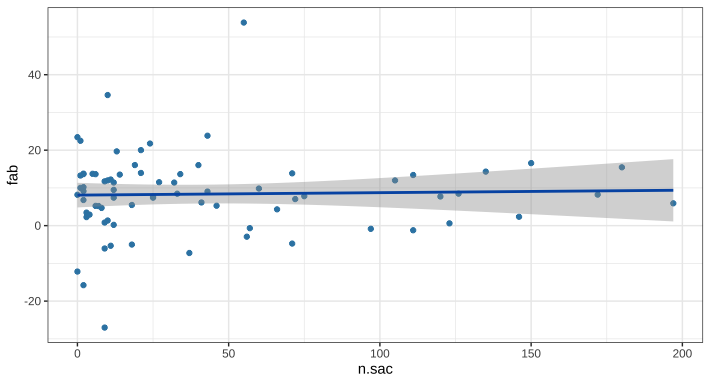


Furthermore, we assessed the relationship between FAB and number of saccades produced towards the face on the participant level (see supplementary materials S2.4). We used a Bayesian Spearman correlation due to both values not being normally distributed. This model revealed no association between number of saccades and face attention bias, in fact there was moderate evidence against an association between the number of saccades and face attention bias (log(*BF*) = -1.95).

# 11 Exploration: cue-elicited saccades

Additionally to our hypotheses, we also explored any effects of diagnostic status, cue type and their interaction on whether a saccade was produced during the presentation of the cues to assess the findings of increased saccade frequency towards faces by Pereira et al. (2020)in our data. Since these are again count data, we will use a Poisson and then compare the overall descriptives and effects to Pereira and colleague’s results. Pereira found about 6% of trials contained cue-elicited saccades which translates to an intercept of 3. Therefore, we can use the same priors and SBC as in our Poisson investigating numbers of saccades in general.

# aggregate to counts
df.cnt.cue = df.cue %>%
 group_by(subID, diagnosis) %>%
 summarise(
 face = sum(direction == "face", na.rm = T),
 object = sum(direction == "object", na.rm = T)
 ) %>%
 pivot_longer(cols = c(face, object), names_to = "direction", values_to = "n.sac") %>%
 mutate_if(is.character, as.factor)

# aggregate for descriptives over both conditions
df.cnt.cue.agg = df.cnt.cue %>% group_by(subID, diagnosis) %>%
 summarise(n.sac = sum(n.sac))

# set the contrasts
contrasts(df.cnt.cue$direction) = contr.sum(2)
contrasts(df.cnt.cue$direction)

## [,1]
## face 1
## object -1

contrasts(df.cnt.cue$diagnosis) = contr.sum(4)
contrasts(df.cnt.cue$diagnosis)

## [,1] [,2] [,3]
## ADHD 1 0 0
## ASD 0 1 0
## BOTH 0 0 1
## COMP -1 -1 -1

# set the same formula
f.cnt = brms::bf(n.sac ~ diagnosis * direction + (1 | subID))

# set priors based on study design
priors = c(
 prior(normal(3, 1.5), class = Intercept),
 prior(normal(0, 1.0), class = sd),
 prior(normal(0, 1.0), class = b)
)

# set number of iterations and warmup for models
iter = 4500
warm = 1500

## 11.1 Posterior predictive checks

As the next step, we fit the model and check whether the chains have converged, which they seem to have. We then perform posterior predictive checks on the model using the bayesplot package.

# fit the model
set.seed(4682)
m.cnt = brm(f.cnt,
 df.cnt.cue, prior = priors,
 iter = iter, warmup = warm,
 backend = "cmdstanr", threads = threading(8),
 file = "m_cnt-cue",
 family = "poisson",
 save_pars = save_pars(all = TRUE)
 )
rstan::check_hmc_diagnostics(m.cnt$fit)

##
## Divergences:

## 0 of 12000 iterations ended with a divergence.

##
## Tree depth:

## 0 of 12000 iterations saturated the maximum tree depth of 10.

##
## Energy:

## E-BFMI indicated no pathological behavior.

# check that rhats are below 1.01
sum(brms::rhat(m.cnt) >= 1.01, na.rm = T)

## [1] 0

# check the trace plots
post.draws = as_draws_df(m.cnt)
mcmc_trace(post.draws, regex_pars = "^b_",
 facet_args = list(ncol = 3)) +
 scale_x_continuous(breaks=scales::pretty_breaks(n = 3)) +
 scale_y_continuous(breaks=scales::pretty_breaks(n = 3))

## Scale for x is already present.
## Adding another scale for x, which will replace the existing scale.


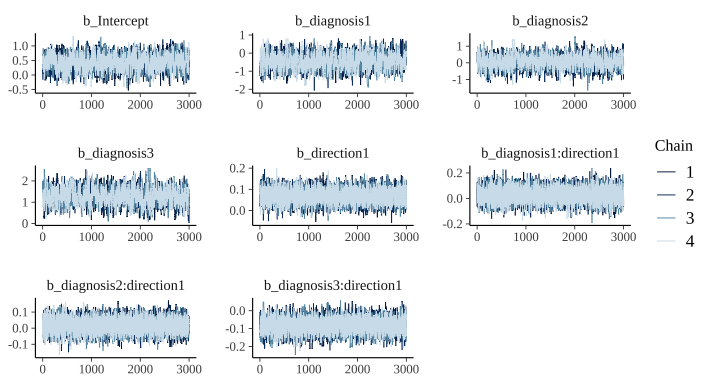


This model has no divergent samples and no rhats that are higher or equal to 1.01. Therefore, we go ahead and perform our posterior predictive checks.

# get the posterior predictions
post.pred = posterior_predict(m.cnt, ndraws = nsim)

# check the fit of the predicted data compared to the real data
p1 = pp_check(m.cnt, ndraws = nsim) +
 theme_bw()

# distributions of means and sds compared to the real values per group
p2 = ppc_stat_grouped(df.cnt.cue$n.sac, post.pred, df.cnt.cue$diagnosis) +
 theme_bw()

p = ggarrange(p1, p2,
 nrow = 2, ncol = 1, labels = "AUTO")
annotate_figure(p,
 top = text_grob("Posterior predictive checks: number of cue-elicited saccades",
 face = "bold", size = 14))


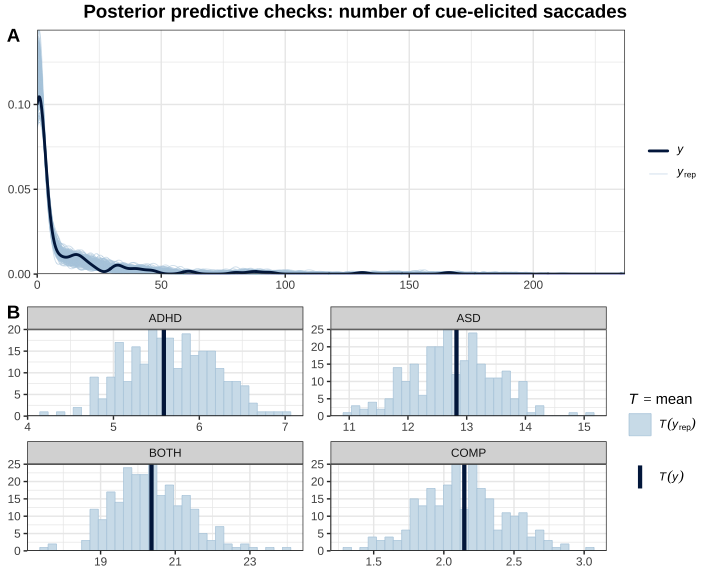


The predictions based on the model capture the data well. This further increases our trust in the model.

## 11.2 Inferences

Now that we are convinced that we can trust our model, we have a look at the model and its estimates.

# print a summary
summary(m.cnt)

## Family: poisson
## Links: mu = log
## Formula: n.sac ~ diagnosis * direction + (1 | subID)
## Data: df.cnt.cue (Number of observations: 184)
## Draws: 4 chains, each with iter = 4500; warmup = 1500; thin = 1;
## total post-warmup draws = 12000
##
## Multilevel Hyperparameters:
## ~subID (Number of levels: 92)
## Estimate Est.Error l-95% CI u-95% CI Rhat Bulk_ESS Tail_ESS
## sd(Intercept) 2.08 0.21 1.71 2.52 1.00 1777 4082
##
## Regression Coefficients:
## Estimate Est.Error l-95% CI u-95% CI Rhat Bulk_ESS
## Intercept 0.45 0.25 -0.06 0.92 1.00 1456
## diagnosis1 -0.43 0.39 -1.21 0.35 1.00 1635
## diagnosis2 0.05 0.37 -0.69 0.79 1.01 1456
## diagnosis3 1.29 0.36 0.58 1.98 1.00 1063
## direction1 0.07 0.03 0.01 0.13 1.00 6981
## diagnosis1:direction1 0.03 0.06 -0.08 0.14 1.00 11505
## diagnosis2:direction1 0.02 0.04 -0.07 0.10 1.00 9677
## diagnosis3:direction1 -0.08 0.04 -0.16 -0.01 1.00 7276
## Tail_ESS
## Intercept 2652
## diagnosis1 2945
## diagnosis2 2552
## diagnosis3 2189
## direction1 8164
## diagnosis1:direction1 9294
## diagnosis2:direction1 9112
## diagnosis3:direction1 8565
##
## Draws were sampled using sample(hmc). For each parameter, Bulk_ESS
## and Tail_ESS are effective sample size measures, and Rhat is the potential
## scale reduction factor on split chains (at convergence, Rhat = 1).

# get the estimates and compute groups
df.m.cnt = as_draws_df(m.cnt) %>%
 select(starts_with("b_")) %>%
 mutate(
 b_COMP = - b_diagnosis1 - b_diagnosis2 - b_diagnosis3,
 ASD = b_Intercept + b_diagnosis2,
 ADHD = b_Intercept + b_diagnosis1,
 BOTH = b_Intercept + b_diagnosis3,
 COMP = b_Intercept + b_COMP
 )

# plot the posterior distributions
df.m.cnt %>%
 select(starts_with("b_")) %>%
 pivot_longer(cols = starts_with("b_"), names_to = "coef", values_to = "estimate") %>%
 filter(coef != "b_Intercept") %>%
 mutate(
 coef = case_match(coef,
 "b_diagnosis1" ~ "ADHD",
 "b_diagnosis2" ~ "ASD",
 "b_diagnosis3" ~ "ADHD+ASD",
 "b_COMP" ~ "COMP",
 "b_direction1" ~ "Face",
 "b_diagnosis1:direction1" ~ "Interaction: ADHD",
 "b_diagnosis2:direction1" ~ "Interaction: ASD",
 "b_diagnosis3:direction1" ~ "Interaction: ADHD+ASD"
 ),
 coef = fct_reorder(coef, desc(estimate))
 ) %>%
 group_by(coef) %>%
 mutate(
 cred = case_when(
 (mean(estimate) < 0 & quantile(estimate, probs = 0.975) < 0) |
 (mean(estimate) > 0 & quantile(estimate, probs = 0.025) > 0) ~ "credible",
 T ~ "not credible"
 )
 ) %>% ungroup() %>%
 ggplot(aes(x = estimate, y = coef, fill = cred)) +
 geom_vline(xintercept = 0, linetype = 'dashed') +
 ggdist::stat_halfeye(alpha = 0.7) + ylab(NULL) + theme_bw() +
 scale_fill_manual(values = c(credible = c_dark, c_light)) +
 theme(legend.position = "none")


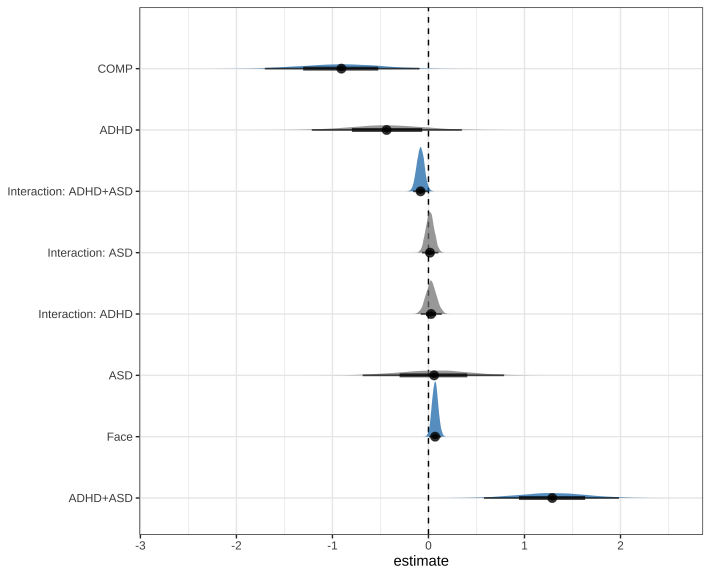


# face > object
e = hypothesis(m.cnt, "0 < 2*(direction1)",
 alpha = 0.025)
e

## Hypothesis Tests for class b:
## Hypothesis Estimate Est.Error CI.Lower CI.Upper Evid.Ratio
## 1 (0)-(2*(direction1)) < 0 -0.14 0.06 -0.27 -0.02 68.36
## Post.Prob Star
## 1 0.99 *
## ---
## 'CI': 95%-CI for one-sided and 97.5%-CI for two-sided hypotheses.
## '*': For one-sided hypotheses, the posterior probability exceeds 97.5%;
## for two-sided hypotheses, the value tested against lies outside the 97.5%-CI.
## Posterior probabilities of point hypotheses assume equal prior probabilities.

# extract predicted differences
df.new = df.cnt.cue %>% ungroup() %>%
 select(diagnosis, direction) %>%
 distinct() %>%
 mutate(
 condition = paste(diagnosis, direction, sep = "_")
 )
df.ms = as.data.frame(
 fitted(m.cnt, summary = F,
 newdata = df.new %>% select(diagnosis, direction),
 re_formula = NA))
colnames(df.ms) = df.new$condition

st(df.ms,
 summ = c('mean(x)','sd(x)','min(x)','pctile(x)[2.5]',
 'pctile(x)[97.5]','max(x)'))

Summary Statistics

| Variable | Mean | Sd | Min | Pctile[2.5] | Pctile[97.5] | Max |
| --- | --- | --- | --- | --- | --- | --- |
| ADHD_face | 1.3 | 0.63 | 0.17 | 0.41 | 2.7 | 6.3 |
| ADHD_object | 1 | 0.52 | 0.12 | 0.34 | 2.3 | 5.4 |
| COMP_face | 0.79 | 0.41 | 0.093 | 0.25 | 1.7 | 4.4 |
| COMP_object | 0.64 | 0.33 | 0.074 | 0.2 | 1.4 | 3.6 |
| ASD_face | 2 | 0.9 | 0.21 | 0.71 | 4 | 9.9 |
| ASD_object | 1.7 | 0.76 | 0.2 | 0.59 | 3.4 | 8.4 |
| BOTH_face | 6.1 | 2.7 | 1.2 | 2.3 | 12 | 26 |
| BOTH_object | 6.3 | 2.8 | 1.3 | 2.4 | 13 | 29 |

st(df.ms %>%
 mutate(
 face = rowMeans(select(., matches(".*_face")), na.rm = T),
 object = rowMeans(select(., matches(".*_object")), na.rm = T),
 FAB = object - face
 ) %>% select(face, object, FAB),
 summ = c('mean(x)','sd(x)','min(x)','pctile(x)[2.5]','pctile(x)[97.5]','max(x)'))

Summary Statistics

| Variable | Mean | Sd | Min | Pctile[2.5] | Pctile[97.5] | Max |
| --- | --- | --- | --- | --- | --- | --- |
| face | 2.5 | 0.77 | 0.77 | 1.3 | 4.3 | 7.5 |
| object | 2.4 | 0.76 | 0.75 | 1.2 | 4.2 | 8 |
| FAB | -0.13 | 0.14 | -0.84 | -0.44 | 0.14 | 0.66 |

On average, participants produced cue-elicited saccades on 4.64% +- 1.06 of the trials. However, the range was very wide, with some participants producing none and others producing them on 68.75% of the trials. Regardless of group, credibly more cue-elicited saccades were produced towards face compared to object cues (*estimate* = -0.14 [-0.27, -0.02], *posterior probability* = 98.56%).

## 11.3 Plots

As a last step, we can plot our data.

# rain cloud plot
df.cnt %>%
 mutate(
 diagnosis = recode(diagnosis, "BOTH" = "ADHD+ASD")
 ) %>%
 ggplot(aes(diagnosis, n.sac, fill = direction, colour = direction)) + #
 geom_rain(rain.side = 'r',
boxplot.args = list(color = "black", outlier.shape = NA, show_guide = FALSE, alpha = .8),
violin.args = list(color = "black", outlier.shape = NA, alpha = .8),
boxplot.args.pos = list(
 position = ggpp::position_dodgenudge(x = 0, width = 0.3), width = 0.3
),
point.args = list(show_guide = FALSE, alpha = .5),
violin.args.pos = list(
 width = 0.6, position = position_nudge(x = 0.16)),
point.args.pos = list(position = ggpp::position_dodgenudge(x = -0.25, width = 0.1))) +
 scale_fill_manual(values = custom.col2) +
 scale_color_manual(values = custom.col2) +
 labs(title = "Number of cue-elicited saccades", x = "", y = "n") +
 theme_bw() +
 theme(legend.position = "bottom",
 plot.title = element_text(hjust = 0.5),
 legend.direction = "horizontal",
 text = element_text(family = "Merriweather", size = 15))


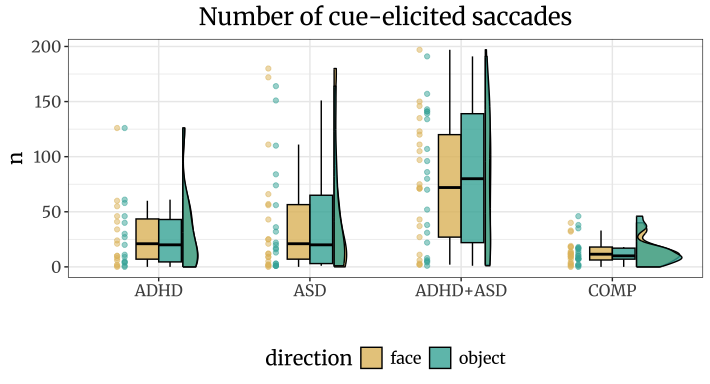


# 12 Exploration: Latencies of cue-elicited saccades

## 12.1 Model

We assume that the SBC for the hypothesis-guided latency analysis holds for this. We only need to slightly adjust the prior for the shift. This analysis only includes participants who performed cue-elicited saccades.

# preprocess cue latencies
df.lat.cue = df.cue %>%
 filter(!is.na(direction)) %>%
 group_by(subID, direction, diagnosis) %>%
 summarise(lat = median(lat, na.rm = T)) %>%
 mutate_if(is.character, as.factor)

# set the formula
f.lat = brms::bf(lat ~ diagnosis * direction + (1 | subID) )

# set weakly informative priors
priors = c(
 prior(normal(5, 0.75), class = Intercept),
 prior(normal(0, 0.25), class = sd),
 prior(normal(0, 0.25), class = b),
 prior(normal(0.5, 0.50), class = sigma),
 prior(normal(150, 50.00), class = ndt) # this is the only prior that differs
)

# set number of iterations and warmup for models
iter = 3000
warm = 1000

# set the contrasts
contrasts(df.lat.cue$direction) = contr.sum(2)
contrasts(df.lat.cue$direction)

## [,1]
## face 1
## object -1

contrasts(df.lat.cue$diagnosis) = contr.sum(4)
contrasts(df.lat.cue$diagnosis)

## [,1] [,2] [,3]
## ADHD 1 0 0
## ASD 0 1 0
## BOTH 0 0 1
## COMP -1 -1 -1

# fit the maximal model
set.seed(7799)
m.lat = brm(f.lat,
 df.lat.cue, prior = priors,
 iter = iter, warmup = warm,
 backend = "cmdstanr", threads = threading(8),
 family = "shifted_lognormal",
 file = "m_lat-cue_agg",
 save_pars = save_pars(all = TRUE)
 )
rstan::check_hmc_diagnostics(m.lat$fit)

##
## Divergences:

## 0 of 8000 iterations ended with a divergence.

##
## Tree depth:

## 0 of 8000 iterations saturated the maximum tree depth of 10.

##
## Energy:

## E-BFMI indicated no pathological behavior.

# check that rhats are below 1.01
sum(brms::rhat(m.lat) >= 1.01, na.rm = T)

## [1] 0

# check the trace plots
post.draws = as_draws_df(m.lat)
mcmc_trace(post.draws, regex_pars = "^b_",
 facet_args = list(ncol = 3)) +
 scale_x_continuous(breaks=scales::pretty_breaks(n = 3)) +
 scale_y_continuous(breaks=scales::pretty_breaks(n = 3))

## Scale for x is already present.
## Adding another scale for x, which will replace the existing scale.


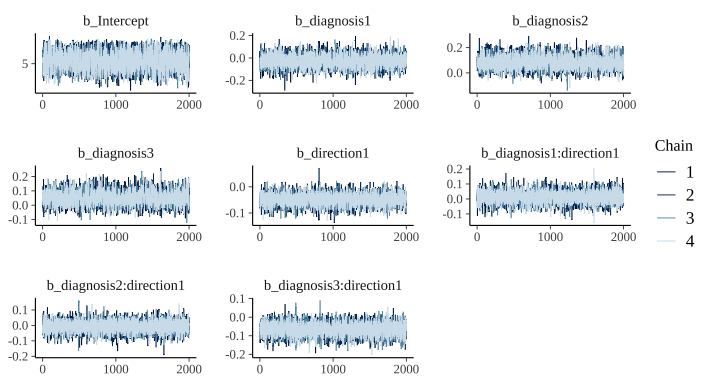


The final model does not exhibit any divergence issues or suboptimal rhats.

# get the posterior predictions
post.pred = posterior_predict(m.lat, ndraws = nsim)

# check the fit of the predicted data compared to the real data
p1 = pp_check(m.lat, ndraws = nsim) +
 theme_bw() + theme(legend.position = "none")

# distributions of means and sds compared to the real values per group
p2 = ppc_stat_grouped(df.lat.cue$lat, post.pred, df.lat.cue$diagnosis) +
 theme_bw() + theme(legend.position = "none")

p = ggarrange(p1, p2,
 nrow = 2, ncol = 1, labels = "AUTO")
annotate_figure(p,
 top = text_grob("Posterior predictive checks: latency cues",
 face = "bold", size = 14))


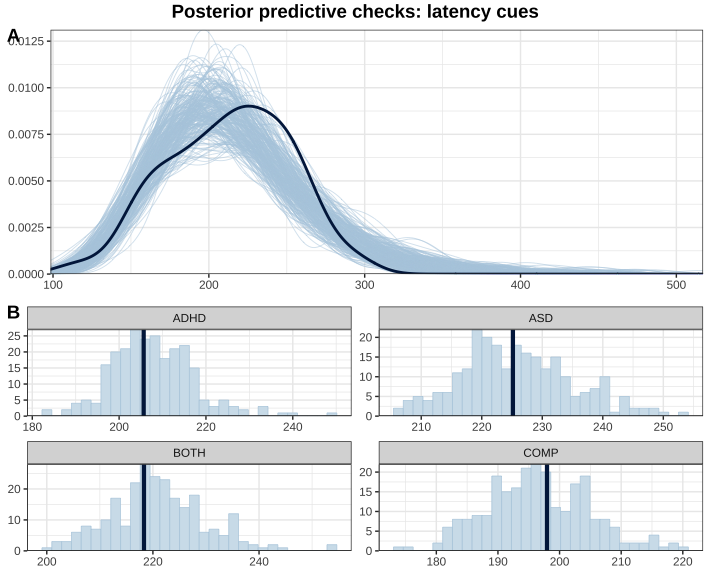


This looks good.

## 12.2 Inferences

Now that we are convinced that we can trust our model, we have a look at the model and its estimates.

# print a summary
summary(m.lat)

## Family: shifted_lognormal
## Links: mu = identity; sigma = identity; ndt = identity
## Formula: lat ~ diagnosis * direction + (1 | subID)
## Data: df.lat.cue (Number of observations: 114)
## Draws: 4 chains, each with iter = 3000; warmup = 1000; thin = 1;
## total post-warmup draws = 8000
##
## Multilevel Hyperparameters:
## ~subID (Number of levels: 63)
## Estimate Est.Error l-95% CI u-95% CI Rhat Bulk_ESS Tail_ESS
## sd(Intercept) 0.16 0.05 0.05 0.25 1.01 1185 1095
##
## Regression Coefficients:
## Estimate Est.Error l-95% CI u-95% CI Rhat Bulk_ESS
## Intercept 5.05 0.13 4.80 5.29 1.00 3536
## diagnosis1 -0.03 0.05 -0.13 0.08 1.00 4200
## diagnosis2 0.08 0.05 -0.01 0.19 1.00 4853
## diagnosis3 0.05 0.05 -0.04 0.14 1.00 3921
## direction1 -0.05 0.02 -0.09 -0.01 1.00 8019
## diagnosis1:direction1 0.01 0.04 -0.06 0.09 1.00 8175
## diagnosis2:direction1 -0.01 0.04 -0.08 0.06 1.00 8286
## diagnosis3:direction1 -0.06 0.03 -0.13 -0.00 1.00 7929
## Tail_ESS
## Intercept 3811
## diagnosis1 4340
## diagnosis2 5316
## diagnosis3 4582
## direction1 5722
## diagnosis1:direction1 5645
## diagnosis2:direction1 4819
## diagnosis3:direction1 5779
##
## Further Distributional Parameters:
## Estimate Est.Error l-95% CI u-95% CI Rhat Bulk_ESS Tail_ESS
## sigma 0.21 0.04 0.15 0.30 1.00 2247 3746
## ndt 49.00 18.65 9.25 81.18 1.00 3620 3566
##
## Draws were sampled using sample(hmc). For each parameter, Bulk_ESS
## and Tail_ESS are effective sample size measures, and Rhat is the potential
## scale reduction factor on split chains (at convergence, Rhat = 1).

# plot the posterior distributions
as_draws_df(m.lat) %>%
 select(starts_with("b_")) %>%
 mutate(
 b_COMP = - b_diagnosis1 - b_diagnosis2 - b_diagnosis3
 ) %>%
 pivot_longer(cols = starts_with("b_"), names_to = "coef", values_to = "estimate") %>%
 filter(coef != "b_Intercept") %>%
 mutate(
 coef = case_match(coef,
 "b_direction1" ~ "Face",
 "b_diagnosis1" ~ "ADHD",
 "b_diagnosis2" ~ "ASD",
 "b_diagnosis3" ~ "ADHD+ASD",
 "b_COMP" ~ "COMP",
 "b_diagnosis1:direction1" ~ "Interaction: ADHD x direction",
 "b_diagnosis2:direction1" ~ "Interaction: ASD x direction",
 "b_diagnosis3:direction1" ~ "Interaction: ADHD+ASD x direction"
 ),
 coef = fct_reorder(coef, desc(estimate))
 ) %>%
 group_by(coef) %>%
 mutate(
 cred = case_when(
 (mean(estimate) < 0 & quantile(estimate, probs = 0.975) < 0) |
 (mean(estimate) > 0 & quantile(estimate, probs = 0.025) > 0) ~ "credible",
 T ~ "not credible"
 )
 ) %>% ungroup() %>%
 ggplot(aes(x = estimate, y = coef, fill = cred)) +
 geom_vline(xintercept = 0, linetype = 'dashed') +
 ggdist::stat_halfeye(alpha = 0.7) + ylab(NULL) + theme_bw() +
 scale_fill_manual(values = c(credible = c_dark, c_light)) +
 theme(legend.position = "none")


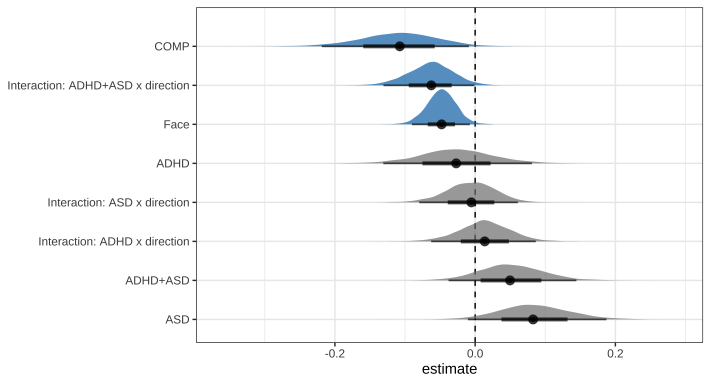


# explore: faster towards faces
e = hypothesis(m.lat, "0 > 2*direction1", alpha = 0.025)
e

## Hypothesis Tests for class b:
## Hypothesis Estimate Est.Error CI.Lower CI.Upper Evid.Ratio
## 1 (0)-(2*direction1) > 0 0.1 0.04 0.02 0.18 86.91
## Post.Prob Star
## 1 0.99 *
## ---
## 'CI': 95%-CI for one-sided and 97.5%-CI for two-sided hypotheses.
## '*': For one-sided hypotheses, the posterior probability exceeds 97.5%;
## for two-sided hypotheses, the value tested against lies outside the 97.5%-CI.
## Posterior probabilities of point hypotheses assume equal prior probabilities.

# extract predicted differences
df.new = df.lat.cue %>% ungroup() %>%
 select(diagnosis, direction) %>%
 distinct() %>%
 mutate(
 condition = paste(diagnosis, direction, sep = "_")
 )
df.ms = as.data.frame(
 fitted(m.lat, summary = F,
 newdata = df.new %>% select(diagnosis, direction),
 re_formula = NA))
colnames(df.ms) = df.new$condition

st(df.ms,
 summ = c('mean(x)','sd(x)','min(x)','pctile(x)[2.5]',
 'pctile(x)[97.5]','max(x)'))

Summary Statistics

| Variable | Mean | Sd | Min | Pctile[2.5] | Pctile[97.5] | Max |
| --- | --- | --- | --- | --- | --- | --- |
| COMP_face | 194 | 11 | 162 | 174 | 215 | 243 |
| COMP_object | 192 | 10 | 155 | 172 | 213 | 236 |
| ADHD_face | 200 | 11 | 166 | 179 | 222 | 266 |
| ADHD_object | 211 | 13 | 163 | 187 | 236 | 281 |
| ASD_face | 214 | 12 | 171 | 191 | 237 | 265 |
| ASD_object | 233 | 12 | 194 | 209 | 258 | 286 |
| BOTH_face | 200 | 9 | 168 | 182 | 218 | 240 |
| BOTH_object | 237 | 11 | 201 | 215 | 260 | 291 |

# calculate our difference columns
df.ms = df.ms %>%
 mutate(
 e = rowMeans(select(., matches(".*_object")), na.rm = T) -
 rowMeans(select(., matches(".*_face")), na.rm = T)
 )

st(df.ms %>%
 mutate(
 face = rowMeans(select(., matches(".*_face")), na.rm = T),
 object = rowMeans(select(., matches(".*_object")), na.rm = T),
 FAB = e
 ) %>% select(face, object, FAB),
 summ = c('mean(x)','sd(x)','min(x)','pctile(x)[2.5]','pctile(x)[97.5]','max(x)'))

Summary Statistics

| Variable | Mean | Sd | Min | Pctile[2.5] | Pctile[97.5] | Max |
| --- | --- | --- | --- | --- | --- | --- |
| face | 202 | 5.5 | 182 | 191 | 213 | 227 |
| object | 218 | 5.9 | 199 | 207 | 230 | 242 |
| FAB | 16 | 6.5 | -18 | 2.7 | 29 | 40 |

A similar effect was found when exploring the latencies of cue-induced saccade, with participants producing saccades towards face cues faster than saccades towards object cues, independent of group (*estimate* = 0.1 [0.02, 0.18], *posterior probability* = 98.86%). This model predicted that if a cue-induced saccade was produced towards the face, the latency was 16.2ms [3.21, 29.02] shorter than if a saccade was produced towards the object cue.

## 12.3 Plots

# rain cloud plot for the
df.lat.cue %>%
 mutate(
 diagnosis = recode(diagnosis, "BOTH" = "ADHD+ASD")
 ) %>%
 ggplot(aes(diagnosis, lat, fill = direction, colour = direction)) + #
 geom_rain(rain.side = 'r',
boxplot.args = list(color = "black", outlier.shape = NA, show_guide = FALSE, alpha = .8),
violin.args = list(color = "black", outlier.shape = NA, alpha = .8),
boxplot.args.pos = list(
 position = ggpp::position_dodgenudge(x = 0, width = 0.3), width = 0.3
),
point.args = list(show_guide = FALSE, alpha = .5),
violin.args.pos = list(
 width = 0.6, position = position_nudge(x = 0.16)),
point.args.pos = list(position = ggpp::position_dodgenudge(x = -0.25, width = 0.1))) +
 scale_fill_manual(values = custom.col2) +
 scale_color_manual(values = custom.col2) +
 labs(title = "Latencies of cue-elicited saccades", x = "", y = "ms") +
 theme_bw() +
 ylim(0, 325) +
 theme(legend.position = "bottom",
 plot.title = element_text(hjust = 0.5),
 legend.direction = "horizontal",
 text = element_text(family = "Merriweather", size = 15))


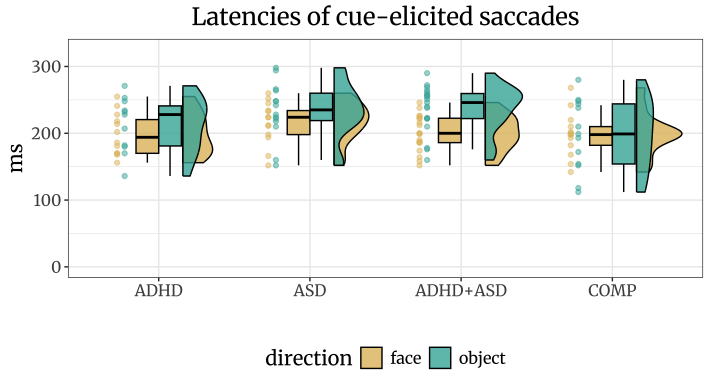


# 13 Exploration: Dwell times starting within cue-elicited saccade window

## 13.1 Model

We assume that the SBC from the reaction time lognormal model holds here, although we slightly widen the Intercept because we have less prior knowledge.

# read in the preprocessed data
df.fix = readRDS("FAB_ET_fix.rds")

# set the formula
f.fix = brms::bf(duration ~ diagnosis * ROI * onTar +
 (ROI * onTar | subID) + (ROI * onTar | subID))

# set weakly informative priors
priors = c(
 # general priors based on SBV
 prior(normal(6, 0.6), class = Intercept),
 prior(normal(0, 0.5), class = sigma),
 prior(normal(0, 0.1), class = sd),
 prior(lkj(2), class = cor),
 prior(normal(0, 0.04), class = b),
 # shift
 prior(normal(200, 100), class = ndt)
)

# set number of iterations and warmup for models
iter = 3000
warm = 1000

# set the contrasts
contrasts(df.fix$ROI) = contr.sum(2)
contrasts(df.fix$ROI)

## [,1]
## face 1
## object -1

contrasts(df.fix$onTar) = contr.sum(2)
contrasts(df.fix$onTar)

## [,1]
## FALSE 1
## TRUE -1

contrasts(df.fix$diagnosis) = contr.sum(4)
contrasts(df.fix$diagnosis)

## [,1] [,2] [,3]
## ADHD 1 0 0
## ASD 0 1 0
## BOTH 0 0 1
## COMP -1 -1 -1

# fit the maximal model
set.seed(5599)
m.fix = brm(f.fix,
 df.fix, prior = priors,
 iter = iter, warmup = warm,
 backend = "cmdstanr", threads = threading(8),
 family = "shifted_lognormal",
 file = "m_fix",
 save_pars = save_pars(all = TRUE)
 )
rstan::check_hmc_diagnostics(m.fix$fit)

##
## Divergences:

## 0 of 8000 iterations ended with a divergence.

##
## Tree depth:

## 0 of 8000 iterations saturated the maximum tree depth of 10.

##
## Energy:

## E-BFMI indicated no pathological behavior.

# check that rhats are below 1.01
sum(brms::rhat(m.fix) >= 1.01, na.rm = T)

## [1] 0

# check the trace plots
post.draws = as_draws_df(m.fix)
mcmc_trace(post.draws, regex_pars = "^b_",
 facet_args = list(ncol = 3)) +
 scale_x_continuous(breaks=scales::pretty_breaks(n = 3)) +
 scale_y_continuous(breaks=scales::pretty_breaks(n = 3))

## Scale for x is already present.
## Adding another scale for x, which will replace the existing scale.


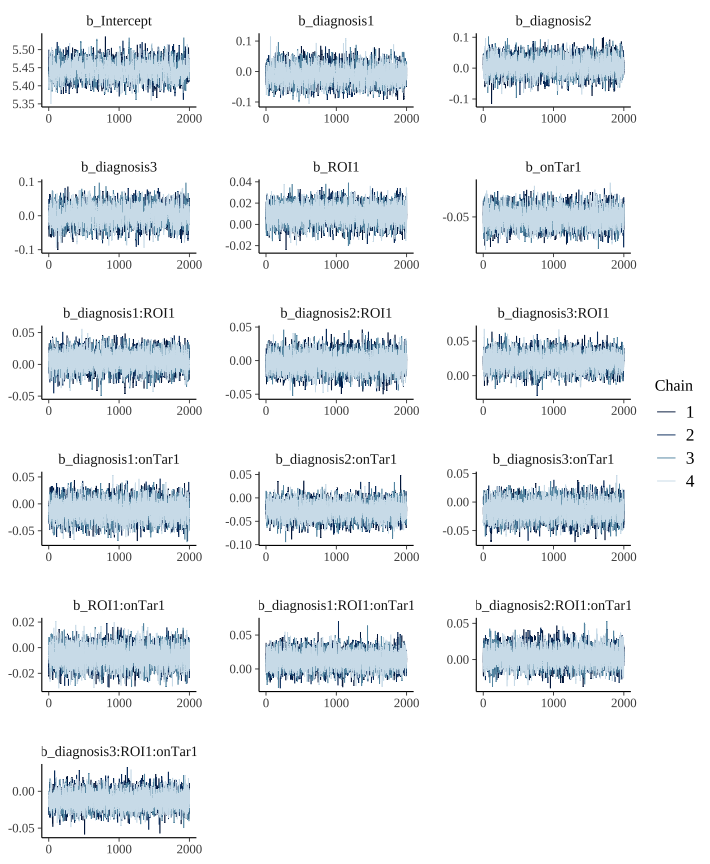


The final model does not exhibit any divergence issues or suboptimal rhats.

# get the posterior predictions
post.pred = posterior_predict(m.fix, ndraws = nsim)

# check the fit of the predicted data compared to the real data
p1 = pp_check(m.fix, ndraws = nsim) +
 theme_bw() + theme(legend.position = "none")

# distributions of means and sds compared to the real values per group
p2 = ppc_stat_grouped(df.fix$duration, post.pred, df.fix$diagnosis) +
 theme_bw() + theme(legend.position = "none")

p = ggarrange(p1, p2,
 nrow = 2, ncol = 1, labels = "AUTO")
annotate_figure(p,
 top = text_grob("Posterior predictive checks: dwell times",
 face = "bold", size = 14))


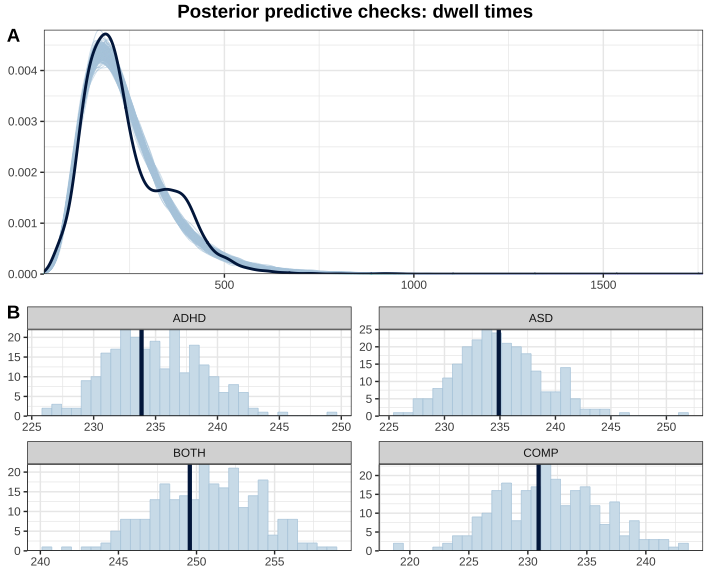


This looks good.

## 13.2 Inferences

Now that we are convinced that we can trust our model, we have a look at the model and its estimates.

# print a summary
summary(m.fix)

## Family: shifted_lognormal
## Links: mu = identity; sigma = identity; ndt = identity
## Formula: duration ~ diagnosis * ROI * onTar + (ROI * onTar | subID) + (ROI * onTar | subID)
## Data: df.fix (Number of observations: 5654)
## Draws: 4 chains, each with iter = 3000; warmup = 1000; thin = 1;
## total post-warmup draws = 8000
##
## Multilevel Hyperparameters:
## ~subID (Number of levels: 78)
## Estimate Est.Error l-95% CI u-95% CI Rhat Bulk_ESS
## sd(Intercept) 0.18 0.02 0.15 0.22 1.00 2868
## sd(ROI1) 0.04 0.01 0.02 0.06 1.00 2493
## sd(onTar1) 0.07 0.01 0.05 0.09 1.00 4102
## sd(ROI1:onTar1) 0.03 0.01 0.00 0.05 1.00 2060
## cor(Intercept,ROI1) -0.27 0.18 -0.61 0.10 1.00 8370
## cor(Intercept,onTar1) 0.19 0.15 -0.10 0.48 1.00 6500
## cor(ROI1,onTar1) 0.16 0.23 -0.29 0.59 1.01 1589
## cor(Intercept,ROI1:onTar1) -0.20 0.24 -0.63 0.29 1.00 10173
## cor(ROI1,ROI1:onTar1) -0.13 0.31 -0.68 0.52 1.00 5599
## cor(onTar1,ROI1:onTar1) -0.39 0.26 -0.81 0.21 1.00 6749
## Tail_ESS
## sd(Intercept) 4641
## sd(ROI1) 2354
## sd(onTar1) 5403
## sd(ROI1:onTar1) 2393
## cor(Intercept,ROI1) 6171
## cor(Intercept,onTar1) 6519
## cor(ROI1,onTar1) 3200
## cor(Intercept,ROI1:onTar1) 5528
## cor(ROI1,ROI1:onTar1) 5340
## cor(onTar1,ROI1:onTar1) 5340
##
## Regression Coefficients:
## Estimate Est.Error l-95% CI u-95% CI Rhat Bulk_ESS
## Intercept 5.44 0.02 5.40 5.49 1.00 2092
## diagnosis1 -0.01 0.03 -0.06 0.05 1.00 3909
## diagnosis2 0.01 0.03 -0.04 0.06 1.00 4609
## diagnosis3 0.00 0.03 -0.05 0.05 1.00 3235
## ROI1 0.01 0.01 -0.01 0.03 1.00 8684
## onTar1 -0.05 0.01 -0.07 -0.03 1.00 7288
## diagnosis1:ROI1 0.01 0.01 -0.02 0.03 1.00 9240
## diagnosis2:ROI1 -0.00 0.01 -0.03 0.02 1.00 10154
## diagnosis3:ROI1 0.02 0.01 -0.00 0.04 1.00 8498
## diagnosis1:onTar1 -0.01 0.02 -0.04 0.02 1.00 7463
## diagnosis2:onTar1 -0.03 0.02 -0.06 0.01 1.00 8029
## diagnosis3:onTar1 -0.01 0.02 -0.04 0.02 1.00 7224
## ROI1:onTar1 -0.01 0.01 -0.02 0.01 1.00 10894
## diagnosis1:ROI1:onTar1 0.01 0.01 -0.01 0.04 1.00 11287
## diagnosis2:ROI1:onTar1 0.00 0.01 -0.02 0.03 1.00 11456
## diagnosis3:ROI1:onTar1 -0.01 0.01 -0.03 0.01 1.00 9056
## Tail_ESS
## Intercept 3278
## diagnosis1 5389
## diagnosis2 5491
## diagnosis3 4788
## ROI1 7010
## onTar1 5965
## diagnosis1:ROI1 6664
## diagnosis2:ROI1 6835
## diagnosis3:ROI1 6945
## diagnosis1:onTar1 6303
## diagnosis2:onTar1 6500
## diagnosis3:onTar1 6376
## ROI1:onTar1 6985
## diagnosis1:ROI1:onTar1 6701
## diagnosis2:ROI1:onTar1 6876
## diagnosis3:ROI1:onTar1 6098
##
## Further Distributional Parameters:
## Estimate Est.Error l-95% CI u-95% CI Rhat Bulk_ESS Tail_ESS
## sigma 0.41 0.00 0.40 0.42 1.00 13572 6037
## ndt 0.59 0.58 0.01 2.15 1.00 8922 4640
##
## Draws were sampled using sample(hmc). For each parameter, Bulk_ESS
## and Tail_ESS are effective sample size measures, and Rhat is the potential
## scale reduction factor on split chains (at convergence, Rhat = 1).

# plot the posterior distributions
as_draws_df(m.fix) %>%
 select(starts_with("b_")) %>%
 mutate(
 b_COMP = - b_diagnosis1 - b_diagnosis2 - b_diagnosis3
 ) %>%
 pivot_longer(cols = starts_with("b_"), names_to = "coef", values_to = "estimate") %>%
 filter(coef != "b_Intercept") %>%
 mutate(
 coef = substr(coef, 3, nchar(coef)),
 coef = str_replace_all(coef, ":", " x "),
 coef = str_replace_all(coef, "ROI1", "FaceROI"),
 coef = str_replace_all(coef, "onTar1", "notTarget"),
 coef = str_replace_all(coef, "diagnosis1", "ADHD"),
 coef = str_replace_all(coef, "diagnosis2", "ASD"),
 coef = str_replace_all(coef, "diagnosis3", "ADHD+ASD"),
 coef = fct_reorder(coef, desc(estimate))
 ) %>%
 group_by(coef) %>%
 mutate(
 cred = case_when(
 (mean(estimate) < 0 & quantile(estimate, probs = 0.975) < 0) |
 (mean(estimate) > 0 & quantile(estimate, probs = 0.025) > 0) ~ "credible",
 T ~ "not credible"
 )
 ) %>% ungroup() %>%
 ggplot(aes(x = estimate, y = coef, fill = cred)) +
 geom_vline(xintercept = 0, linetype = 'dashed') +
 ggdist::stat_halfeye(alpha = 0.7) + ylab(NULL) + theme_bw() +
 scale_fill_manual(values = c(credible = c_dark, c_light)) +
 theme(legend.position = "none")


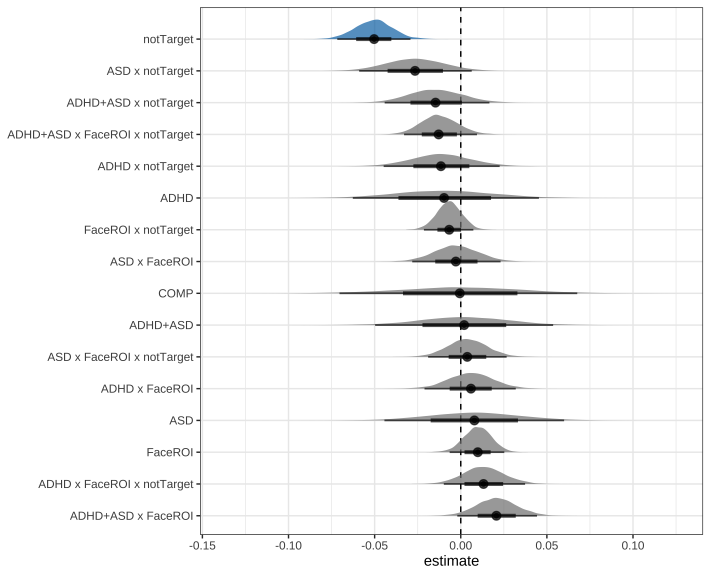


## 13.3 Plots

# rain cloud plot for the
df.fix %>%
 group_by(subID, diagnosis, ROI, onTar) %>%
 summarise(
 duration = median(duration, na.rm = T)
 ) %>%
 mutate(
 diagnosis = recode(diagnosis, "BOTH" = "ADHD+ASD")
 ) %>%
 ggplot(aes(diagnosis, duration, fill = ROI, colour = ROI)) + #
 geom_rain(rain.side = 'r',
boxplot.args = list(color = "black", outlier.shape = NA, show_guide = FALSE, alpha = .8),
violin.args = list(color = "black", outlier.shape = NA, alpha = .8),
boxplot.args.pos = list(
 position = ggpp::position_dodgenudge(x = 0, width = 0.3), width = 0.3
),
point.args = list(show_guide = FALSE, alpha = .5),
violin.args.pos = list(
 width = 0.6, position = position_nudge(x = 0.16)),
point.args.pos = list(position = ggpp::position_dodgenudge(x = -0.25, width = 0.1))) +
 scale_fill_manual(values = custom.col2) +
 scale_color_manual(values = custom.col2) +
 labs(title = "Dwell times starting during cue-elicited saccades", x = "", y = "") +
 theme_bw() +
 facet_wrap(. ~ onTar) +
 theme(legend.position = "bottom",
 plot.title = element_text(hjust = 0.5),
 legend.direction = "horizontal",
 text = element_text(family = "Merriweather", size = 15))


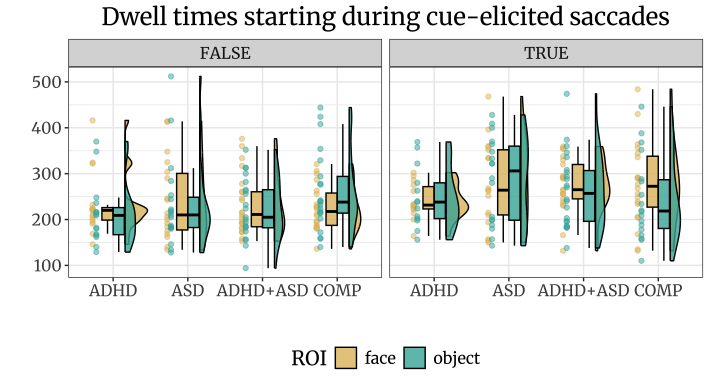

Supplement: nsaf112_Supplementary_Data [file nsaf112_supplementary_data.docx]
